# Phylogenetic and functional diverse ANME-1 thrive in Arctic hydrothermal vents

**DOI:** 10.1093/femsec/fiac117

**Published:** 2022-10-03

**Authors:** F Vulcano, C J Hahn, D Roerdink, H Dahle, E P Reeves, G Wegener, I H Steen, R Stokke

**Affiliations:** Department of Biological Sciences, Center for Deep Sea Research, University of Bergen, Bergen, Norway; Max-Plank Institute for Marine Microbiology, HGF MPG Joint Re­search Group for Deep-Sea Eco­logy and Tech­no­logy, Bremen, 28359, Germany; Department of Earth Science, Center for Deep Sea Research, University of Bergen, Bergen, Norway; Computational Biological Unit, Department of Informatics, Department of Biological Sciences, Center for Deep Sea Research, University of Bergen, Bergen, Norway; Department of Earth Science, Center for Deep Sea Research, University of Bergen, Bergen, Norway; Max-Plank Institute for Marine Microbiology, HGF MPG Joint Re­search Group for Deep-Sea Eco­logy and Tech­no­logy, Bremen, 28359, Germany; MARUM, Center for Marine Environmental Sciences, University Bremen, Bremen, 28359, Germany; Alfred Wegener Institute Helmholtz Center for Polar and Marine Research, Bremerhaven, 27570, Germany; Department of Biological Sciences, Center for Deep Sea Research, University of Bergen, Bergen, Norway; Department of Biological Sciences, Center for Deep Sea Research, University of Bergen, Bergen, Norway

**Keywords:** ANME-1, comparative genomics: thermophily, hydrothermal vents, phylogenomics

## Abstract

The methane-rich areas, the Loki's Castle vent field and the Jan Mayen vent field at the Arctic Mid Ocean Ridge (AMOR), host abundant niches for anaerobic methane-oxidizers, which are predominantly filled by members of the ANME-1. In this study, we used a metagenomic-based approach that revealed the presence of phylogenetic and functional different ANME-1 subgroups at AMOR, with heterogeneous distribution. Based on a common analysis of ANME-1 genomes from AMOR and other geographic locations, we observed that AMOR subgroups clustered with a vent-specific ANME-1 group that occurs solely at vents, and with a generalist ANME-1 group, with a mixed environmental origin. Generalist ANME-1 are enriched in genes coding for stress response and defense strategies, suggesting functional diversity among AMOR subgroups. ANME-1 encode a conserved energy metabolism, indicating strong adaptation to sulfate-methane-rich sediments in marine systems, which does not however prevent global dispersion. A deep branching family named *Ca*. Veteromethanophagaceae was identified. The basal position of vent-related ANME-1 in phylogenomic trees suggests that ANME-1 originated at hydrothermal vents. The heterogeneous and variable physicochemical conditions present in diffuse venting areas of hydrothermal fields could have favored the diversification of ANME-1 into lineages that can tolerate geochemical and environmental variations.

## Teaser

Phylogenomics and functional analysis of ANME-1 anaerobic methane oxidizers from hydrothermal systems at the Arctic Mid Ocean Ridge.

## Introduction

Three major groups of anaerobic methanotrophic archaea (ANME); ANME-1, ANME-2, and ANME-3 (Boetius et al. 2000, Knittel and Boetius 2009) mediate the anaerobic oxidation of methane (AOM). They reverse the methanogenesis pathway for methane oxidation (Hallam et al. 2004). Marine ANME archaea do not code for own respiratory pathways. Instead, they transfer the electrons liberated during AOM to sulfate-reducing bacteria (SRB) (McGlynn et al. 2015, Wegener et al. 2015). ANME appear globally in sulfate methane transition zone (SMTZ) of anoxic sediments and perform AOM in a wide range of physicochemical conditions (Hinrichs et al. 1999, Orphan et al. 2001, Knittel et al. 2005, Lloyd et al. 2006, Lösekann et al. 2007, Knittel and Boetius 2009, Roalkvam et al. 2011, Maignien et al. 2013, Ruff et al. 2013, Vigneron et al. 2013, Dowell et al. 2016, Ruff et al. 2016, Dombrowski et al. 2018). Among ANMEs, ANME-1 seem to be most widely distributed in thermal environments, colonizing both marine hydrothermal vents and terrestrial hot springs (Teske et al. 2002, Holler et al. 2011, Biddle et al. 2012, McKay et al. 2012, Borrel et al. 2019).

In AOM cultures from the Guaymas Basin hydrothermal sediments ANME-1 form partnership with the deep branching sulfate reducer *Candidatus* Desulfofervidus (Holler et al. 2011, Krukenberg et al. 2016). At low temperature environments like cold-seeps, ANME-1 grow with sulfate reducers of the SEEP-SRB clades (Kleindienst et al. 2012, Krukenberg et al. 2018). The mechanism for the exchange of reducing equivalents between the partner proceeds most likely through direct interspecies electron transfer (DIET) mediated by extracellular cytochromes and nanowires (Wegener et al. 2015, Skennerton et al. 2017, Krukenberg et al. 2018).

The recent-increased availability of genomes of ANME-1 have provided deep insights of their phylogeny, evolution, and metabolic properties. In the Genome Taxonomy Database (GTDB) (Rinke et al. 2021 and https://gtdb.ecogenomic.org/), ANME-1 (*Ca*. Methanophagales) is classified as a distinct order within the phylum *Halobacteriota* and the class *Syntropharchaeia*, separated from the other ANMEs (phylum *Halobacteriota*, class *Methanosarcinia*). Currently, the ANME-1 order includes the two families: ANME-1 and B39_G2. B39_G2 is affiliated to *Ca*. Alkanophagales (Wang et al. 2021, Wang et al. 2022). The ANME-1 family comprises 8 genera and 16 candidate species, whereas B39_G2 is represented by a single uncultured candidate species (Rinke et al. 2021; https://gtdb.ecogenomic.org). Besides ANME-1, the Syntropharchaeia class includes the two cultured species that oxidize the short-chain alkanes butane and propane, *Candidatus* Syntrophoarchaeum butanivorans and *Candidatus* Syntrophoarchaeum caldarius (Laso-Pérez et al. 2016). In addition, MAGs of the linage *Ca*. Alkanophagales, with the ANME-1 GTDB family B39_G2, describe a potential C_n_H_2n+2_ oxidizer. These MAGs encode a divergent Syntropharchaeum-like alkyl–coenzyme M reductase (ACR; Dombrowski et al. 2018) and a complete beta-oxidation pathway (Dong et al. 2020, Wang et al. 2021). In *Syntropharchaeia* multi-carbon metabolism seems to precede methane metabolisms. The latter capability likely appeared after the acquisition of a methane-oxidizing methyl-coenzyme M reductase (MCR) through horizontal gene transfer from the clades *Ca*. Methanofastidiosa/*Ca*. Nuwarchaeia (Borrel et al. 2019, Wang et al. 2021).

Besides few differences in the encoded MCR, all ANME-1 genomes have an identical set of enzymes for methane oxidation, with a conserved bypass of the Methylene-H_4_M(S)PT reductase (Mer) enzyme (Meyerdierks et al. 2010, Stokke et al. 2012, Krukenberg et al. 2018, Borrel et al. 2019, Wang et al. 2019). Little variability has also been observed in the redox complexes for energy conservation, with only a few genomes carrying the Na^+^-coupled respiratory *Rhodobacter* nitrogen fixation (Rnf) complex, in addition to F_420_H_2_ dehydrogenase (Fqo), heterodisulfide reductase (Hdr), F_420_-non-reducing hydrogenase (Mvh), formate dehydrogenase (Fdh) and DIET-supporting proteins (Borrel et al. 2019). Comparative genome analyses of ANME-1 have overall revealed a limited energy metabolism, highly specialized to catalyze AOM in SMTZs.

Efforts remain to understand how the genetic features of ANME genomes connect to the distribution of ANME in geochemically different niches. A comparative assessment of ANME-1 across their habitable environments would hence be useful to reveal their total genomic heterogeneity and possible genetic signatures for niche-specific microbial functions. In this study, MAGs of ANME-1 from focused and diffuse fluid flow sites at the Loki´s Castle vent field (LCVF) (Pedersen et al. 2010) and the Jan Mayen vent field (JMVF) (Stokke et al. 2020) at the Arctic Mid-Ocean Ridge (AMOR) were reconstructed. We identified ANME-1 lineages and studied their occurrence in various hydrothermal niches. Finally, with focus on vent taxa, we compared the functions encoded in the entire ANME-1 order.

## Materials and methods

### Environmental samples and DNA extraction

Genomic DNA was extracted from sediment samples collected in 2010, 2017, and 2018, from a white barite chimney section (BaCh2W), the superficial layer below a white microbial mat (BaCh4M), and a dark grey barite chimney base (BaCh3G) in the diffuse venting barite field at the Loki´s Castle vent field (Steen et al. 2016). The barite chimney samples included in this study were altogether named Loki´s Castle barite field chimneys. In 2018, a patch of sediment covered by a thick microbial mat was sampled with a blade corer, resulting in a 20 cm core. Likewise, the wall of a black smoker (Baumberger et al. 2016) was sub-sampled for DNA extraction. At the Jan Mayen vent field, *in situ* enrichments in the Bruse vent field sediments (Stokke et al. 2020) and F3 flange section of a white smoker from the Soria Moria vent field (Dahle et al. 2015) were sampled for DNA extraction. The samples are listed in Table 1. Total DNA was extracted using FastDNA^TM^SPIN Kit for Soil (MP Biomedicals, Santa Ana, CA, USA) according to manufacturer instructions and sequenced at the NSC Norwegian Sequencing Center in Oslo, except for the BaCh4M sequenced at StarSEQ in Mainz, Germany.

**Table 1.** Overview of samples from the Loki's Castle vent field (LCVF) and the Jan Mayen vent field (JMVF) included in this study.

Sample ID	Location	Type of sample	Description
LCBF* chimney (BaCh2W )	LCVF	Barite chimney	Middle section; white barite; ∼ 20 °C**; diffuse flow
LCBF chimney (BaCh4M)	LCVF	Barite chimney	Superficial layer below a white mat; 0–∼ 20 °C; diffuse flow
LCBF chimney (BaCh3G)	LCVF	Barite chimney	Chimney base; dark grey; ∼ 20 °C ; diffuse flow
LCBF* sediments	LCVF	Hydrothermal sediments	Sediments covered by *Sulfurimonas* mat; 20 cmbsf, dark grey; 10 °C ; diffuse flow
JMVF sediments	JMVF	Hydrothermal sediments	Bruse Vent Field; *in situ* incubators; 0–74 °C***; diffuse flow
LCVF black smoker (wall/bulk)	LCVF	Black smoker	João; two presumably high-temperature samples rich in sulfide minerals (a wall section (wall) and bulk material from the chimney (bulk); temperature unknown; focused flow
JMVF white smoker flange	JMVF	White smoker	Soria Moria; flange; 70–72 °C^****^; focused flow

*LCBF: Loki's Castle barite field; **(Steen et al. 2016); ***(Stokke et al. 2020); ^****^(Dahle et al. 2015)

### Geochemical analysis

For geochemical analysis, porewater from the blade corer was collected at 4°C with Rhizons (pore diameter, 0.2 µm). Alkalinity and hydrogen sulfide concentrations were measured onboard immediately after sampling, using a Metrohm 888 Titrando titrator and a Silver/Sulfide ionplus® Sure-Flow® Solid State Combination Ion Selective Electrode (ISE) (Thermo Scientific). Residual porewater was stored in 3% HNO_3_ acid-washed HDPE plastic bottles and frozen at −20°C for onshore for measurement of sulfate concentration (ICP-OES) (Eickmann et al. 2014).

At the Loki's Castle barite field, sediment temperatures were measured using the ROV arm equipped with a high-temperature probe hiT (WHOI MISO) (Fornari et al. 1998).

Moreover, at the Loki's Castle barite field the rates of methane oxidation and sulfate reduction were assessed in radiotracer assays with ^14^C-methane and ^35^S-Sulfate as described by Wegener et al. 2008. Sediments were supplemented with anoxic medium (Laso-Pérez et al. 2018) and aliquoted in replicates in exetainer vials under anoxic conditions. The headspace was filled with gaseous hydrocarbons-equilibrated sterile medium (methane, ethane, propane, and butane). After addition of the radiotracers, the incubation was stopped after 48 h at room temperature. The radio-labelled reaction products were collected through chromium distillation (for ^35^S-Sulfide) fraction (Kallmeyer et al. 2004), or using a Phenylethylamine trap (for^14^C-CO_2_) and the associated radioactivity measured for metabolic rates estimation.

### Catalyzed reported deposition fluorescence atalyzedhybridization (CARD-FISH)

Onboard, 1 g of material from barite chimneys and surrounding sediments was resuspended in 50 ml of 1×PBS (Phosphate-Buffered Saline) and fixed overnight at 4°C in 2% formaldehyde. Samples were centrifuged 15 min at 1000 × *g* at 4°C with a swing rotor to allow sediments to settle. Aliquots of the resulting supernatant were filtered on isopore polycarbonate filters (0.2 μm pore diameter, Merck Millipore). Filters were washed twice with 1× PBS pH 7.6 and stored at −20°C.

Onshore,*in situ*hybridization of rRNA with horseradish peroxidase (HRP)-labeled oligonucleotide coupled to catalyzed reporter (tyramide) deposition (Pernthaler and Amann 2004, Amann and Fuchs 2008) was performed. Briefly, filters were coated with 0.1% (w/v) low-gelling point agarose. Permeabilization of bacterial and archaeal cell walls was performed by incubation for 60 min at 37°C in lysozyme solution (10 mg/ml lysozyme in 1×PBS pH 7.6, 0.05 M EDTA pH 8.0, 0.1 M Tris-HCl pH 8.0) and incubation for 5 min at room temperature in proteinase K solution (15 μg/ml proteinase K in 10 mM Tris-HCl pH 8.0, 1 mM EDTA pH 8.0), respectively. Endogenous peroxidases were inactivated by incubating the filters in 0.15% H_2_O_2_ solution in methanol for 30 min at room temperature. Hybridization of rRNA was performed by incubating the filters for 2 h at 46°C in a solution 1:300 of HRP-labelled probes (8 pmol/μl working solution) and hybridization buffer (900 mM NaCl, 20 mM Tris-HCl pH 8.0, 1×blocking reagent (Roche), 10% dextrane sulfate, 0.02% sodium dodecyl sulfate (SDS) and probe-specific formamide %) in humidified hybridization chambers. After 15 min washing at 48°C in preheated washing buffer (5 mM EDTA pH 8.0, 20 mM Tris-HCl pH 8.0, 0.01% SDS and NaCl according to formamide concentration in hybridization buffer), filters were washed again in 1×PBS pH 7.6 for 15 min. Filters were incubated at 46°C for 45 min in humidified chambers in a solution 1000:10:1 of amplification buffer (2 M NaCl, 1× PBS pH 7.6, 0.1× Blocking Reagent (Roche), 10% dextran sulfate), 0.15% H_2_O_2_ solution (5 μl of 30% H2O2 in 1 ml 1× PBS pH 7.6) and fluorescently-labeled tyramides (Alexa488 or Alexa594), for signal amplification. Washing in 1× PBS pH 7.6 was followed by DNA staining by incubation of filters in (DAPI 4’,6′- diamino-2-phenylindole) solution (1 μg/ml) for 10 min at room temperature. Finally, filters were mounted on glass slides using Citifluor Mountant Solution: VECTASHIELD® Antifade Mounting Medium (Vector Laboratories). After the first amplification step, filters for double hybridization were treated with an additional step of peroxidase inactivation in 0.15% H_2_O_2_ methanol solution. Filters were finally analyzed with epifluorescent microscopy using an Axiophot II imaging microscope (Zeiss; Germany). The probes used in this study are listed in Table S1.

### Assembly, binning, and annotation

Metagenome assembly for all samples followed the procedure described for the Bruse vent field (Fredriksen et al. 2019). In short, filtering of raw Illumina MiSeq 300 paired-end reads, and assembly, were performed using the CLC genomics workbench (Qiagen, v.10–12) using default parameters (quality 0.05; length, minimum 40, and maximum 1000 nucleotides). In addition, one nucleotide was removed from terminal read ends. Assembly was performed using default parameters with an automatic k-mer size and bubble size. A minimum contig length was set to 1000 bases with scaffolding enabled.

Except for the Loki's Castle barite field sediments sample, MAGs were reconstructed using MetaBat (Kang et al. 2015). MAGs from Loki's Castle barite field sediments were reconstructed using a combination of MetaBat 2.15, MaxBin v.2.2.7 (Wu et al. 2016), concoct 1.1.0 (Alneberg et al. 2014), and DAS Tool (Sieber et al. 2018). For MAGs Chimney19_Bin_00 366 and Chimney19_MAG_00 329, first a co-assembly was done with MEGAHIT (Li et al. 2015), then automatic binning was performed using again concoct and MetaBat. Reference genomes were downloaded from the Assembly database at NCBI (April 2020/May 2021). Contamination and completeness of the individual MAGs and of the reference genomes in the current study were assessed on the presence of lineage-specific, conserved single-copy marker genes using CheckM v1.0.7 (Parks et al. 2015). Functional annotation of MAGs and downloaded genomes were performed within the anvi'o (v6.2 and v.7) pipeline (Eren et al. 2021). The predicted coding sequences (Prodigal v2.6.3, February 2016) (Hyatt et al. 2010) were annotated against the following HMM profiles using scripts within anvi'o: Archaea_76 (Lee 2019), Ribosomal_RNAs (Seemann T, https://github.com/tseemann/barrnap), the Pfam database v 32.0 (2018–08), the COG database (Galperin *et al*. 2015) using DIAMOND as search algorithm (v 0.9.14) (Buchfink et al. 2014), and search against the KOfam HMM database (Aramaki et al. 2020). In addition, for each contig database, amino acid sequences were exported, annotated with GhostKoala (default parameters) (Kanehisa et al. 2016), and imported back into anvi'o (Graham, https://merenlab.org/2018/01/17/importing-ghostkoala-annotations/).

### Estimates of relative abundances of ANME archaea

Phylogenetic composition and abundance for each metagenome were first assessed by the assembly of SSU sequences with phyloFlash (Gruber-Vodicka et al. 2020, https://github.com/HRGV/phyloFlash). Furthermore, filtered reads were mapped against all contigs using BBMap v.Feb.2020 (Bushnell B.—sourceforge.net/projects/bbmap/) with default parameters. The relative abundance of each MAG was calculated using the -coverage and -profile commands in CheckM v1.0.7 (Parks et al. 2015) using the BBMap mapping file.

### Taxonomic classification, phylogenetic and phylogenomic analysis

Classification of MAGs was performed using the GTDB toolkit (GTDB-Tk) (Chaumeil et al. 2020) and the GTDB version R06-RS202 (Parks et al. 2018, Parks et al. 2021).

Amino acid sequences from 35 selected single-copy marker genes (Table S2), identified from the HMM profile Archaea_76 (Lee 2019) in anvi'o, were extracted from the ANME-1 AMOR MAGs and 384 reference genomes publicly available at NCBI (reference genomes were selected based on Borrel et al. 2019, Hahn et al. 2020, Schwank et al. 2019). The extracted single-copy marker genes were aligned using MAFFT L-INS-i v7.397 (2018/Apr/16) (Katoh 2002), trimmed with TrimAL (TrimAL v 1.4. rev15, -gappyout) and concatenated with catfasta2phyml (https://github.com/nylander/catfasta2phyml/blob/master/catfasta2phyml.pl). A maximum-likelihood tree of the concatenated sequences was calculated with IQ-TREE multicore version 1.6.7 with LG+F+R10 model and 1000 bootstraps. The ANI of AMOR ANME-1 genomes and references was calculated using anvi'o integrated PyANI v.0. 2. 7 (Pritchard et al. 2016). For phylogeny based on the subunit A of methyl-coenzyme M reductase (McrA), all MAGs were screened for the McrA protein sequences against the HMM profile for KEGG orthology ID K00399 available at https://data.ace.uq.edu.au/public/graftm/7/ (7.27.methyl_coenzyme_reductase_alpha_subunit.mcrA.gpkg.tar.gz (09-Aug-2017)) (Boyd et al. 2018), as exemplified in anvi'o pipeline by Lee (https://merenlab.org/2016/05/21/archaeal-single-copy-genes/). Identified McrA sequences were extracted from the contig databases (https://merenlab.org/2016/05/21/archaeal-single-copy-genes/) and aligned with MAFFT v7.397 (2018/Apr/16) (Katoh *et al*., 2002) using the G-INS-i iterative refinement method, and gaps removed using TrimAL v 1.4. rev15 with the gappyout option (Capella-Gutiérrez *et al*. 2009). Finally, the phylogenetic tree was calculated with IQ-TREE v 1.6.12 (Nguyen et al. 2015) model LG+F+R6 and 1000 bootstraps. An identical procedure was followed for phylogenetic analysis of the16S rRNA gene. When available, 16S rRNA sequences were extracted from the MAGs using anvi'o (v6) (–hmm-source Ribosomal_RNAs –gene Archaeal_16S_rRNA). A list of sequences used in the 16S rRNA phylogeny and MAGs which 16S rRNA genes were extracted is given in Table S3A and B. Reference sequences were selected based on Teske et al. 2002, Knittel et al. 2005, Lösekann et al. 2007, Biddle et al. 2012).

A complex pangenome, representing 38 genomes with >70% completeness and <10% contamination, was re-constructed using the anvi'o workflow for microbial pangenomics (https://merenlab.org/2016/11/08/pangenomics-v2/#displaying-the-pan-genome). Singletons were removed with the option ‘—min-occurrence 2’ to simplify the pangenome visualization. Organization of the pangenome of the assembled genomes was based on presence-absence of groups of genes with homologous amino acid sequence (gene clusters) (Shaiber et al. 2020). From this, a dendrogram was re-constructed representing the hierarchical clustering based on gene cluster frequency (Delmont and Eren 2018). Functional enrichment analysis was performed using anvi'o v6 program anvi-compute-functional-enrichment. Functions were considered enriched for q-values < 0.05 based on Shaiber *et al*. 2020.

## Results

### Distribution and morphology of ANME-1 under different environmental settings

To resolve the genomic diversity of ANME-1 in hydrothermal vents, we performed a metagenome-based study focusing on two methane-enriched hydrothermal vents systems, the Jan Mayen vent field and the Loki's Castle vent field located on the Arctic Mid-Ocean Ridge. The analyzed samples cover a wide diversity of hydrothermal settings, including various niches in the Loki's Castle barite field. This is a low-temperature diffuse flow area, situated approximately 50 meters apart from the Loki's Castle black smoker, characterized by venting of hydrothermal fluids through sediments and barite chimneys (Steen et al. 2016) (Table 1). When 16S rRNA gene sequences were retrieved from the metagenomic dataset, ANME were detected in all samples. They remained either taxonomically unassigned or assigned to ANME-1a. Estimated relative abundances of ANME-1 varied considerably between the samples (Fig. S1A) and reflected differences in fluid flow rates and in end-member fluid concentration of methane between and within the two vent fields (Baumberger et al. 2016, Steen et al. 2016, Dahle et al. 2018, Stokke et al. 2020). In the Jan Mayen vent field, where an endmember fluid concentration of 5.4 mmol kg^–1^ of methane was measured (Dahle et al. 2018, Stokke et al. 2020), ANME-1 reach a relative abundance between 5 and 14% in diffuse venting sediments. In the flange of a white smoker the relative abundance of ANME-1 16S rRNA gene was approximately 10% (Fig. S1A).

The highest relative abundance of ANME-1 was observed in the high-temperature venting black smoker in the Loki´s Castle vent field consistent with higher endmember fluid concentration of methane of 12-13 mmol kg^−1^ methane (Baumberger et al. 2016). End-member fluids are highly diluted in the diffuse-flow barite field in the Loki´s Castle. Nevertheless, the sediments hosted an abundant population of ANME-1, indicating high flowrates of methane. Consistently, a steep temperature gradient and a shallow SMTZ were observed (2–4 cmbsf) (Fig. S1B). Moreover, methane oxidation rates of 110 nmol d^−1^ g_(wetweight; ww)_^−1^ and a methane dependent sulfate-reduction rate (SRR) of 30 nmol d^−1^ g_ww_^−1^ respectively, were measured (Fig. S1C). The lowest relative abundance of ANME-1 was observed in the barite chimneys at Loki's Castle barite field (Fig. S1A).

**Figure 1.**
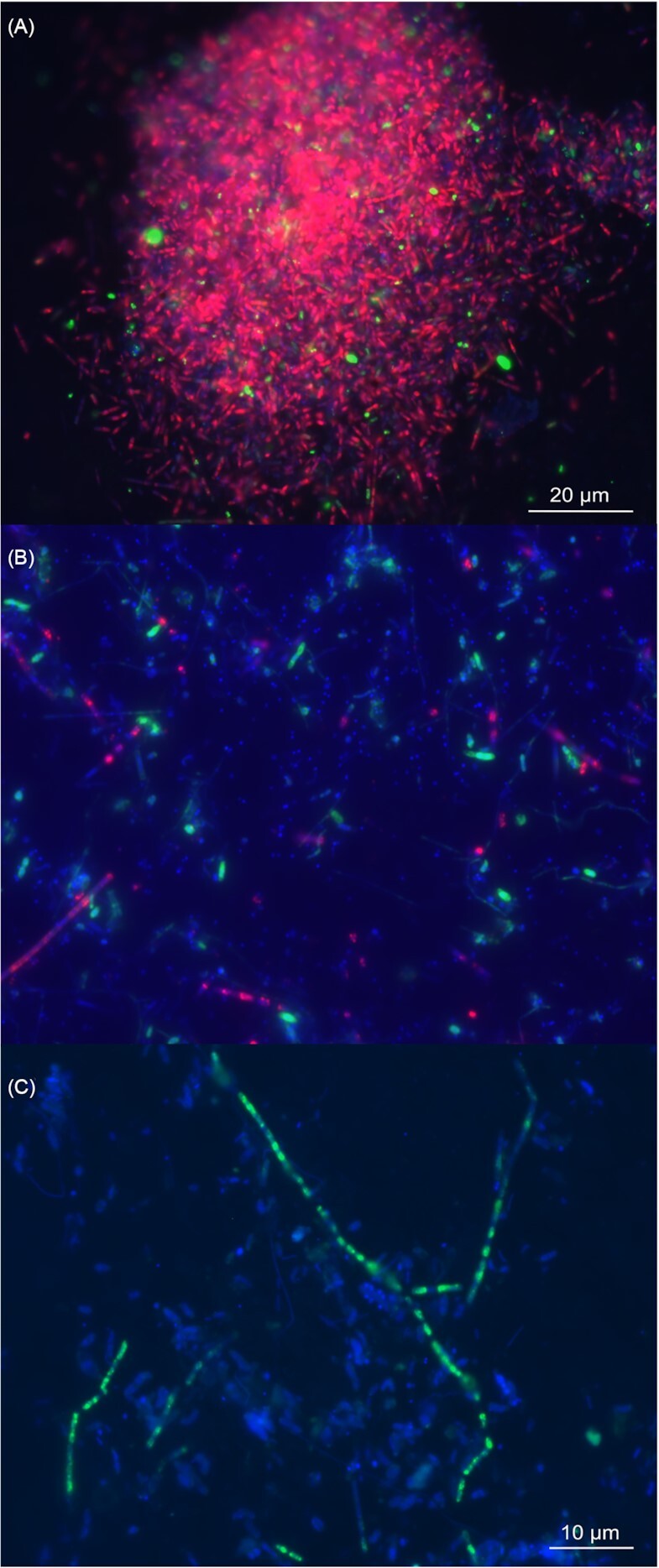
Micrographs of ANME-1 and partner bacteria of the Loki's Castle barite field. **(A)** Aggregates of ANME-1 (ANME-1–350 probe) and Deltaproteobacteria (Delta495 probes) in Loki's Castle barite field sediments and **(B)** barite chimneys. ANME-1 and Deltaproteobacteria are in red and green, respectively. **(C)** Filaments of ANME-1 in the upper section of a barite chimney, stained in green. Scale bars are reported for A and B-C.

We visualized ANME-1 and their partners from different locations using CARD-FISH. In sediments, rod-shaped ANME-1 and Deltaproteobacteria form well-mixed large aggregates with diameters between 40 and 80 µm of (Fig. 1A). In the barite chimneys, the few ANME-1 appeared in short chains of 2 to 10 cells (Fig. 1B). ANME-1 rods and Deltaproteobacteria were loose within a matrix of mineral particles. Occasionally, ANME-1 cells formed filaments with a length of up to 100 µm in the external layers of the barite chimneys (Fig. 1C). This morphology resembled the chain-forming aggregates described in 50 °C enrichments of ANME-1-Guaymas/SRB (Holler et al. 2011). Notably, *Ca*. Desulfofervidus was observed in the barite field sediments at 10°C (Fig. S1A).

### Taxonomy and distribution of ANME-1 archaea

**Figure 2.**
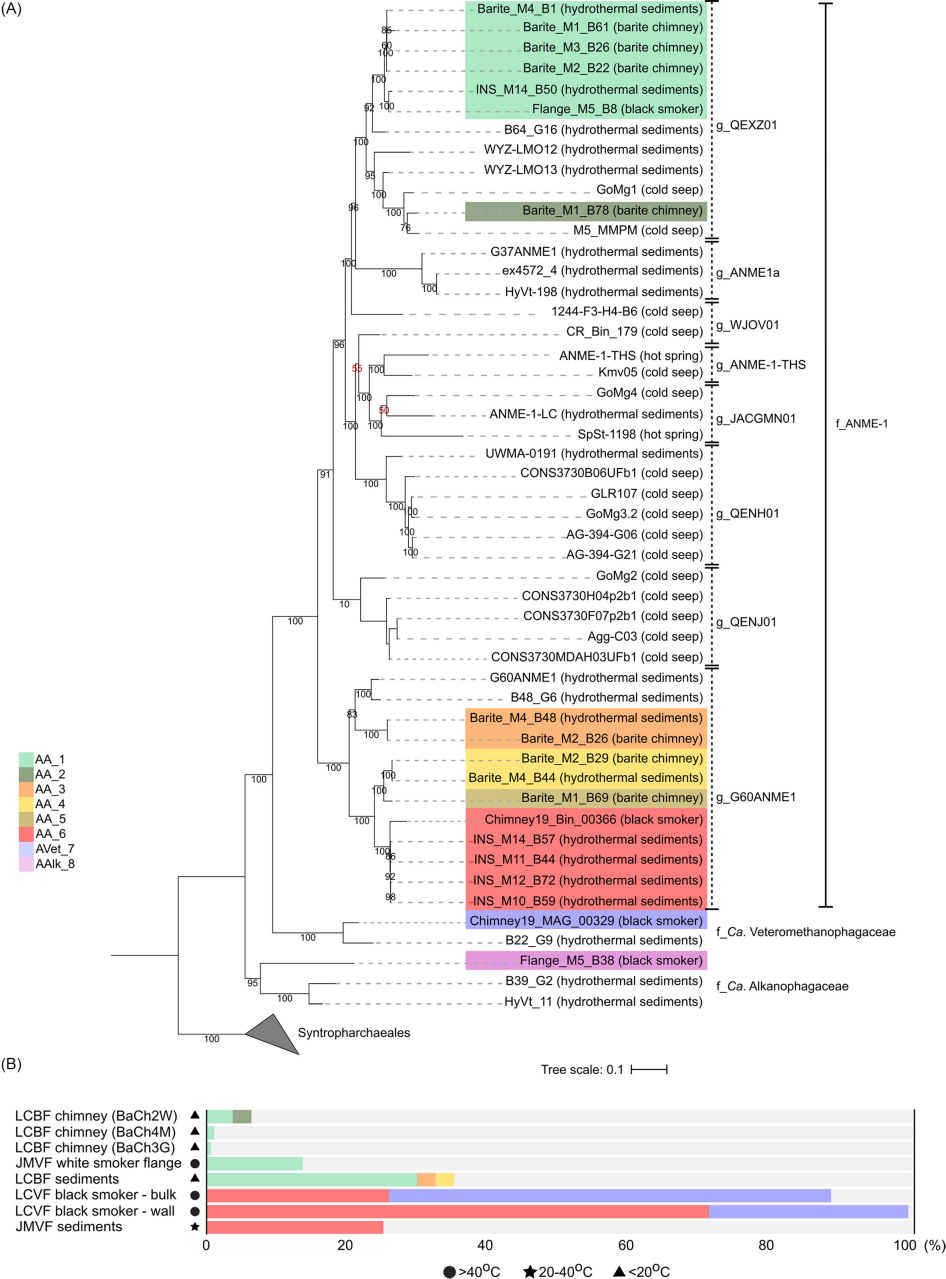
Phylogenomic analysis of ANME-1. **(A)** Phylogenomic tree of the ANME-1 order based on concatenated alignment of 35 marker genes. The ANI-defined AMOR subgroups are highlighted by colors as in the legend. For each genome, the environment of origin is indicated in parenthesis next to the leaf name. The genus- and family- level classification from GTDB-tk is indicated on the right (g_ for genus; f_ for family). Bootstrap values < 60 are in red. **(B)** Relative abundance of the AMOR subgroups in various samples at Loki's Castle vent field and Jan Mayen vent field. The estimated temperature at each site is indicated by symbols. ‘LCBF’: Loki's Castle barite field, ‘JMVF’: Jan Mayen vent field, ‘LCVF’: Loki's Castle vent field.

In total we reconstructed 19 ANME-1 related MAGs (Table S4B). Three from the barite field sediments, seven from barite chimneys and two from the black smoker were found at Loki´s Castle vent field. From the Jan Mayen vent field, five MAGs from sediments and two from the flange were obtained (for details see Table 1). The MAGs were on average 83% complete and showed low contamination values (<2.6%, 0.65% on average) (Table S4C). Our phylogenomic analysis identified three families in the ANME-1 order (Fig. 2A). These were of the classical ANME-1 which included the clusters ANME-1a and ANME-1b (Knittel et al. 2005) (Fig. S2) and *Ca*. Alkanophagaceae (Wang et al. 2021). The third represented a novel deep branching family, that we named *Ca*. Veteromethanophagaceae. The name stands for ‘old methane consumer’: *vetero-*, old (Latin); *methano-*, pertaining to methane (new Latin); *phagaceae*, eating (Greek). The topology of the phylogenomic tree was overall consistent with the 16S rRNA and McrA gene phylogenies (Fig. S2 and Fig. S3).

Out of the eight identified ANME-1 genera, our reconstructed MAGs in the ANME-1 family affiliated either with the genus QEXZ01 (7) or with the genus G60ANME1 (11) (Fig. 2A and Table S4C). Among them, six species-level subgroups were defined based on pairwise average nucleotide identity (ANI) (Fig. S4 and Table S5). They were named AMOR ANME-1 (AA) subgroups (AA_1 to AA_6) where subgroups AA_1 and AA_2 were of genus QEXZ01 and subgroups AA_3 to AA_6 of genus G60ANME1(Fig. 2A and Table S5). Subgroups AVet_7 and AAlk_8 were identified within *Ca*. Veteromethanophagaceae and *Ca*. Alkanophagaceae, respectively (Fig. 2A and Table S5).

ANME-1 genera showed differences in their geographic origin and distribution. Based on our analysis, the genus G60ANME1 clustered with genomes exclusively from marine hydrothermal vents. The genus G60ANME1 was originally named after a MAG assembled from a 60 °C AOM culture from the Guaymas Basin vent system (Krukenberg et al. 2018). The genus QEXZ01, from hydrothermal vents located at AMOR, also grouped with genomes from the Guaymas Basin vent system, the cold seeps in the Gulf of Mexico and from marine sediments of Aarhus Bay. Genomes, exclusively of hydrothermal origin (Guaymas Basin) were observed in genus ANME-1a. The genera WJOV01 and QENJ01 included only genomes from marine cold seeps. QENH01, JACGMN01 and ANME-1-THS included genomes with a mixed provenance. Notably, the genera ANME-1-THS and JACGMN01 contained genomes from terrestrial hot springs, marine cold seeps, and alkaline vent fluids. Altogether, most ANME-1 genera seemed to have a wide geographic distribution, which argues for their large adaptability to diverse environmental conditions. Some genera seemed, however, restricted to a specific type of environment or geographic location.

On a local scale, at the Arctic Mid Ocean Ridge, the AMOR subgroups showed heterogeneity in their abundance and distribution within and between the hydrothermal vent fields (Fig. 2B). In the Loki's Castle barite field, we found five of the six ANME-1 subgroups (AA_1–AA_5). All five were detected in barite chimneys, although in low relative abundances. The barite field sediments hosted three subgroups (AA_1, AA_3, AA_4) of which AA_1 dominated with up 40% of the total community. The high-temperature black smoker at Loki's Castle hosted only the AA_6 subgroup, but this represented up to 73% of the total microbial community. Notably, the wall and the bulk sample from the of the black smoker chimney hosted the subgroup of the *Ca*. Veteromethanophagaceae, AVet_7. At Jan Mayen vent field, only two of the six ANME-1 subgroups were observed. AA_6 occurred in the temperate sediments at approximately 25% rel. abundance. AA_1 occurred in the flange with a rel. abundance of 14%. Notably, the flange also hosted the subgroup of *Ca*. Alkanophagaceae, AAlk_8, in low abundances (0.24%.).

### Comparative genomics of ANME-1

To further explain the observed phylogenetic diversity and the wide adaptability of ANME-1 to diverse environmental conditions, we analyzed their genomic content. Based on functional annotation against the KOfam HMM database, all ANME-1 MAGs from AMOR, including the new family *Ca*. Veteromethanophagaceae encode very similar metabolic pathways. This included genes of the reverse methanogenesis pathway, redox complexes, and the enzymes of the reverse acetyl-CoA pathway (Table S6A and B) (Meyerdierks et al. 2010, Stokke et al. 2012, Krukenberg et al. 2018). They all showed the potential for DIET as they coded for multiple multi-heme cytochromes of the kind that was expressed in consortia-forming ANME-1 cultures (Fig. S5) (Wegener et al. 2015, Krukenberg et al. 2018). Even the amino acid, cofactors and vitamin metabolisms were conserved (Table S7).

The AAlk_8 appeared as a multi-carbon degrader, as it encoded a divergent Syntropharchaeum-like McrA, all genes for beta-oxidation and Mer (Table S6B and Fig. S6), a complete Mvh and lacked cytochromes (Dong et al. 2020; Wang et al. 2021).

**Figure 3.**
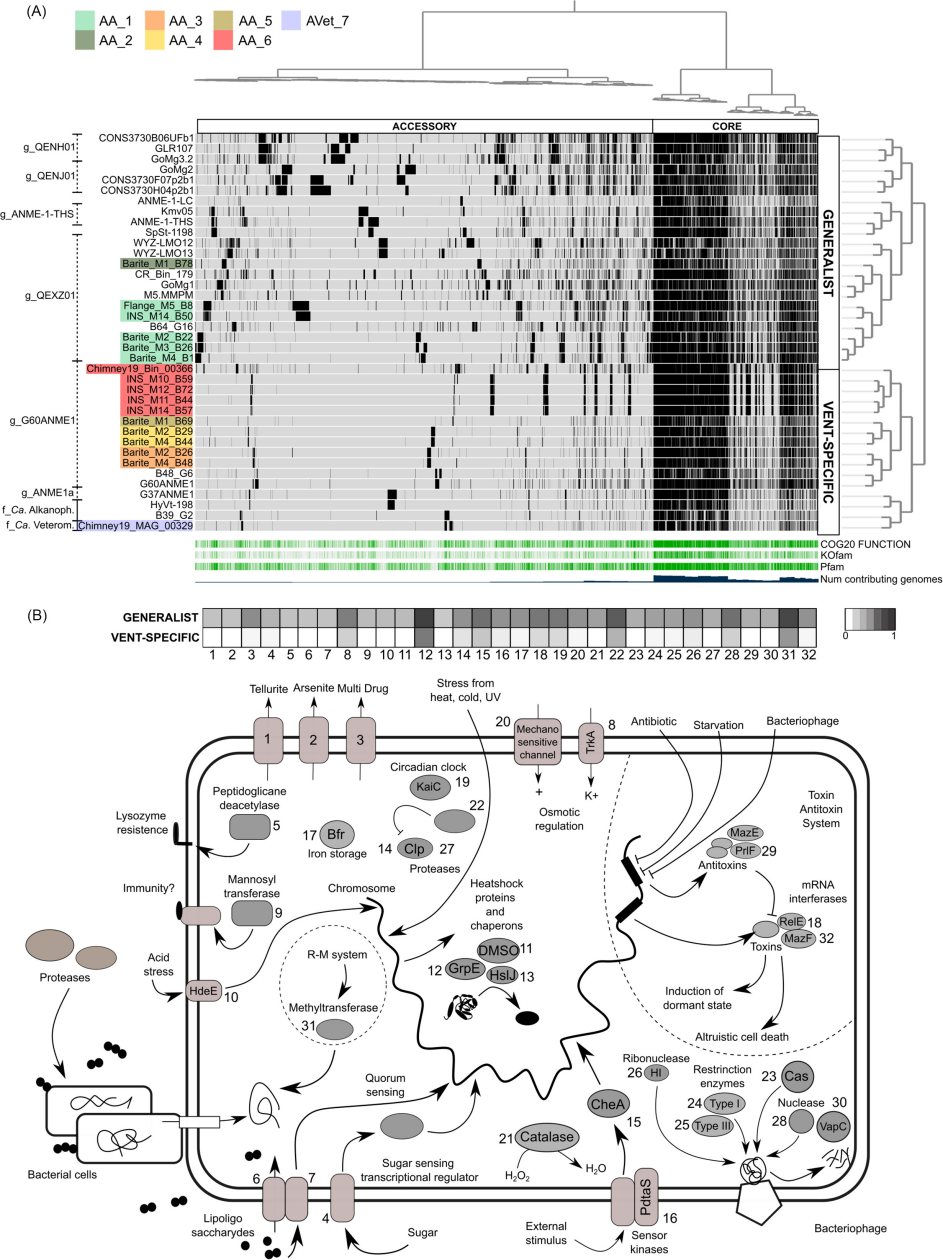
Gene clusters enriched in generalist ANME-1 and their cellular function. **(A)** Pangenome of the ANME-1 order. Hierarchical clustering is expressed by the dendrogram on the left of the phylogram. Genomes are thereafter divided into generalist and vent-specific. The core and the accessory pangenome are indicated. The coverage of COG, KOfam and Pfam annotations and the number of genomes contributing to each gene cluster are given below the phylogram. GTDB-tk classification is on the right. **(B)** Diagram of the cellular function of 32 genes enriched in generalist ANME-1. The occurrence of each gene in generalists and vent-specific genomes is indicated in the heatmap. More details of each cellular function are given in Table S8.

To further compare ANME-1 genomes based on their overall genome content, a pangenome analysis was performed (Fig. 3A). The pangenome consisting of 64264 genes was organized into in 6058 gene clusters (Delmont et al. 2018). The core pangenome comprised 1604 gene clusters (46147 genes), while the accessory pangenome consisted of 4454 gene clusters (18117 genes).

When the ANME-1 genomes were hierarchically clustered based on their similarity in gene cluster frequency, the resulting dendrogram identified two major functional groups of genomes (Fig. 3A). Based on habitat of origin, they were defined as vent-specific and generalist ANME-1. The vent-specific group consisted of genomes reconstructed only from hydrothermal vents and included genus G60ANME1 (AA_3, AA_4, AA_5 and AA_6), genus ANME1a, and the families *Ca*. Veteromethanophagaceae, as well as the multi-carbon degrading *Ca*. Alkanophagaceae. In contrast, the generalist group consisted of genomes reconstructed from geochemically heterogeneous environments like marine cold seeps, vents, and terrestrial hot springs. It included genera QEXZ01 (AA_1 and AA_2), ANME-1-THS, JACGMN01, QENH01, QENJ01, and WJOV01.

Functional differences between the two groups were analyzed using the functional enrichment analysis of anvi'o (Shaiber et al. 2020). In the generalist group 89 genes were enriched. Of these 53 were assigned to COG categories and included inorganic ion transport and metabolism (P), posttranslational modification, protein turnover, chaperones (O), signal transduction mechanisms (T), replication, recombination, and repair (L), cell wall, membrane, and envelope biogenesis (M) and translation (J) (Table S8). Enriched genes encoded processes involved in the response to chemical gradients, pH, and hydrogen peroxide, osmotic stress regulation and detoxification of arsenic and tellurium (Fig. 3B). Moreover, they coded for transporters of nutrients, zinc, xenobiotics, and phosphate and for iron storage proteins (Fig. 3B). Finally, genes regulating the cellular physiology in response to pathogens and starvation were enriched. These included multiple mRNA interferases of the type I and II Toxin Antitoxin system (TA), typically regulating the cellular stress response (Fig. 3B). The vent-specific ANME-1 showed few enriched functions, only few that could be linked to the thermal stability of tRNA and the cellular membrane (Table S8).

## Discussion

### Hydrothermal vents host phylogenetically and functionally divergent ANME-1

Our comparative genomic study detailed ANME-1 genomic diversity in the Loki´s Caste vent field and Jan Mayen vent field. Eight phylogenetically distinct AMOR subgroups were defined. Besides the six that belonged to the ANME-1 family, two affiliated with deep branching lineages in the ANME-1 order, one with the *Ca*. Veteromethanophagaceae, and one with *Ca*. Alkanophagaceae, a putative multi-carbon degrader. Lineages of the ANME-1 family and *Ca*. Veteromethanophagaceae encode a set of metabolic enzymes. This indicates that despite dwelling in different geochemical setting (focused flow of black smokers and diffuse low-temperature in the barite field), ANME-1 and *Ca*. Veteromethanophagaceae systematically rely only on methane and syntrophic associations with sulfate reducers. The ANME-1 from hydrothermal vents are either vent-specific or generalists. The vent-specific ANME-1 cluster rather in the root of the ANME-1 phylogenetic tree (Wang et al. 2022). Such distribution suggests a hydrothermal and a thermophilic (Wang et al. 2022) origin of the ANME-1 order. The vent specific ANME-1 appeared limited in their encoded functional capacity. Instead, the generalists that appear also at cold-seeps and terrestrial environments encode more genes for stress response, detoxification, and defense mechanisms.

In the barite field of the methane-rich Loki's Castle vent field, the occurrence of cold seep-adapted generalist could be driven by its cold seeps-like biogeochemical environment (Pedersen et al. 2010), in close proximity to black smokers. Diluted hydrothermal fluids allow the settlement of siboglinid tube worms, typical at cold seeps (Pedersen et al. 2010). Furthermore, shallow SMTZs (Fig. S1B) are typically observed at seeps, under mats of sulfur-oxidizers (Orphan et al. 2001, de Beer et al. 2006, Lloyd et al. 2006, Roalkvam et al. 2011, Gründger et al. 2019, Carrier et al. 2020). The availability of cold seep-like niches might favor the establishment through genetic selection of generalist lineages that can colonize lower temperature environments, next to vent-specific lineages. Overall, the exposure to the high physicochemical diversity found in deep-sea hydrothermal vents like the Loki's Castle vent field could fuel such diversification of the resident ANME-1 population, on a phylogenetic and functional level. This might have happened in the later stages of ANME-1 evolution, given the likely hydrothermal origin of ANME-1. The acquisition of genetic systems for defense and stress control might have prompted their ability to disperse in cold seeps and other habitats.

### ANME-1 lineages can spread and colonize distant geographic locations

According to the generally accepted theory of Beijerinck and Baas Becking (Baas-Becking 1934), microbial organisms are globally distributed, and locally selected by the environment. Recent studies have shown that Beijerinck's theory is applicable to deep-sea hydrothermal microbes (Dick 2019), and sequences belonging to members of the hydrothermal microbiome have been found in open ocean waters (Gonnella et al. 2016). It is not clear how strict anaerobes like ANME-1 could freely disperse in the water column and still be viable and able to colonize geographically distant areas. Nevertheless, phylogenetic evidence supports connectivity between geographically distant sites, such as AMOR and the Guaymas Basin vent field. The genus QENH01 appears at the Hydrate Ridge (Pacific Ocean), Hikurangi Margin (Pacific Ocean), and Gulf of Mexico (Atlantic Ocean). Such extensive biogeographic distance could be explained by the global deep ocean circulation (Talley 2013). Importantly, the deep ocean remained anoxic until well after the Great Oxygenation Event (2 Gyr) (Canfield 1998) and later experienced anoxic episodes (Jenkyns 2010), which may have promoted ANME-1 dispersal. In today's oxic ocean, ANME-1 could travel in a dormant state, as suggested for microaerophilic Campylobacterota and Aquificales (Gonnella et al. 2016), or could be transported in anoxic microniches. Connectivity likely exists between marine and terrestrial environments. *Ca*. Methanoalium (ANME-1-THS and JACGMN01) was initially defined as a ‘land’ clade after the reconstruction of ANME-1-THS from a Tibetan Hot Spring (Borrel et al. 2019). Chadwick's (Chadwick et al. 2022) and our study expanded this clade with additional genomes from a hot spring in California (SpSt_1198), a marine cold seep in the Gulf of Mexico (GoMg4), the Lost City alkaline vent on the Atlantic Massif (ANME-1-LC), and a terrestrial mud volcano located close to the coast of the Black Sea (Kmv05). Further genomic analysis is required to fully decipher the physiological mechanisms at the basis of ANME-1 phylogenetic/functional diversification and dispersal, such as dynamics of the horizontal gene transfer processes and genetic systems for sporulation and induction of dormancy.

## Conclusions

Overall, our metagenomic approach targeting a wide spectrum of hydrothermal settings in the Loki´s Castle and the Jan Mayen vent fields, allowed us to propose that hydrothermal vents, characterized by geochemical and thermal heterogeneity, could fuel ANME-1 phylogenetic and functional diversification, acting as evolutionary hotspots. Furthermore, they may have promoted the divergence between vent-specific and generalist ANME-1. Despite ANME-1 capacity to disperse globally, marine ANME-1 are overall characterized by metabolic homogeneity and are well adapted to SMTZs. Notably, yet the still small sample size might underestimate their distribution. Further genomic studies are required to complement ANME-1 taxonomy, to confirm the observed functional groups and to determine how selective advantage mechanisms and horizontal gene transfer have shaped ANME-1 lineages through time.

## Supplementary Material

fiac117_Supplemental_Files
