# Supplementary material for: Phylogenetic and functional diverse ANME-1 thrive in Arctic hydrothermal vents
Source: FEMS Microbiol Ecol. 2022 Oct 3;98(11):fiac117. doi: 10.1093/femsec/fiac117 (PMC9576274; doi:10.1093/femsec/fiac117)
Supplement: fiac117_Supplemental_Files [file fiac117_supplemental_files.zip › ANME1_REVISED_Supp_data_CAPTIONS_FIGURES.docx]

**Phylogenetic and functional diverse ANME-1 thrive in Arctic hydrothermal vents**

Vulcano F^1*^, Hahn CJ^4^, Roerdink D^2^, Dahle H^3^, Reeves EP^2^, Wegener G^4,5,6^, Steen IH^1^, and Stokke R^1^

**Supplementary Captions**

**Supplementary Figure 1.** **relative abundance of ANME-1 and syntrophic partners at Loki’s Castle vent field (LCVF) and Jan Mayen vent field (JMVF) and geochemistry of Loki’s Castle barite field (LCBF) sediments.** (A) Relative abundances of ANME-1a and b, unclassified ANME, ANME-2 and putative sulfate-reducing partners, based on phyloFlash-filtered 16S rRNA genes from metagenomes. (B) Geochemical and temperature profiles in the barite field sediments. The sampled horizon is indicated by a red star. The SMTZ is indicated in grey. (C) AOM rates estimated with ^14^C-methane and ^35^S-Sulfate in the barite sediments. Ethane, propane, and butane were also tested as substrates.

**Supplementary Figure 2**. **16S rRNA gene phylogeny.** Where available, 16S rRNA gene sequences were extracted from MAGs and compared to ANME-1b and ANME-1a sequences from Guaymas Basin and the Black Sea. The AMOR subgroups are highlighted by colors as in the legend. For details about AMOR subgroups see Supplementary Figure 4.

**Supplementary Figure 3. Comparison between McrA phylogeny (left) and concatenated phylogeny (right).** Bootstrap values are reported when lower than 90%. ANME-1 genera according to GTDB-tk classification are represented by colors.

**Supplementary Figure 4. (A)** ANI of the 50 MAGs included in the study. AMOR MAGs with >95% ANI are clustered into the AMOR subgroups as indicated by the legend. Percentage identity values are indicated by the grey gradient.

**Supplementary Figure 5**. **Distribution and abundance of cytochromes in ANME-1 order.** The number of genes annotated with cytochrome-associated Pfams in indicated by the gradient. The AMOR subgroups and the GTDB-tk genera are given. Pfam accession numbers correspond to the following annotations: (1) Cytochrome c7 and related cytochrome c; (2) NapC/NirT cytochrome c family, N-terminal region; (3) Cytochrome c3; (4) Cytochrome c554 and c-prime; (5) Class III cytochrome C family; (6) Cytochrome c-type biogenesis protein CcmF C-terminal; (7) Doubled CXXCH motif (Paired_CXXCH_1).

**Supplementary Figure 6**. **Distribution of the genes of the beta-oxidation pathway in the ANME-1 order, Syntropharchaeum, ANME-2 and ethane-oxidizers**. The color gradient indicates the copy number of each gene. The AMOR subgroups and the GTDB-tk classification are indicated. Annotation is based on GhostKoala and KofamKoala (default parameters). GenBank accession numbers for reference genomes of Syntropharchaeum, ANME-2 and ethane-oxidizers are (from left to right): GCA_001766815.1, SAMN09638579, GCA_003601555.1, [SRX359142](https://www.ncbi.nlm.nih.gov/sra/SRX359142%5baccn%5d), GCA_002503595.1, GCA_002506015.1, GCA_003194445.1, GCA_003601535.1, GCA_003661105.1, GCA_013572355.1, GCA_004211975.1, GCA_012961645.1, GCA_002926195.1, GCA_013572335.1, GCA_014237145.1, GCA_004193545.1, GCA_004212095.1.
