# Supplementary material for: Phylogenetic and functional diverse ANME-1 thrive in Arctic hydrothermal vents
Source: FEMS Microbiol Ecol. 2022 Oct 3;98(11):fiac117. doi: 10.1093/femsec/fiac117 (PMC9576274; doi:10.1093/femsec/fiac117)
Supplement: fiac117_Supplemental_Files [file fiac117_supplemental_files.zip › Supp_data_Table_1.docx]

**Supplementary Table 1.**CARD-FISH Oligonucleotides used in this study.

| Probe | Formamide% | Target | Sequence (5’ – 3’) | Target Site^1^ | Ref. |
| --- | --- | --- | --- | --- | --- |
| ANME-1-350 | 40 | ANME-1 | AGTTTTCGCGCCTGATGC | 350–367 | Boetius *et al*., 2000 |
| ANME-2a-647 | 45 | ANME-2 | TCTTCCGGTCCCAAGCCT | 647–664 | Knittel *et al*., 2005 |
| ANME-2c-662 | 45 | ANME-2 | CCCTTGGCAGTCTGATTG | 622–639 | Knittel *et al*., 2005 |
| SEEP1a‐1441 | 45 | Seep-SRB1a | CCCCTTGCGGGTTGGTCC | 1441–1470 | Schreiber *et al*., 2010 |
| SEEP1a‐473 | 45 | Seep-SRB1a | TTCAGTGATACCGTCAGTATCCC | 473–495 | Schreiber *et al*., 2010 |
| Seep-SRB2-658 | 45 | SEEP-SRB2 | TCCACTTCCCTCTCCGGT | 658-675 | Kleindienst *et al*., 2012 |
| HotSeep-1-1465 | 20 | *Ca*. Desulfofervidus | CGCCGACCACACCTTGGG | 183-201 | Krukenberg *et al*., 2016 |
| Delta495a | 35 | Deltaproteobacteria | AGTTAGCCGGTGCTTCCT | 495-512 | Loy *et al*., 2002 |
| EUB I-III-338 | 35 | Bacteria | GCTGCCTCCCGTAGGAGT;  GCAGCCACCCGTAGGTGT;  GCTGCCACCCGTAGGTGT | 338-355 | Daims *et al*., 1999; Amann *et al*., 2000 |
| Arch915 | 35 | Archaea | GTGCTCCCCCGCCAATTCCT | 915–934 | Amann *et al*., 1990 |

^1^Position in *Escherichia coli* 16S
