# Supplementary material for: Phylogenetic and functional diverse ANME-1 thrive in Arctic hydrothermal vents
Source: FEMS Microbiol Ecol. 2022 Oct 3;98(11):fiac117. doi: 10.1093/femsec/fiac117 (PMC9576274; doi:10.1093/femsec/fiac117)
Supplement: fiac117_Supplemental_Files [file fiac117_supplemental_files.zip › Supp_data_Table_2.docx]

**Supplementary Table 2.** List of single-copy marker genes used for concatenated phylogeny (HMM profile Archaea_76 (Lee 2019).

RNA_pol_L_2

RNA_pol_N

RNA_pol_Rpb4

RNA_pol_Rpb6

Ribosom_S12_S23

Ribosomal_L1

Ribosomal_L13

Ribosomal_L11

Ribosomal_L14

Ribosomal_L15e

Ribosomal_L16

Ribosomal_L21e

Ribosomal_L22

Ribosomal_L23

Ribosomal_L29

Ribosomal_L3

Ribosomal_L5e

Ribosomal_L6

Ribosomal_S11

Ribosomal_S13

Ribosomal_S15

Ribosomal_S17

Ribosomal_S17e

Ribosomal_S19

Ribosomal_S2

Ribosomal_S3Ae

Ribosomal_S7

Ribosomal_S8

Ribosomal_S9

Ribosomal_L4

RNase_HII

tRNA-synt_1c

tRNA-synt_1d

Ham1p_like

TIM
