# Supplementary material for: Phylogenetic and functional diverse ANME-1 thrive in Arctic hydrothermal vents
Source: FEMS Microbiol Ecol. 2022 Oct 3;98(11):fiac117. doi: 10.1093/femsec/fiac117 (PMC9576274; doi:10.1093/femsec/fiac117)
Supplement: fiac117_Supplemental_Files [file fiac117_supplemental_files.zip › Supp_data_Table_3_AB.docx]

**Supplementary Table 3A.**NCBI accession numbers of 16S rRNA sequences used in 16S rRNA phylogeny.

| AF134387.1 |
| --- |
| AF419624.1 |
| AF419625.1 |
| AF419626.1 |
| AF419630.1 |
| AF419631.1 |
| AF419632.1 |
| AF419655.1 |
| AF356644.1 |
| FR682489.1 |
| FR682490.1 |
| FR682491.1 |
| JF937715.1 |
| JF937719.1 |
| JF937746.1 |
| JF937751.1 |
| JF937755.1 |
| JF937770.1 |
| JF937791.1 |
| AF361687.1 |
| AJ578027.1 |
| AJ578140.1 |
| AF314249.1 |
| AY053468.1 |
| AY211702.1 |
| AY211727.1 |
| AJ578028.1 |
| AJ578029.1 |
| AJ578086.1 |
| AJ578110.1 |
| AJ578120.1 |
| AJ578123.1 |
| AJ578129.1 |
| AJ578130.1 |
| AJ578132.1 |
| AJ578133.1 |
| AJ578136.1 |
| AJ579316.1 |
| AY760632.1 |
| DQ270605.1 |
| AJ578096.1 |
| AF134393.1 |
| AF354130.1 |
| AF354134.1 |
| AF354143.1 |
| AJ578088.1 |
| AJ578090.1 |
| AJ578093.1 |
| AJ578097.1 |
| AJ578103.1 |
| AJ578107.1 |
| AJ578113.1 |
| AJ578116.1 |
| AJ578128.1 |
| AF354129.1 |
| AF354133.1 |
| AF354141.1 |
| AF354142.1 |
| AY053472.1 |
| AY053474.1 |
| AF419638.1 |
| AF419644.1 |
| AY211692.1 |
| AY211695.1 |
| AY211713.1 |
| AJ578083.1 |
| AJ578092.1 |
| AJ578095.1 |
| AJ578104.1 |
| AJ578112.1 |
| AJ578115.1 |
| AJ578118.1 |
| AF354136.1 |
| AY592077.1 |
| AY592286.1 |
| AJ704631.1 |
| AJ704650.1 |
| AJ704651.1 |
| AJ704652.1 |
| AJ704653.1 |
| AJ578119.1 |
| AF353127.1 |
| AF361688.1 |

| AY714860.1 |
| --- |

**Supplementary Table 3B.** GenBank accession numbers of genomes where 16S rRNA genes have been extracted for use in 16S rRNA phylogeny

| Organism name | GenBank accession |
| --- | --- |
| *Methanocalculu*s sp. 52_23 | GCA_001508455.1 |
| *Methanogenium cariaci* JCM 10550 (euryarchaeotes) | GCA_001315945.1 |
| *Methanosarcina barkeri* 3 (euryarchaeotes) | GCA_000970305.1 |
| *Methanosarcina mazei* (euryarchaeotes) | GCA_000978935.1 |
| *Methanosarcina mazei* (euryarchaeotes) | GCA_000979105.1 |
| *Methanosarcina mazei* (euryarchaeotes) | GCA_000978955.1 |
| *Methanosarcina siciliae* T4/M (euryarchaeotes) | GCA_000970085.1 |
| *Methanosarcina* sp. WWM596 (euryarchaeotes) | GCA_000969965.1 |
| *Methanosarcina* sp. WH1 (euryarchaeotes) | GCA_000970005.1 |
| WYZ_LMO13 | GCA_003601795.1 |
| G60ANME1 | GCA_003194435.1 |
| B48_G6 | GCA_003661165.1 |
| GoMg1 | GCA_013180605.1 |
| M5_MMPM | GCA_003160755.1 |
| CONS3730F07p2b1 | GCA_013374555.1 |
| CONS3730MDAH03UFb1 | GCA_013374565.1 |
| GoMg2 | GCA_013180585.1 |
| AG_394_G06 | GCA_009903405.1 |
| CR_Bin_179 | GCA_017883965.1 |
| SpSt_1198 | GCA_011049045.1 |
| CONS3730B06UFb1 | GCA_003336485.1 |
| GoMg4 | GCA_012979255.1 |
| GoMg3.2 | GCA_013180565.1 |
| GLR107 | GCA_013139985.1 |
