# Supplementary material for: Phylogenetic and functional diverse ANME-1 thrive in Arctic hydrothermal vents
Source: FEMS Microbiol Ecol. 2022 Oct 3;98(11):fiac117. doi: 10.1093/femsec/fiac117 (PMC9576274; doi:10.1093/femsec/fiac117)
Supplement: fiac117_Supplemental_Files [file fiac117_supplemental_files.zip › Supp_data_Table_4ABC_Sept2022.pdf]

Supplementary Table 4A. NCBI Biosample accession numbers

Biosample	Sample Name	Organism	Tax ID	NCBI isolate name
SAMN25610650	ANME-1-Barite_M4_B1	Methanosarcinales archaeon	2250255	Barite_M4_B1-AMOR_1a
SAMN25610651	ANME-1-Barite_M3_B26	Methanosarcinales archaeon	2250255	Barite_M3_B26-AMOR_1a
SAMN25610652	ANME-1-Barite_M2_B22	Methanosarcinales archaeon	2250255	Barite_M2_B22-AMOR_1a
SAMN25610653	ANME-1-Barite_M1_B61	Methanosarcinales archaeon	2250255	Barite_M1_B61-AMOR_1a
SAMN25610654	ANME-1-Flange_M5_B8	Methanosarcinales archaeon	2250255	Flange_M5_B8-AMOR_1b
SAMN25610655	ANME-1-INS_M14_B50	Methanosarcinales archaeon	2250255	INS_M14_B50-AMOR_1b
SAMN25610656	ANME-1-Barite_M1_B78	Methanosarcinales archaeon	2250255	Barite_M1_B78-AMOR_2
SAMN25610657	ANME-1-Barite_M4_B48	Methanosarcinales archaeon	2250255	Barite_M4_B48-AMOR_3
SAMN25610658	ANME-1-Barite_M2_B26	Methanosarcinales archaeon	2250255	Barite_M2_B26-AMOR_3
SAMN25610659	ANME-1-Barite_M4_B44	Methanosarcinales archaeon	2250255	Barite_M4_B44-AMOR_4
SAMN25610660	ANME-1-Barite_M2_B29	Methanosarcinales archaeon	2250255	Barite_M2_B29-AMOR_4
SAMN25610661	ANME-1-Barite_M1_B69	Methanosarcinales archaeon	2250255	Barite_M1_B69-AMOR_4
SAMN25610662	ANME-1-INS_M10_B59	Methanosarcinales archaeon	2250255	INS_M10_B59-AMOR_5b
SAMN25610663	ANME-1-INS_M11_B44	Methanosarcinales archaeon	2250255	INS_M11_B44-AMOR_5b
SAMN25610664	ANME-1-INS_M12_B72	Methanosarcinales archaeon	2250255	INS_M12_B72-AMOR_5b
SAMN25610665	ANME-1-INS_M14_B57	Methanosarcinales archaeon	2250255	INS_M14_B57-AMOR_5b
SAMN25610666	ANME-1-Flange_M5_B38	Methanosarcinales archaeon	2250255	Flange_M5_B38-AMOR_7
SAMN25716587	ANME-1_Bin00366_17ROV19_HD24_HD25	Methanosarcinales archaeon	2250255	LokisCastle_Sequencing2019_Bin00366
SAMN25716588	ANME-1_CaVeteromethanophagaceae_MAG00329_17ROV19_HD24_HD25	Methanosarcinales archaeon	2250255	LokisCastle_CaVeteromethanophagaceae_Sequencing2019_MAG00329

Supplementary Table 4B. Genome statistics of reconstructed MAGs from AMOR

Final MAG name	# ambiguous bases	# scaffolds	# contigs	N50 (scaffolds)	N50 (contigs)	Mean scaffold length (bp)	Mean contig length (bp)	Longest scaffold (bp)	Longest contig (bp)	GC std (scaffolds > 1kbp)	Coding density	Translation table
Barite_M1_B61	22923	214	339	8555	6334	7205	4480	28805	21669	1.86	86.1	11
Barite_M1_B69	2387	142	162	8102	7304	6914	6045	26030	26030	1.27	92.31	11
Barite_M1_B78	13022	171	268	10177	7477	8023	5070	30107	30107	1.63	84.24	11
Barite_M2_B29	5842	129	182	10093	9127	8715	6145	27586	24783	1.22	90.24	11
Barite_M2_B26	7263	83	137	18161	13859	15520	9349	56557	40755	1.06	91.34	11
Barite_M2_B22	14500	187	304	9725	7079	8122	4948	40601	36745	1.67	88.66	11
Barite_M3_B26	2926	191	230	11231	9842	8745	7249	36354	34406	1.66	90.7	11
Barite_M4_B1	27552	360	502	7013	5505	4997	3529	28955	26565	2.64	86.0	11
Barite_M4_B44	9552	116	169	12490	10681	10128	6895	38184	30428	1.26	89.34	11
Barite_M4_B48	13661	69	139	22669	15306	17655	8666	56142	51118	1.17	88.88	11
INS_M10_B59	2736	59	81	30636	24569	24045	17480	75502	60210	0.98	90.35	11
INS_M11_B44	5015	66	99	30392	23428	21673	14398	69496	52429	1.23	90.35	11
INS_M12_B72	4298	59	96	30070	24427	23768	14562	71856	59935	0.74	90.26	11
INS_M14_B50	4012	186	227	9368	8405	8105	6624	47580	41630	1.75	90.13	11
INS_M14_B57	8360	57	105	29999	21616	24221	13069	74429	56098	0.87	89.13	11
Flange_M5_B38	51	157	158	4314	4314	4183	4156	10633	10633	1.54	90.65	11
Flange_M5_B8	9372	220	301	11707	8858	8848	6436	58392	50517	1.87	88.35	11
Chimney19_Bin_00366	0	356	356	13779	13779	8720	8720	87222	87222	4.83	89.45	11
Chimney19_MAG_00329	0	245	245	7447	7447	6237	6237	22872	22872	1.94	84.66	11

Supplementary Table 4C. Names, geographic location, metadata, GCA and BioSample accesion number of MAGs from this study and references; information were collected from NCBI Database and published litterature. The GTDB-tk classification and closest taxonomical representative are also listed.

Isolate name	GenBank assembly accession	completeness (%)	contamination (%)	strain heterogeneity (%)	GC_content	Genome size (bp)	BioSample NCBI	geographic location NCBI	local/broad-scale environmental context
M5.MMPM	GCA_003160755.1	81.24	2.97	66.67	43.30	1,898,925	SAMN09045419	Aarhus Bay Station M5	marine sediment
B64_G16	GCA_003661125.1	83.83	3.27	0.00	42.99	2,212,588	SAMN09214940	Guaymas Basin. Gulf of California	hydrothermal sediments
WYZ-LMO12	GCA_003601545.1	73.94	9.23	4.76	42.27	2,163,043	SAMN08103064	Guaymas Basin. Gulf of California	oily sediments covered with mat
WYZ-LMO13	GCA_003601795.1	80.50	4.65	0.00	42.97	1,934,883	SAMN08103065	Guaymas Basin. Gulf of California	oily sediments covered with mat
GoMg1	GCA_013180605.1	80.80	2.94	16.67	42.66	2,344,285	SAMN08574220	Atlantic Ocean:Gulf of Mexico	cold seep
G37ANME1	GCA_003194425.1	87.91	1.31	50.00	52.19	1,398,999	SAMN08434975	Guaymas Basin. Gulf of California	hydrothermal sediments (AOM enrichment)
ex4572_4	GCA_002254785.1	69.91	1.96	33.33	53.22	1,018,085	SAMN06647984	Guaymas Basin. Gulf of California	hydrothermal sediments
HyVt-198	GCA_011041875.1	76.47	0.00	0.00	53.64	933.646	SAMN09638370	Guaymas Basin. Gulf of California	hydrothermal sediments
1244-F3-H4-B6	GCA_009618475.1	59.84	0.65	0.00	43.24	1,093,430	SAMN13181027	Pacific Ocean: Eastern North Pacific. ODP site 1244	methane clathrate
CR_Bin_179	GCA_017883965.1	90.31	2.61	20.00	41.50	2,478,969	SAMN18059653	Pacific Ocean: off Costa Rica Margin	coastal mud
ANME-1-THS	GCA_004212135.1	78.40	4.90	33.33	48.73	2,030,246	SAMN10387997	Tibet	Tibetan Hot Spring sediment
Kmv05	GCA_014859735.1	87.99	1.96	0.00	46.27	1,679,196	SAMN15871821	eastern Crimea	bubbling pool in crater-like structure of active mud volcano
SpSt-1198	GCA_011049045.1	93.14	3.46	14.29	55.24	2,003,787	SAMN09639115	California	hot spring sediment
GoMg4	GCA_012979255.1	51.26	0.87	50.00	44.04	1,341,504	SAMN08574218	Atlantic Ocean:Gulf of Mexico	cold seep
ANME-1-LC	GCA_014061035.1	87.99	1.96	40.00	44.52	1,073,849	SAMN15518188	Atlantic Ocean: Lost City Hydrothermal Field	hydrothermal field fluid
CONS3730B06UFb1	GCA_003336485.1	91.01	3.27	0.00	42.89	3,182,512	SAMN08574241	Pacific Ocean:Hydrate Ridge methane seep. Oregon	cold seep
AG-394-G06	GCA_009903405.1	63.48	0.65	0.00	42.76	1,531,297	SAMN08432499	Atlantic Ocean:Gulf of Mexico	cold seep
AG-394-G21	GCA_009903435.1	65.42	0.98	0.00	42.98	1,868,174	SAMN08432500	Atlantic Ocean:Gulf of Mexico	cold seep
UWMA-0191	GCA_012962515.1	62.71	3.92	33.33	43.37	1,401,145	SAMN10967532	Atlantic Ocean:Mid Cayman Rise	vent fluid
GoMg3.2	GCA_013180565.1	80.59	4.93	60.00	42.03	2,727,246	SAMN08574217	Atlantic Ocean:Gulf of Mexico	cold seep
GLR107	GCA_013139985.1	83.22	2.61	50.00	42.88	2,125,186	SAMN13287923	Hikurangi Margin	cold seep
GoMg2	GCA_013180585.1	79.21	3.92	37.50	42.67	2,192,919	SAMN08574219	Atlantic Ocean:Gulf of Mexico	cold seep
Agg-C03	GCA_013374425.1	63.37	3.27	20.00	43.59	1,895,081	SAMN08574226	Pacific Ocean:Hydrate Ridge methane seep. Oregon	cold seep
CONS3730H04p2b1	GCA_013374505.1	76.47	1.63	50.00	43.28	2,571,212	SAMN08574227	Pacific Ocean:Hydrate Ridge methane seep. Oregon	cold seep
CONS3730F07p2b1	GCA_013374555.1	74.44	6.05	10.00	43.13	2,752,395	SAMN08574224	Pacific Ocean:Hydrate Ridge methane seep. Oregon	cold seep
CONS3730MDAH03UFb1	GCA_013374565.1	82.14	18.16	79.49	43.28	3,408,029	SAMN08574223	Pacific Ocean:Hydrate Ridge methane seep. Oregon	cold seep
G60ANME1	GCA_003194435.1	88.24	1.96	66.67	45.84	1,805,851	SAMN08434988	Guaymas Basin. Gulf of California	hydrothermal sediments (AOM enrichment)
B48_G6	GCA_003661165.1	75.50	1.96	50.00	45.15	1,204,084	SAMN09215141	Guaymas Basin. Gulf of California	hydrothermal sediments (AOM enrichment)
B22_G9	GCA_003661195.1	69.23	1.63	66.67	46.76	1,032,966	SAMN09215218	Guaymas Basin. Gulf of California	hydrothermal sediments
B39_G2	GCA_003661185.1	83.33	1.31	0.00	51.89	1,690,640	SAMN09215057	Guaymas Basin. Gulf of California	hydrothermal sediments
HyVt-11	GCA_011043905.1	61.28	0.84	40.00	54.45	1,254,427	SAMN09638273	Guaymas Basin. Gulf of California	hydrothermal sediment
Barite_M1_B61		65.48	5.45	0.00	42.7	1,541,944	(see Supp. Table 4A)	AMOR	barite chimney (LCBF chimney - BaCh2W) (see Table 1)
Barite_M1_B69		73.04	1.36	0.00	48.6	981.829	(see Supp. Table 4A)	AMOR	barite chimney (LCBF chimney - BaCh2W) (see Table 1)
Barite_M1_B78		74.35	6.54	0.00	44.3	1,372,011	(see Supp. Table 4A)	AMOR	barite chimney (LCBF chimney - BaCh2W) (see Table 1)
Barite_M2_B22		74.84	1.31	0.00	43.1	1,518,868	(see Supp. Table 4A)	AMOR	barite chimney (LCBF chimney - BaCh4M) (see Table 1)
Barite_M2_B26		88.24	0.65	0.00	50.7	1,288,204	(see Supp. Table 4A)	AMOR	barite chimney (LCBF chimney - BaCh4M) (see Table 1)
Barite_M2_B29		84.35	2.31	60.00	49.3	1,124,287	(see Supp. Table 4A)	AMOR	barite chimney (LCBF chimney - BaCh4M) (see Table 1)
Barite_M3_B26		81.65	1.96	0.00	43.2	1,670,303	(see Supp. Table 4A)	AMOR	barite chimney (LCBF chimney - BaCh3G) (see Table 1)
Barite_M4_B1		75.82	1.96	33.33	43.4	1,799,200	(see Supp. Table 4A)	AMOR	hydrothermal sediments (LCBF sediments) (see Table 1)
Barite_M4_B44		79.01	2.61	25.00	49.2	1,174,921	(see Supp. Table 4A)	AMOR	hydrothermal sediments (LCBF sediments) (see Table 1)
Barite_M4_B48		86.74	0.65	0.00	50.7	1,218,258	(see Supp. Table 4A)	AMOR	hydrothermal sediments (LCBF sediments) (see Table 1)
INS_M10_B59		90.52	0.65	0.00	47.9	1,418,687	(see Supp. Table 4A)	AMOR	hydrothermal sediments (JMVF sediments) (see Table 1)
INS_M11_B44		90.31	0.65	0.00	47.9	1,430,433	(see Supp. Table 4A)	AMOR	hydrothermal sediments (JMVF sediments) (see Table 1)
INS_M12_B72		89.54	0.65	0.00	48.0	1,402,330	(see Supp. Table 4A)	AMOR	hydrothermal sediments (JMVF sediments) (see Table 1)
INS_M14_B50		76.29	2.29	0.00	43.3	1,507,692	(see Supp. Table 4A)	AMOR	hydrothermal sediments (JMVF sediments) (see Table 1)
INS_M14_B57		88.34	0.65	0.00	47.9	1,380,621	(see Supp. Table 4A)	AMOR	hydrothermal sediments (JMVF sediments) (see Table 1)
Flange_M5_B38		51.41	1.31	0.00	46.7	656.734	(see Supp. Table 4A)	AMOR	black smoker flange (JMVF white smoker flange) (see Table 1)
Flange_M5_B8		84.10	4.25	57.14	43.3	1,946,644	(see Supp. Table 4A)	AMOR	black smoker flange (JMVF white smoker flange) (see Table 1)
Chimney19_Bin_00366		88.56	3.92	0.00	42.2	3,104,503	(see Supp. Table 4A)	AMOR	black smoker flange (LCVF black smoker) (see Table 1)
Chimney19_MAG_00329		75.34	2.36	85.71	46.5	1,528,068	(see Supp. Table 4A)	AMOR	black smoker flange (LCVF black smoker) (see Table 1)

(continues from Supplementary Table 4C)

Isolate name	Reference	classification (GTDB-tk)	closest_placement_reference	closest_placement_ani
M5.MMPM	Wang Y et al.. 2019	d__Archaea;p__Halobacteriota;c__Syntropharchaeia;o__ANME-1;f__ANME-1;g__QEXZ01;s__QEXZ01 sp003601795	GCA_003601795.1	100
B64_G16	Yu H et al.. 2018	d__Archaea;p__Halobacteriota;c__Syntropharchaeia;o__ANME-1;f__ANME-1;g__QEXZ01;s__QEXZ01 sp003661125	GCA_003661125.1	100
WYZ-LMO12	Seitz et al.. 2019	d__Archaea;p__Halobacteriota;c__Syntropharchaeia;o__ANME-1;f__ANME-1;g__QEXZ01;s__	GCA_003661125.1	89.93
WYZ-LMO13	Wang Y et al.. 2019	d__Archaea;p__Halobacteriota;c__Syntropharchaeia;o__ANME-1;f__ANME-1;g__QEXZ01;s__QEXZ01 sp003601795	GCA_003601795.1	100
GoMg1	Yu H et al.. 2018	d__Archaea;p__Halobacteriota;c__Syntropharchaeia;o__ANME-1;f__ANME-1;g__QEXZ01;s__QEXZ01 sp013180605	GCA_013180605.1	100
G37ANME1	Krukenberg et al.. 2018	d__Archaea;p__Halobacteriota;c__Syntropharchaeia;o__ANME-1;f__ANME-1;g__ANME1a;s__ANME1a sp003194425	GCA_003194425.1	98.99
ex4572_4	Seitz et al.. 2019	d__Archaea; p__Halobacterota; c__Syntrophoarchaeia; o__ANME-1; f__ANME-1; g__ANME1a; s__ANME1a sp003194425	N/A	N/A
HyVt-198	Zhou et al.. 2020	d__Archaea;p__Halobacteriota;c__Syntropharchaeia;o__ANME-1;f__ANME-1;g__ANME1a;s__ANME1a sp003194425	GCA_003194425.1	98.99
1244-F3-H4-B6	\	d__Archaea;p__Halobacteriota;c__Syntropharchaeia;o__ANME-1;f__ANME-1;g__;s__	N/A	N/A
CR_Bin_179	\	d__Archaea;p__Halobacteriota;c__Syntropharchaeia;o__ANME-1;f__ANME-1;g__WJOV01;s__	GCA_009618475.1	83.63
ANME-1-THS	Borrel et al.. 2019	d__Archaea;p__Halobacteriota;c__Syntropharchaeia;o__ANME-1;f__ANME-1;g__ANME-1-THS;s__ANME-1-THS sp004212135	GCA_004212135.1	100
Kmv05	\	d__Archaea;p__Halobacteriota;c__Syntropharchaeia;o__ANME-1;f__ANME-1;g__ANME-1-THS;s__	GCA_004212135.1	79.68
SpSt-1198	Zhou et al.. 2020	d__Archaea;p__Halobacteriota;c__Syntropharchaeia;o__ANME-1;f__ANME-1;g__ANME-1-THS;s__	GCA_004212135.1	76.67
GoMg4	Yu H et al.. 2018	d__Archaea;p__Halobacteriota;c__Syntropharchaeia;o__ANME-1;f__ANME-1;g__ANME-1-THS;s__	GCA_004212135.1	76.25
ANME-1-LC	Yu H et al.. 2018	d__Archaea;p__Halobacteriota;c__Syntropharchaeia;o__ANME-1;f__ANME-1;g__JACGMN01;s__JACGMN01 sp014061035	GCA_014061035.1	100
CONS3730B06UFb1	Yu H et al.. 2018	d__Archaea;p__Halobacteriota;c__Syntropharchaeia;o__ANME-1;f__ANME-1;g__QENH01;s__QENH01 sp003336485	GCA_003336485.1	100
AG-394-G06	Yu H et al.. 2018	d__Archaea;p__Halobacteriota;c__Syntropharchaeia;o__ANME-1;f__ANME-1;g__QENH01;s__	GCA_003336485.1	88.62
AG-394-G21	Yu H et al.. 2018	d__Archaea;p__Halobacteriota;c__Syntropharchaeia;o__ANME-1;f__ANME-1;g__QENH01;s__	GCA_003336485.1	88.73
UWMA-0191	\	d__Archaea;p__Halobacteriota;c__Syntropharchaeia;o__ANME-1;f__ANME-1;g__QENH01;s__	N/A	N/A
GoMg3.2	Yu H et al.. 2018	d__Archaea;p__Halobacteriota;c__Syntropharchaeia;o__ANME-1;f__ANME-1;g__QENH01;s__QENH01 sp013180565	GCA_013180565.1	100
GLR107	Yu H et al.. 2018	d__Archaea;p__Halobacteriota;c__Syntropharchaeia;o__ANME-1;f__ANME-1;g__QENH01;s__QENH01 sp013139985	GCA_013139985.1	100
GoMg2	Yu H et al.. 2018	d__Archaea;p__Halobacteriota;c__Syntropharchaeia;o__ANME-1;f__ANME-1;g__QENJ01;s__QENJ01 sp013180585	GCA_013180585.1	100
Agg-C03	Yu H et al.. 2018	d__Archaea;p__Halobacteriota;c__Syntropharchaeia;o__ANME-1;f__ANME-1;g__QENJ01;s__	GCA_013374505.1	91.45
CONS3730H04p2b1	Yu H et al.. 2018	d__Archaea;p__Halobacteriota;c__Syntropharchaeia;o__ANME-1;f__ANME-1;g__QENJ01;s__QENJ01 sp013374505	GCA_013374505.1	100
CONS3730F07p2b1	Yu H et al.. 2018	d__Archaea;p__Halobacteriota;c__Syntropharchaeia;o__ANME-1;f__ANME-1;g__QENJ01;s__	GCA_013374505.1	91.89
CONS3730MDAH03UFb1	Yu H et al.. 2018	d__Archaea;p__Halobacteriota;c__Syntropharchaeia;o__ANME-1;f__ANME-1;g__QENJ01;s__	GCA_013374505.1	91.67
G60ANME1	Krukenberg et al.. 2018	d__Archaea;p__Halobacteriota;c__Syntropharchaeia;o__ANME-1;f__ANME-1;g__G60ANME1;s__	GCA_003194435.1	91.63
B48_G6	Seitz et al.. 2019	d__Archaea;p__Halobacteriota;c__Syntropharchaeia;o__ANME-1;f__ANME-1;g__G60ANME1;s__	GCA_003194435.1	91.63
B22_G9	Seitz et al.. 2019	d__Archaea;p__Halobacteriota;c__Syntropharchaeia;o__ANME-1;f__;g__;s__	N/A	N/A
B39_G2	Seitz et al.. 2019	d__Archaea;p__Halobacteriota;c__Syntropharchaeia;o__ANME-1;f__B39-G2;g__B39-G2;s__B39-G2 sp003661185	GCA_003661185.1	100
HyVt-11	Zhou et al.. 2020	d__Archaea;p__Halobacteriota;c__Syntropharchaeia;o__ANME-1;f__B39-G2;g__B39-G2;s__	GCA_003661185.1	80.45
Barite_M1_B61	this study	d__Archaea;p__Halobacteriota;c__Syntropharchaeia;o__ANME-1;f__ANME-1;g__QEXZ01;s__	GCA_003661125.1	84.05
Barite_M1_B69	this study	d__Archaea;p__Halobacteriota;c__Syntropharchaeia;o__ANME-1;f__ANME-1;g__G60ANME1;s__	GCA_003194435.1	77.89
Barite_M1_B78	this study	d__Archaea;p__Halobacteriota;c__Syntropharchaeia;o__ANME-1;f__ANME-1;g__QEXZ01;s__	N/A	N/A
Barite_M2_B22	this study	d__Archaea;p__Halobacteriota;c__Syntropharchaeia;o__ANME-1;f__ANME-1;g__QEXZ01;s__	GCA_003661125.1	84.4
Barite_M2_B26	this study	d__Archaea;p__Halobacteriota;c__Syntropharchaeia;o__ANME-1;f__ANME-1;g__G60ANME1;s__	GCA_003194435.1	79.63
Barite_M2_B29	this study	d__Archaea;p__Halobacteriota;c__Syntropharchaeia;o__ANME-1;f__ANME-1;g__G60ANME1;s__	GCA_003194435.1	77.38
Barite_M3_B26	this study	d__Archaea;p__Halobacteriota;c__Syntropharchaeia;o__ANME-1;f__ANME-1;g__QEXZ01;s__	GCA_003661125.1	84.69
Barite_M4_B1	this study	d__Archaea;p__Halobacteriota;c__Syntropharchaeia;o__ANME-1;f__ANME-1;g__QEXZ01;s__	GCA_003661125.1	84.85
Barite_M4_B44	this study	d__Archaea;p__Halobacteriota;c__Syntropharchaeia;o__ANME-1;f__ANME-1;g__G60ANME1;s__	GCA_003194435.1	77.29
Barite_M4_B48	this study	d__Archaea;p__Halobacteriota;c__Syntropharchaeia;o__ANME-1;f__ANME-1;g__G60ANME1;s__	GCA_003194435.1	79.52
INS_M10_B59	this study	d__Archaea;p__Halobacteriota;c__Syntropharchaeia;o__ANME-1;f__ANME-1;g__G60ANME1;s__	GCA_003194435.1	77.72
INS_M11_B44	this study	d__Archaea;p__Halobacteriota;c__Syntropharchaeia;o__ANME-1;f__ANME-1;g__G60ANME1;s__	GCA_003194435.1	77.77
INS_M12_B72	this study	d__Archaea;p__Halobacteriota;c__Syntropharchaeia;o__ANME-1;f__ANME-1;g__G60ANME1;s__	GCA_003194435.1	77.81
INS_M14_B50	this study	d__Archaea;p__Halobacteriota;c__Syntropharchaeia;o__ANME-1;f__ANME-1;g__QEXZ01;s__	GCA_003661125.1	83.74
INS_M14_B57	this study	d__Archaea;p__Halobacteriota;c__Syntropharchaeia;o__ANME-1;f__ANME-1;g__G60ANME1;s__	GCA_003194435.1	77.77
Flange_M5_B38	this study	d__Archaea;p__Halobacteriota;c__Syntropharchaeia;o__ANME-1;f__;g__;s__	N/A	
Flange_M5_B8	this study	d__Archaea;p__Halobacteriota;c__Syntropharchaeia;o__ANME-1;f__ANME-1;g__QEXZ01;s__	GCA_003661125.1	84.15
Chimney19_Bin_00366	this study	d__Archaea;p__Halobacteriota;c__Syntropharchaeia;o__ANME-1;f__ANME-1;g__G60ANME1;s__	GCA_003194435.1	77.77
Chimney19_MAG_00329	this study	d__Archaea;p__Halobacteriota;c__Syntropharchaeia;o__ANME-1;f__;g__;s__	N/A	N/A
