# Supplementary material for: Phylogenetic and functional diverse ANME-1 thrive in Arctic hydrothermal vents
Source: FEMS Microbiol Ecol. 2022 Oct 3;98(11):fiac117. doi: 10.1093/femsec/fiac117 (PMC9576274; doi:10.1093/femsec/fiac117)
Supplement: fiac117_Supplemental_Files [file fiac117_supplemental_files.zip › Supp_data_Table_5.docx]

**Supplementary Table 5.**Summary of AMOR ANME-1 (AA), AMOR Veteromethanophagaceae (AVet) and AMOR Alkanophagaceae (AAlk) subgroups and affiliated MAGs. ‘LCBF’: Loki’s Castle Barite Field, ‘JMVF’: Jan Mayen Vent Field, ‘LCVF’: Loki’s Castle Vent Field.

| **AMOR subgroups (<95% ANI)** | **MAGs ID** | **Sample ID** |
| --- | --- | --- |
| AA_1 | Barite_M4_B1 | LCBF sediments |
|  | Barite_M3_B26 | LCBF chimney |
|  | Barite_M2_B22 | LCBF chimney |
|  | Barite_M1_B61 | LCBF chimney |
|  | Flange_M5_B8 | JMVF white smoker flange |
|  | INS_M14_B50 | JMVF sediments |
| AA_2 | Barite_M1_B78 | LCBF chimney |
| AA_3 | Barite_M4_B48 | LCBF sediments |
|  | Barite_M2_B26 | LCBF chimney |
| AA_4 | Barite_M4_B44 | LCBF sediments |
|  | Barite_M2_B29 | LCBF chimney |
| AA_5 | Barite_M1_B69 | LCBF chimney |
| AA_6 | Chimney19_Bin_00366 | LCVF black smoker |
|  | INS_M10_B59 | JMVF sediments |
|  | INS_M11_B44 | JMVF sediments |
|  | INS_M12_B72 | JMVF sediments |
|  | INS_M14_B57 | JMVF sediments |
| AVet_7 | Chimney19_MAG_00329 | LCVF black smoker |
| AAlk_8 | Flange_M5_B38 | JMVF white smoker flange |
