# Supplementary material for: Phylogenetic and functional diverse ANME-1 thrive in Arctic hydrothermal vents
Source: FEMS Microbiol Ecol. 2022 Oct 3;98(11):fiac117. doi: 10.1093/femsec/fiac117 (PMC9576274; doi:10.1093/femsec/fiac117)
Supplement: fiac117_Supplemental_Files [file fiac117_supplemental_files.zip › Supp_data_Table_6AB.pdf]

Supplementary Table 6A: list of KO numbers used for MAGs functional analysis. Information about names and ID numbers were collected from KEGG Database at https://www.genome.jp/kegg/ (Kanehisa et al., 2000)

Predicted protein name	Gene name	KO number	EC number
methyl-CoM reductase (Mcr) alpha subunit	mcrA	K00399	EC:2.8.4.1
methyl coenzyme M reductase system, component A2	K00400	K00400	
methyl-coenzyme M reductase beta subunit	mcrB	K00401	EC:2.8.4.1
methyl-coenzyme M reductase gamma subunit	mcrG	K00402	EC:2.8.4.1
methyl-coenzyme M reductase subunit C	mcrC	K03421	
methyl-coenzyme M reductase subunit D	mcrD	K03422	
Methyl-H4M(S)PT:HS-CoM methyltransferase(Mtr) subunitA	mtrA	K00577	EC:2.1.1.86
tetrahydromethanopterin S-methyltransferase subunit B	mtrB	K00578	EC:2.1.1.86
tetrahydromethanopterin S-methyltransferase subunit C	mtrC	K00579	EC:2.1.1.86
tetrahydromethanopterin S-methyltransferase subunit D	mtrD	K00580	EC:2.1.1.86
tetrahydromethanopterin S-methyltransferase subunit E	mtrE	K00581	EC:2.1.1.86
tetrahydromethanopterin S-methyltransferase subunit F	mtrF	K00582	EC:2.1.1.86
tetrahydromethanopterin S-methyltransferase subunit G	mtrG	K00583	EC:2.1.1.86
tetrahydromethanopterin S-methyltransferase subunit H	mtrH	K00584	EC:2.1.1.86
Methylene-H4M(S)PT reductase(Mer)	mer	K00320	EC:1.5.98.2 (1.5.99.11)
bifunctional enzyme fae/hps	fae-hps	K13812	EC:4.2.1.147 4.1.2.43
methylenetetrahydrofolate reductase (NADPH)	metF, MTHFR	K00297	EC:1.5.1.20
Methylene-H4M(S)PT dehydrogenase(Hmd)	mtd	K00319	EC:1.5.98.1 (1.5.99.9)
Methenye-H4M(S)PT cyclohydrolase(Mch)	mch	K01499	EC:3.5.4.27
Formyl-MFR:H4M(S)PT formyltransferase(Ftr)	ftr	K00672	EC:2.3.1.101
Formyl-MFR dehydrogenase(Fmd) subunit A	fwdA, fmdA	K00200	EC:1.2.99.5 (EC:1.2.7.12)
Formyl-MFR dehydrogenase(Fmd) subunit B	fwdB, fmdB	K00201	EC:1.2.99.5 (EC:1.2.7.12)
Formyl-MFR dehydrogenase(Fmd) subunit C	fwdC, fmdC	K00202	EC:1.2.99.5 (EC:1.2.7.12)
Formyl-MFR dehydrogenase(Fmd) subunit D	fwdD, fmdD	K00203	EC:1.2.99.5 (EC:1.2.7.12)
formylmethanofuran dehydrogenase subunit E	fwdE, fmdE	K11261	EC:1.2.99.5 (EC:1.2.7.12)
4Fe-4S ferredoxin	fwdF, fmdF	K00205	
4Fe-4S ferredoxin	fwdG	K11260	
4Fe-4S ferredoxin	fwdH	K00204	
[methyl-Co(III) methanol-specific corrinoid protein]:coenzyme M methyltransferase	mtaA	K14080	EC:2.1.1.246
methanol---5-hydroxybenzimidazolylcobamide Co-methyltransferase	mtaB	K04480	EC:2.1.1.90
methanol corrinoid protein	mtaC	K14081	
acetyl-CoA decarbonylase/synthase complex subunit beta	cdhC	K00193	EC:2.3.1.-
acetyl-CoA decarbonylase/synthase complex subunit delta	cdhD, acsD	K00194	EC:2.1.1.245
acetyl-CoA decarbonylase/synthase complex subunit gamma	cdhE, acsC	K00197	EC:2.1.1.245
acetyl-CoA decarbonylase/synthase complex subunit alpha	cdhA	K00192	EC:1.2.7.4
acetyl-CoA decarbonylase/synthase complex subunit epsilon	cdhB	K00195	
carbon-monoxide dehydrogenase catalytic subunit	cooS, acsA	K00198	EC:1.2.7.4
anaerobic carbon-monoxide dehydrogenase iron sulfur subunit	cooF	K00196	
acetyl-CoA synthetase	ACSS, acs	K01895	EC:6.2.1.1
putative acetyl-CoA synthetase, alpha subunit	acdA	K01905	EC:6.2.1.13
putative acetyl-CoA synthetase, beta subunit	acdB	K22224	EC:6.2.1.13
acetate---CoA ligase (ADP-forming)	acdAB	K24012	EC:6.2.1.13
heterodisulfide reductase subunit A1	hdrA1	K22480	EC:1.8.7.3
heterodisulfide reductase subunit B1	hdrB1	K22481	EC:1.8.7.3
heterodisulfide reductase subunit C1	hdrC1	K22482	EC:1.8.7.3
heterodisulfide reductase subunit A2	hdrA2	K03388	EC:1.8.7.3 1.8.98.4 1.8.98.5 1.8.98.6
heterodisulfide reductase subunit B2	hdrB2	K03389	EC:1.8.7.3 1.8.98.4 1.8.98.5 1.8.98.6
heterodisulfide reductase subunit C2	hdrC2	K03390	EC:1.8.7.3 1.8.98.4 1.8.98.5 1.8.98.6
heterodisulfide reductase subunit D	hdrD	K08264	EC:1.8.98.1
heterodisulfide reductase subunit E	hdrE	K08265	EC:1.8.98.1
formate dehydrogenase (coenzyme F420) alpha subunit	fdhA	K22516	EC:1.17.98.3 1.8.98.6
formate dehydrogenase (coenzyme F420) beta subunit	fdhB	K00125	EC:1.17.98.3 1.8.98.6
V/A-type H+/Na+-transporting ATPase subunit A	ATPVA, ntpA, atpA	K02117	EC:3.6.3.14 3.6.3.15 (EC:7.1.2.2 7.2.2.1)
V/A-type H+/Na+-transporting ATPase subunit B	ATPVB, ntpB, atpB	K02118	EC:3.6.3.14 3.6.3.15 (EC:7.1.2.2 7.2.2.1)
V/A-type H+/Na+-transporting ATPase subunit C	ATPVC, ntpC, atpC	K02119	EC:3.6.3.14 3.6.3.15 (EC:7.1.2.2 7.2.2.1)
V/A-type H+/Na+-transporting ATPase subunit D	ATPVD, ntpD, atpD	K02120	EC:3.6.3.14 3.6.3.15 (EC:7.1.2.2 7.2.2.1)
V/A-type H+/Na+-transporting ATPase subunit E	ATPVE, ntpE, atpE	K02121	EC:3.6.3.14 3.6.3.15 (EC:7.1.2.2 7.2.2.1)
V/A-type H+/Na+-transporting ATPase subunit F	ATPVF, ntpF, atpF	K02122	EC:3.6.3.14 3.6.3.15 (EC:7.1.2.2 7.2.2.1)
V/A-type H+/Na+-transporting ATPase subunit I	ATPVI, ntpI, atpI	K02123	EC:3.6.3.14 3.6.3.15 (EC:7.1.2.2 7.2.2.1)
V/A-type H+/Na+-transporting ATPase subunit K	ATPVK, ntpK, atpK	K02124	EC:3.6.3.14 3.6.3.15 (EC:7.1.2.2 7.2.2.1)
ech hydrogenase subunit A	echA	K14086	
ech hydrogenase subunit B	echB	K14087	
ech hydrogenase subunit C	echC	K14088	
ech hydrogenase subunit D	echD	K14089	
ech hydrogenase subunit E	echE	K14090	
ech hydrogenase subunit F	echF	K14091	
methanophenazine hydrogenase, large subunit	vhoA, vhtA;	K14068	
methanophenazine hydrogenase, cytochrome b subunit	vhoC, vhtC;	K14069	
methanophenazine hydrogenase	vhoG, vhtG;	K14070	
F420H2 dehydrogenase subunit A	fpoA	K22158	EC:1.5.98.3
F420H2 dehydrogenase subunit B	fpoB	K22159	EC:1.5.98.3
F420H2 dehydrogenase subunit C	fpoC	K22160	EC:1.5.98.3
F420H2 dehydrogenase subunit D	fpoD	K22161	EC:1.5.98.3
F420H2 dehydrogenase subunit F	fpoF	K22162	EC:1.5.98.3
F420H2 dehydrogenase subunit H	fpoH	K22163	EC:1.5.98.3
F420H2 dehydrogenase subunit I	fpoI	K22164	EC:1.5.98.3
F420H2 dehydrogenase subunit J	fpoJ	K22165	EC:1.5.98.3
F420H2 dehydrogenase subunit K	fpoK	K22166	EC:1.5.98.3
F420H2 dehydrogenase subunit L	fpoL	K22167	EC:1.5.98.3
F420H2 dehydrogenase subunit M	fpoM	K22168	EC:1.5.98.3
F420H2 dehydrogenase subunit N	fpoN	K22169	EC:1.5.98.3
F420H2 dehydrogenase subunit O	fpoO	K22170	EC:1.5.98.3
F420H2:quinone oxidoreductase subunit A	fqoA (nuoA)	K22171	EC:1.1.98.4
F420H2:quinone oxidoreductase subunit B/C	fqoBC (nuoBC)	K22172	EC:1.1.98.4
F420H2:quinone oxidoreductase subunit D	fqoD	K22173	EC:1.1.98.4
F420H2:quinone oxidoreductase subunit F	fqoF	K22174	EC:1.1.98.4
F420H2:quinone oxidoreductase subunit H	fqoH (nuoH)	K22175	EC:1.1.98.4
F420H2:quinone oxidoreductase subunit I	fqoI (nuoI)	K22176	EC:1.1.98.4
F420H2:quinone oxidoreductase subunit J	fqoJ (nuoJ)	K22177	EC:1.1.98.4
F420H2:quinone oxidoreductase subunit K	fqoK	K22178	EC:1.1.98.4
F420H2:quinone oxidoreductase subunit L	fqoL	K22179	EC:1.1.98.4
F420H2:quinone oxidoreductase subunit M	fqoM	K22180	EC:1.1.98.4
F420H2:quinone oxidoreductase subunit N	fqoN	K22181	EC:1.1.98.4
NADH-quinone oxidoreductase subunit A	nuoA	K00330	EC:7.1.1.2 (EC:1.6.5.3)
NADH-quinone oxidoreductase subunit B	nuoB	K00331	EC:7.1.1.2 (EC:1.6.5.3)
NADH-quinone oxidoreductase subunit C/D	nuoCD	K13378	EC:7.1.1.2 (EC:1.6.5.3)
NADH-quinone oxidoreductase subunit D	nuoD	K00333	EC:7.1.1.2 (EC:1.6.5.3)
NADH-quinone oxidoreductase subunit G	nuoG	K00336	EC:7.1.1.2 (EC:1.6.5.3)
NADH-quinone oxidoreductase subunit H	nuoH	K00337	EC:7.1.1.2 (EC:1.6.5.3)
NADH-quinone oxidoreductase subunit I	nuoI	K00338	EC:7.1.1.2 (EC:1.6.5.3)
NADH-quinone oxidoreductase subunit L	nuoL	K00341	EC:7.1.1.2 (EC:1.6.5.3)

NADH-quinone oxidoreductase subunit M	nuoM	K00342	EC:7.1.1.2 (EC:1.6.5.3)
NADH-quinone oxidoreductase subunit N	nuoN	K00343	EC:7.1.1.2 (EC:1.6.5.3)
Coenzyme F420 hydrogenase subunit alpha	frhA	K00440	1.12.98.1
probable coenzyme F420-reducing hydrogenase, beta subunit	frhB	K00441	1.12.98.1
frhD; coenzyme F420 hydrogenase subunit delta	frhD	K00442	1.12.98.1
coenzyme F420 hydrogenase subunit gamm	frhG	K00443	1.12.98.1
F420-non-reducing hydrogenase large subunit	mvhA, vhuA, vhcA	K14126	EC:1.12.99.- 1.8.98.5
F420-non-reducing hydrogenase iron-sulfur subunit	mvhD, vhuD, vhcD	K14127	EC:1.12.99.- 1.8.98.6
F420-non-reducing hydrogenase small subunit	mvhG, vhuG, vhcG	K14128	EC:1.12.99.- 1.8.98.5
acetyl-CoA C-acetyltransferase	atoB	K00626	EC:2.3.1.9
3-hydroxybutyryl-CoA dehydrogenase	hbd	K00074	EC:1.1.1.157
enoyl-CoA hydratase	crt	K01715	EC:4.2.1.17
butyryl-CoA dehydrogenase	ACADS	K00248	EC:1.3.8.1
two-component system, chemotaxis family, sensor kinase CheA	cheA	K03407	EC:2.7.13.3
two-component system, chemotaxis family, CheB/CheR fusion protein	cheBR	K13924	EC:2.1.1.80 3.1.1.61
two-component system, chemotaxis family, protein-glutamate methylesterase/glutaminase	cheB	K03412	
chemotaxis protein CheC	cheC	K03410	
chemotaxis protein CheD	cheD	K03411	EC:3.5.1.44
taxis protein CheF	cheF	K09156	
two-component system, chemotaxis family, chemotaxis protein CheY	cheY	K03413	
chemotaxis protein methyltransferase CheR	cheR	K00575	EC:2.1.1.80
purine-binding chemotaxis protein CheW	CheW	K03408	
chemotaxis protein CheX	CheX	K03409	
chemotaxis protein CheZ	CheZ	K03414	
two-component system, chemotaxis family, chemotaxis protein CheV	CheV	K03415	
methyl-accepting chemotaxis protein	MCP	K03406	
archaeal flagellin FlaA	FlaA	K07324	
archaeal flagellin FlaB	FlaB	K07325	
archaeal flagellar protein FlaH	FlaH	K07331	
archaeal flagellar protein FlaI	FlaI	K07332	
archaeal flagellar protein FlaJ	FlaJ	K07333	
archaeal preflagellin peptidase FlaK	FlaK	K07991	EC:3.4.23.52
glyceraldehyde 3-phosphate dehydrogenase	GAPDH, gapA	K00134	EC:1.2.1.12
glyceraldehyde-3-phosphate dehydrogenase (NAD(P))	gap2	K00150	EC:1.2.1.59
pyruvate ferredoxin oxidoreductase alpha subunit	porA	K00169	EC:1.2.7.1
pyruvate ferredoxin oxidoreductase beta subunit	porB	K00170	EC:1.2.7.1
pyruvate ferredoxin oxidoreductase delta subunit	porD	K00171	EC:1.2.7.1
pyruvate ferredoxin oxidoreductase gamma subunit	porC, porG	K00172	EC:1.2.7.1
2-oxoisovalerate/pyruvate ferredoxin oxidoreductase gamma subunit	vorG, porG	K00189	EC:1.2.7.7 1.2.7.1
anaerobic carbon-monoxide dehydrogenase iron sulfur subunit	cooF	K00196	
anaerobic carbon-monoxide dehydrogenase catalytic subunit	cooS, acsA	K00198	EC:1.2.7.4
hexokinase	HK	K00844	EC:2.7.1.1
glucokinase	glk	K00845	EC:2.7.1.2
6-phosphofructokinase 1	pfkA, PFK	K00850	EC:2.7.1.11
polyphosphate glucokinase	ppgK	K00886	EC:2.7.1.63
diphosphate-dependent phosphofructokinase	pfp, PFP	K00895	EC:2.7.1.90
ADP-dependent phosphofructokinase/glucokinase	pfkC	K00918	EC:2.7.1.146 2.7.1.147
pyruvate, water dikinase	pps, ppsA	K01007	EC:2.7.9.2
glucose-6-phosphatase	G6PC	K01084	EC:3.1.3.9
fructose-1,6-bisphosphatase I / sedoheptulose-1,7-bisphosphatase	fbp-SEBP	K01086	EC:3.1.3.11 3.1.3.37
fructose 1,6-bisphosphate aldolase/phosphatase	K01622	K01622	EC:4.1.2.13 3.1.3.11
fructose-bisphosphate aldolase, class I	ALDO	K01623	EC:4.1.2.13
fructose-bisphosphate aldolase, class II	FBA, fbaA	K01624	EC:4.1.2.13
enolase	ENO, eno	K01689	EC:4.2.1.11
aldose 1-epimerase	galM, GALM	K01785	EC:5.1.3.3
glucose-6-phosphate isomerase	GPI, pgi	K01810	EC:5.3.1.9
2,3-bisphosphoglycerate-dependent phosphoglycerate mutase	PGAM, gpmA	K01834	EC:5.4.2.11
phosphoglucomutase	pgm	K01835	EC:5.4.2.2
fructose-1,6-bisphosphatase II	glpX	K02446	EC:3.1.3.11
fructose-1,6-bisphosphatase I	FBP, fbp	K03841	EC:3.1.3.11
fructose-1,6-bisphosphatase III	fbp3	K04041	EC:3.1.3.11
glucose-6-phosphate isomerase, archaeal	pgi1	K06859	EC:5.3.1.9
ADP-dependent glucokinase	ADPGK	K08074	EC:2.7.1.147
glyceraldehyde-3-phosphate dehydrogenase, spermatogenic	GAPDHS	K10705	EC:1.2.1.12
fructose-1,6-bisphosphatase II / sedoheptulose-1,7-bisphosphatase	glpX-SEBP	K11532	EC:3.1.3.11 3.1.3.37
fructose-bisphosphate aldolase, class I	fbaB	K11645	EC:4.1.2.13
glucokinase	GCK	K12407	EC:2.7.1.2
transaldolase / glucose-6-phosphate isomerase	tal-pgi	K13810	EC:2.2.1.2 5.3.1.9
2,3-bisphosphoglycerate-independent phosphoglycerate mutase	gpmI	K15633	EC:5.4.2.12
2,3-bisphosphoglycerate-dependent phosphoglycerate mutase	gpmB	K15634	EC:5.4.2.11
2,3-bisphosphoglycerate-independent phosphoglycerate mutase	apgM	K15635	EC:5.4.2.12
phosphomannomutase / phosphoglucomutase	pmm-pgm	K15778	EC:5.4.2.8 5.4.2.2
phosphoglucomutase / phosphopentomutase	PGM2	K15779	EC:5.4.2.2 5.4.2.7
glucose/mannose-6-phosphate isomerase	pgi-pmi	K15916	EC:5.3.1.9 5.3.1.8
fructose-bisphosphate aldolase / 6-deoxy-5-ketofructose 1-phosphate synthase	K16305	K16305	EC:4.1.2.13 2.2.1.11
fructose-bisphosphate aldolase / 2-amino-3,7-dideoxy-D-threo-hept-6-ulosonate synthase	K16306	K16306	EC:4.1.2.13 2.2.1.10
6-phosphofructokinase 2	pfkB	K16370	EC:2.7.1.11
ATP-dependent phosphofructokinase / diphosphate-dependent phosphofructokinase	pfk, pfp	K21071	EC:2.7.1.11 2.7.1.90
6-phosphofructokinase	PFK9	K24182	EC:2.7.1.11
phosphoglycerate kinase	PGK, pgk	K00927	EC:2.7.2.3
glyceraldehyde-3-phosphate dehydrogenase [NAD(P)+]	gapN	K18978	EC:1.2.1.90
glyceraldehyde-3-phosphate dehydrogenase (ferredoxin)	gapor	K11389	EC:1.2.7.6

Supplementary Table 6B. Distribution of metabolic genes in genomes of ANME-1, Ca. Veteromethanophagaceae and Ca. Alkanophagaceae, based on KofamScan and GhostKOALA annotations. Numbers >1 indicate that multiple gene clusters have the same annotation. Numbers in red indicate that the same gene cluster is found more than once in the genome. The location of each gene in the pangenome is reported on the left, "CORE" in black indicates that the gene is exclusively found in the core pangenome, "ACCESSORY" indicates that the gene is only found in the accessory pangenome, while "CORE" in blue indicates that the gene is generally found in the core pangenome, but for some genomes it is part of the accessory pangenome.

			KofamScan																																															
			QEXZ01												ANME-1a			WJOV0		ME-1-T		JACGMN01			QENH01						QENJ01					G60ANME1												Veter.		Alk.		
modules_with_ko	ko	location in pangenome	Barite_M4_B1	Barite_M1_B61	Barite_M3_B26	Barite_M2_B22	INS_M14_B50	Flange_M5_B8	B64_G16	WYZ_LMO12	WYZ_LMO13	GoMg1	Barite_M1_B78	M5_MMPM	G37ANME1	ex4572_4	HyVt_198	1244_F3_H4_B6	CR_Bin_179	ANME_1_THS	Kmv05	GoMg4	ANME-1-LC	SpSt_1198	UWMA-0191	CONS3730B06UFb1	GLR107	GoMg3.2	AG-394-G06	AG-394-G21	GoMg2	CONS3730H04p2b1	CONS3730F07p2b1	Agg-C03	CONS3730MDAH03UFb1	G60ANME1	B48_G6	Barite_M4_B48	Barite_M2_B26	Barite_M2_B29	Barite_M1_B69	Barite_M4_B44	Chimney19_Bin_00366	INS_M10_B59	INS_M11_B44	INS_M12_B72	INS_M14_B57	Chimney19_MAG_00329	B22_G9	Flange_M5_B38	B39_G2	HyVt_11
mcrA	K00399	CORE																																																			
mcrA2	K00400	CORE	1			1	1	1	1	1	1	1	1		1	1	1	1		1	1	1	1	1	1	1	1	1	1	1	1	1	1		1		1	1	1	1	1	1	1	1	1	1	1	1	1		1	1
mcrB	K00401	CORE	2						1	2	1	1		1	1	1			1	1	1	1	1	1		1	1									1	1		1		1		1		1		1	1		1	1	1
mcrG	K00402	CORE	1						1		1										1			1		1										1	1	1	1				1				1		1			
mcrC	K03421																																																			
mcrD	K03422																																																			
mtrA	K00577	CORE	2				1	2	1	1	1	1		1	1	1		2	1	1	2	1		2	1	1	1		1	1	1	1			1	1	1		1		1		1	1	1	1	1	1				
mtrB	K00578																																																1				
mtrC	K00579																																																			
mtrD	K00580	CORE					1	1	1	1	1	1		1	1			1	1					1	1	1	1		1	1						1							1					1				
mtrE	K00581																																																			
mtrF	K00582								1		1				1	1		1	1							1						1			1		1						1	1	1	1	1					
mtrG	K00583																																																			
mtrH	K00584	CORE	2			1	1		1	1	1	1		1	1	1		1	1	1	1	1	1	1	1	1	1		1	1	1	1			1	1	1		1				1	1	1	1	1	1				
mer	K00320	ACCESSORY												1									1																											1	2	2
fae/hps	K13812	CORE	1	1	1	1	1	1	1	1	1		1		1	1	1		1	1	1		1	1	1	1	1	1	1	1	1	1	1		1	1	1	1	1	1	1	1	1	1	1	1	1	1			1	
metF	K00297	CORE																																																	1	
mtd	K00319	CORE	1	2	1	1		1	1	1	1	1		1	1		1		1	1	1	1		1		1	1			1	1	1	1	1	1	1	1	1	1	1		1	1	1	1	1	1	1	1	1	1	1
mch	K01499	CORE	1	1	1	1	1	1	1	1	1	1	1		1		1	1	1	1	1	1	1	1	1	1	1		1	1		1	1		1	1		1	1	1	1	1	1	1	1	1	1	1	1		1	
ftr	K00672	CORE	1	2	2	2	2	2	2	1	1	2	1	2	2	1	2	2	2	2	2	1	1	2	2	2	1	1	2	1	2	2	2	1	2	2	1	2	2	1	1	1	2	2	2	2	2	2	1	1	1	
fwdA	K00200	CORE			1	1		1	1	1	1	1		2	1	1	1		1	1	1		1	1	2	1	1		1	1	1	1	1	1	1	1	1	1	1	1	1		1	1	1	1	1	1	1	1	1	1
fwdB	K00201	CORE	2	2	3	3	3	3	2	1	2	1	2	1	2	1	1		2	1	1	1	1	1	3	3	3	2	2	2	2	2	2	3	4	1	4	2	2	2	2	1	2	2	2	2	2	2	2	3	3	3
fwdC	K00202	CORE	1		1	1		1	1	1	1	1		1	1		1	1	1	2	1		1	1	2	1	1		1	1		1	1	1	1	1		1	1	1	1		1	1	1	1			1	1	1	
fwdD	K00203	CORE	1		1	1	1	1	1	1	1	1	1	1	1	1			1		1	2	1	1	1	1	1	1	1	1	1		2	1	2	1	1	1	1	1	1	1	1	1	1	1	1	1	1		1	1
fwdE	K11261	ACCESSORY	3	2	2	1					1	1	1	1				1				1	2	1	3	4	2		2	2		1	1	2	2								1	1						1	2	1
fwdF	K00205	ACCESSORY																					1																									1	1	1	1	1
fwdG	K11260	CORE	1	1	1	1	1	1	1										1				1		1	1		1	1		1	1	1	1	2			1	1	1	1	1	1	1	1					1	2	2
cdhB	K00193	CORE	1		1	1	1	1	1	1	1	1	1		1	1				1	1		1	1	1	1	1	1			1				1	1	1	1	1	1	1	1	1	1	1	1	1	1	1		1	1
cdhD	K00194	CORE	1	1	1	1	1	1	1	1	1	1	1	1	1	1	1			1	1		1	1	1	1	1	1	1	1		1	1		1	1	1	1	1	1	1	1	1	1	1	1	1	1	1		1	1
cdhG	K00197	CORE	1	1	1	1				2	1	1	1	1	1	1	1			1	1		1	1		1	1	1	1	1		1	1		1	1	1	1	1	1	1	1	1	1	1	1	1	1	1		2	1
cdhA	K00192	CORE	2	2	2	1	2	2	2		1	1	1	2	1	1	1		2	1	1		1	1	2	2	2	3	2	1	2	1	2	1	2	1	3	1	1	1	1	2	2	2	2	2	2	1		3	4	4
cdhE	K00195	CORE	1	1	1			1	1	1	1	1	1	1	1	1	1		1		1		2	1	1	1	1	1	1	1	1				1	1	1	1	1			1	1	1	1	1	1	1		2	2	2
cooS, acsA	K00198	ACCESSORY																		1	1			1																				1	1	1	1	1	1		1	

cooF	K00196	ACCESSORY						1																																								1	1	2	2	3
ACSS1_2, acs	K01895		1	1	1	1		1	1	1	1	1	1	1	1				1	2	1			2	2	1	1	1	1	1	1	1	1		1					1	1	1										
acdA	K01905																																																			
acdB	K22224																																																			
acdAB	K24012		2	2	1	1	1	1	1	2	1	2								1	1			1		1		1	2	2					1	1	1		1	1	1	1	1	1	1	1	1	1	1		1	
hdrA1	K22480																																																			
hdrB1	K22481																																																			
hdrC1	K22482																																																			
hdrA2	K03388	CORE	4		4	1	2	5	5	1	2	3	2	2	2	1	1	3	5	1	3		1	1	1	4	2	3	3	3	1	2	2	1	1	2	1	3	3	2	1	4	3	3	3	3	3	2	1	1	2	1
hdrB2	K03389	CORE			1	1	1	1	2	3	2	2	2	2	1	1	1		1	2	2		1	2	1	1	1		2	2	1			1	1	1	2	2	2		1	1	3	2	2	2	2	2	1	1	2	1
hdrC2	K03390	CORE	2	1	2	1	2	2	3	3	2	2	2	2	2	1	2	1	2	2	2	1	1	2	2	2	2	1	2	2	2	1	2		2	2	2	2	2	2	1	2	3	1	1	1	2	2	1	1	2	1
hdrD	K08264	CORE	1	1	2	2		2		1	1	1		1	1	1	1	2	3	1	1		1	1	1	2	1	1			1		1		1	2	1	1	1				1	1	1	1		1		1	4	2
hdrE	K08265																																																			
fdhA	K22516	ACCESSORY																					1																													1
fdhB	K00125	CORE	2	1	2	2	2	2	2	2	1	1	1	1	1	1			2	1	1			2	1	2	1	1	1	1	1	1	2		3	1	1	1	1	1	1	1	1	1	1	1	1	2	2	1	2	1
ATPVA	K02117	CORE	1	1	1	1	1		1	1		1	1	1	1	1	1		1		1	1	1	1	1	1	1	1		1		1	1		1	1	1	1	1	1			1	1	1	1	1	1	1		1	
ATPVB	K02118	CORE	1	1	1				1	1	1	1	1	1	1	1	1			1	1	1		1	1	1	1	1		1		1	1		1	1		1	1	1	1	1	1	1	1	1	1	1			1	1
ATPVC	K02119	CORE	1	1	1	1	1	1	1	1		1	1	1	1	1	1	1	1	1		1	1	1	1	1	1	1		1	1			1	1	1	1	1	1		1	1	1	1	1	1	1		1		1	1
ATPVD	K02120	CORE	1	1	1	1	1	1	1	1	1		1	1	1	1			1	1	1	1	1	1	1	1	1				1	1	1	1	1	1	1	1	1	1	1	1	1	1	1	1	1	1	1	1	1	
ATPVE	K02121	CORE	1	2	1	1		1	1	1	1	1		1	1	1			1	1	1			1	1	1	1	1	1	1	1		1	1	1	1		1	1	1	1		1	1	1	1	1	1	1		1	
ATPVF	K02122	CORE		2	1	1		1	1	1	1	1						1	1	1	1		1	1	1	1	1	1	1	1	1	1	1	1		1	1			1	1		1	1	1	1	1	1	1		1	
ATPVI	K02123	CORE	1	1	1	1	1	1	1		2	1	1		1	1	1	1	1	2		1	1	1	1	1	1	1		1	1					1	1	1	1	1		1	1	1	1	1	1	1	1		1	1
ATPVK	K02124	ACCESSORY																		1		1	1																													
fqoA	K22171	CORE	1	1	1	1	1	1	1	1				1	1		1		1	1	1	1		1		1		1		1		1	1	1	1	1	1	1	1	1	1	1	1	1	1	1	1	1	1		1	1
fqoBC	K22172	CORE	1	1	1	1	1	1	1	1				1					1	1	1	1		1								1	1	1	1	1				1		1	1	1	1	1	1		1			
fqoH	K22175	CORE																																																1		
fqoI	K22176	CORE	1		1	1	1	1	1	1	1			1	1	1	1		1	1	1	1		1		1	1	1		1	1	1	1	1	1		1	1	1	1	1		1	1	1	1	1	1	1	1	1	1
fqoJ	K22177	CORE	1		1	1	1	1	1	1				1	1	1	1		1	1	1	1		1	1	1	1			1		1	1	1		1		1	1	1	1	1	1	1	1	1	1	1		1	1	1
fqoK	K22178	CORE	1		1	1	1	1	1	1				1	1	1	1		1	1	1	1		1	1	1	1			1		1	1	1		1		1	1	1	1	1	1	1	1	1	1	1		1	1	1
fqoL	K22179	CORE		1	1	1				1				1	1	1	1		1	1	1	1		1		1				1			1			1	1	1	1	1			1	1	1	1	1		1		1	
fqoM	K22180	CORE		1	1	1			1	1				1	1	1	1		1	1	1			1		1	1			1		1	1	1		1		1	1	1	1		1	1	1	1	1			1	1	
fqoN	K22181	CORE	1	1	1	1		1	1	1				1	1	1	1		1	1	1	1		1		1		1		1		1	1	1	1	1	1	1	1	1	1	1	1	1	1	1		1	1		1	
rnfA	K03617	ACCESSORY																		1	1	1																														
rnfB	K03616	ACCESSORY																		1	1	1																														
rnfC	K03615	ACCESSORY																		1	1	1																														
rnfD	K03614	ACCESSORY																		1	1	1																														
rnfE	K03613	ACCESSORY																		1	1	1																														
rnfG	K03612	ACCESSORY																		1	1	1																														
frhB	K00441	CORE	5	4	4	4	3	6	5	3	4	7	4	6	5	5	2	1	8	4	6	2	1	5	3	1	6	7	4	8	8	6	6	2	9	7	3	5	6	5	5	5	6	5	5	5	5	5	4	3	4	3
mvhA	K14126	ACCESSORY																			1		1																												1	1
mvhD	K14127	CORE	3	1	3	3	2	5	5	2	2	2	2	2	2	2	2	1	4	2	5		1	3	2	4	2	2	2	2	2	1	2		4	2	1	2	2	3	2	4	2	2	2	2	2	3	3	2	5	4
mvhG	K14128	ACCESSORY																			1		1																												1	1
porA	K00169	CORE	1		1	1	1	1	1	2	1	2	2	2	1	1	1	1	1	1	1	1		2		2	2		1	2	1	1	1	1	1	1	1	1	1	1	1	1	1	1	1	1	1	1		1	2	2
porB	K00170	CORE	1			1		1	1	1	1	2	2	3	1	1	1	1	1	1	1	1		2		2	2	2	1	2	1	1	2	1	3	1	1	1	1	1	1	1	1	1	1	1	1	1			2	2
porD	K00171	CORE		1	1	1	2	1	1	1	1	1		1	1	1	1		1	1	1	1		2		1	1			1		1			1	1	1	1	1		1	1	1	1	1	1	1	1	1	1	1	2
porC	K00172	CORE			1		2	1	1	1	1	1		2	1	1	1		1		1	1		2		2	2	1	1	2	1	2	2	1	1	1	1	1	1		1	1	1	1	1	1	1	1	1	1	2	2
CcmB	K02194	CORE	1	1	1		1	1	1			1		1	1	1			1						1		1			1	1	1			1	1	1	1	1	1		1	1	1	1	1	1	1	1			
ccmC	K02195	CORE	1	1	1	1	1	1	1			1	1		1		1		1						1	1	1	1	1	1	1	1	1	1	1	1	1	1	1	1	1	1	2	1	1	1	1					

CcmE	K02197	CORE	1	1	1	1	1		1			1	1	1	1	1	1								1	1	1	1	1	1						1	1	1	1	1	1	1	1	1	1	1	1	1	1			
ccdA	K06196	CORE	1	1	1	1	1	1	1	1		1														1	1	1	1	1		1			1	1					1		1	1	1	1	1					
mtaA	K14080																																																				
mtaB	K04480																																																				
mtaC	K14081																																																				
hemE	K01599	ACCESSORY																																																		
mmgC	K18244																																																				
ACADM, acd	K00249	CORE	2	2	2	2	2	1	1	1	1	1		1	1	1	1	1	2	1	1	1	1		2	2	1	2	1	2	1		1	1	1	1		1	1	1	1	1	1	1	1	1	1	1	1	1	8	7
ACAT, atoB	K00626	CORE	2	2	2	2	2	2	2	1	2	2	2	2	2	2	2		2	2	2		2	2	2	2	2	2	2	1	2	1	2	1	2	2	2	2	2	2	2	2	2	2	2	2	2	2	2		5	5
mmgB	K00074	CORE							1		1	1										1										1	1	1		1		1	1	1	1	1	1	1	1	1	1	1		1	1	1
HADH	K00022																																																		1	
	K08683																																																			
crt	K01715	CORE	1	1	1		1	1	1	1	1	1	1		1		1		1		1	1	1	1	1	1		1				1				1		1	1	1	1	1	1	1	1	1	1			1	3	1
paaF, echA	K01692	CORE	1	1	1		1	1	1	1	1	1	1		1		1		1		1	1	1	1	1	1		1			1	1	1			1		1	1	1	1	1	1	1	1	1	1	1		1	5	2
etfA	K03522	ACCESSORY																																															1	2	2
etfB	K03521	ACCESSORY																																															2	2	2

(continues from Supplementary Table 6B)

	GhostKOALA																																																	
	QEXZ01												ANME-1a			WJOV0		ME-1-T		JACGMN01			QENH01						QENJ01					G60ANME1												Veter.		Alk.		
	Barite_M4_B1	Barite_M1_B61	Barite_M3_B26	Barite_M2_B22	INS_M14_B50	Flange_M5_B8	B64_G16	WYZ_LMO12	WYZ_LMO13	GoMg1	Barite_M1_B78	M5_MMPM	G37ANME1	ex4572_4	HyVt_198	1244_F3_H4_B6	CR_Bin_179	ANME_1_THS	Kmv05	GoMg4	ANME-1-LC	SpSt_1198	UWMA-0191	CONS3730B06UFb1	GLR107	GoMg3.2	AG-394-G06	AG-394-G21	GoMg2	CONS3730H04p2b1	CONS3730F07p2b1	Agg-C03	CONS3730MDAH03UFb1	G60ANME1	B48_G6	Barite_M4_B48	Barite_M2_B26	Barite_M2_B29	Barite_M1_B69	Barite_M4_B44	Chimney19_Bin_00366	INS_M10_B59	INS_M11_B44	INS_M12_B72	INS_M14_B57	Chimney19_MAG_00329	B22_G9	Flange_M5_B38	B39_G2	HyVt_11
mcrA	1					1	1	1	1	1		1	1	1	1		1	1	1	1	1	1		1	1		1	1		1	1	1	1	1			1				1		1		1	1	1	2	2	2
mcrA2	2		1	2	2	1	1	2	2	2	2	2	2	2	1	1	2	2	2	2	2	2	2	2	2	2	1	2	1	2	2	2	2	1	1	2	2	2	2	2	2	2	2	2	2	2	1	2	2	2
mcrB					1			2	1	1		1	1	1			1		1	1	1	1		1	1	1	2							1	1	1	1	2	1		1		1		1	1	1	1	2	1
mcrG	1						1	2	1	1		1	1	1			1	1		1	1	1		2	1	1	1	1				1	2	1		1	1	1	1		1		1		1		1	2	1	1
mcrC	2				1		1	1	1	1	1	1	1	1		1	1	1	1	1	1	1	1	1	1	1	1	1	1	1			1	1		1	1	1	1	1	1	1	1	1	1	1				
mcrD																																																		
mtrA	2				1	1		1	1	1		1	1	1		2	1	1	2	1		2	1	1	1		1	1	1	1	1		1	1			1	1	1		1	1	1	1	1	1				
mtrB																																																		
mtrC	1				1		1	1	1	1		1	1	1		1	1	1	1	1		1	1	1	1		1	1	1	1	1		1	1			1	1	1		1	1	1	1	1	1				
mtrD	1				1	1	1	1	1	1		1	1	1		1	1	1	1	1		1	1	1	1		1	1		1	1		1	1			1	1	1		1	1		1	1	1				
mtrE	1				1	1	1	1	1	1		1	1	1			1	1	1	1		1	1	1	1	1	1	1		1	1	1	1	1			1	1	1		1	1	1	1	1	1				
mtrF																																																		
mtrG																																			1															
mtrH	2			1	1	1	1	1	1	2		3	1	1	1	1	1	1	1	1	1	1	1	1	1	1	1	1	1	1			1	1			1	1			1	1	1	1	1	1				
mer																																			1													1	1	1
fae/hps	1	1	1	1	1	2	1	1	1				1	1	1		1	1	1		1	1	1	1	1	1	1	1	1	1	1		1	1	1	1	1	1	1	1	1	1	1	1	1	1			1	
metF	2		3	1	1	2	1	2	1		2	1	1	1	1		1	1	2			1		1	1	1	1	1	1	1		1	1	2		1	1	2	1	2	1	1	1	1	1	1	1		1	1
mtd	1	2	1	1	1	1		1	1	1		1	1		1		1	1	1	1	1	1		1	1			1	1	1	1	1	1	1		1	1			1	1	1	1	1	1	1		1		1
mch	1	1	1	1	1	1	1	1	1	1	1	1	1		1	1	1	1	1	1	1	1	1	1	1	1	1	1		1	1		1	1	2	1	1		1	1	1	1	1	1	1	1	1		1	
ftr	3	2	2	2	1	1	2	2	1	2	1	2	2	1	2	2	2	2	2	1	1	1	2	2	3	2	2	1	1	2	2	1	2	2		2	2	1	2	2	2	2	2	2	2	2	1	1	1	
fwdA	2	1	2	2	1	2	2	1	2	1		2	3	3	2	2	2	1	1		1	1	4	2	2	1	2	2	2	2	2	1	2	1		2	2	2	2	1	2	2	2	2	2	2	1		2	1
fwdB	3	3	3	3	3	3	2	1	2		2	2	2		1		2	1		2	1	1	3	3	3	2	2	2	2	3	3	3	4	1	1	2	2	3	2	2	3	2	2	2	2	3	1	2	3	3
fwdC	1		2	1		1	1	2	3	1	2	2	2	2	1	2	1	3	1			1	2	2	1		2	1		1	1	1	1	2	1	2	2	2	2	1	2	2	2	2	2	1	2	1	1	
fwdD	1		1	1	1	1	1	1	1	1	1	1	1	1			1		1	2		1	1	1	1	1	1	1	1		2	1	2	1		1	1	1	1	1	1	1	1	1	1	1			1	1
fwdE		1	1						1			1				1				1		1	1	2			1	1			1	1	1		1						1	1							2	2
fwdF																																			1											1			1	1
fwdG																																			2	1	1		1	1	1	1	1					1	2	2
cdhB	2	1	2	2	2	2	2	2	2	1	1	1	2	2	1		2	1	1		1	1	2	2	2	2	1	3	2	1	3	1	2	1	1	2	2	3	2	2	2	2	2	1	2	1		3	4	4
cdhD	1	1	1	1	1	1	1	1	1	1	1	1	1	1	1	1		1	1		1	1	1	1	1	1	1	1		1	1		1	1		1	1	1	1	1	1	1	1	1	1	1	1	1	2	2

cdhG	1	1	1	1		1		2	1	1	1	1	1	1	1	2		1	1		1	1		1	1	1	1	1		1	1		1	1	2	1	1	1	1	2	2	2	2	2	2	1	1	2	2	1
cdhA	3	2	2	1	2	2	2	1	1	1	1	2	2	2	1	1	2	1	1		1	1	2	1	2	2	2	2	2	2	2	1	2	1		1	1	2	1	1	2	2	2	2	2	1		3	4	6
cdhE	1	1	1			1	1	1	1	1	1	1	1	1	1		1				1	1	1	1	1	1	1	1	1				1	1		1	1			1	1	1	1	1	1	1		1	2	2
cooS, acsA																		1	1			1											1									1	1	1	1				1	1
cooF						1															1																					1	1	1	1				2	2
ACSS1_2, acs	1	1	1	1		1	1	1	1	2	1	3	1	1	2		1	2	1		1	3	3	2	1	1	1	2	1	1	2	1	2					1	1	1								2		
acdA																																																		
acdB										1												1		1	1								2																1	1
acdAB	1	1			1	1		1	1	2		1						1	1	1		3		2	1	1	2	2		3	3	2	3	1		2	1		1	1	1	1	1	1	1	1			5	6
hdrA1																																																		
hdrB1	2		1	2			1					1				1		1									1	1						1				1			1	1	1	1	1		1			
hdrC1																																																		
hdrA2	6	5	4	3	6	9	5	5	5	7	6	8	6	7	8	5	10	7	14		1	6	7	9	4	8	5	6	4	4	10	6	11	7	2	6	5	5	8	10	6	5	5	5	5	7	2	8	6	9
hdrB2	2	2	2	1	2	2	2	3	2	2	2	2	2	2	2	1	2	2	2	1	1	2	2	2	1	2	2	2	3	2	3	3	3	2		2	2	1	1	2	2	2	2	2	2	2		1	2	2
hdrC2	2	1	2	2	2	2	2	3	2	2	2	2	2	2	2	1	2	2	2	1	1	2	2	2	2	1	2	2	2	1	2		2	2	1	2	2	2	2	2	2	2	2	2	2	2	1	1	2	2
hdrD	1		1	1										1					1		1									1			1				1			1	1				2	1			1	1
hdrE																																																		
fdhA							1																	1						2	1		1															1	1	1
fdhB	2	1	2	2	3	2	2	2	1	1	1	1	1	1	1		2	1	1			2	1	2	1	1	1	1	1	1	2		2	1		1	1	1	1	1	1	1	1	1	1	2		2	2	1
ATPVA	1	1	1	1	1	1	1	2	1	1	1	1	1	1	1		1	1	1	1	2	1	1	1	1	1	1	1		1	1		1	1		1	1	1	1	2	1	1	1	1	1	1	1		1	1
ATPVB	1	1	1	1	2	1	1	2	1	1	2	2	1	1	1		1	1	1	1	1	1	1	1	1	1	1	1		1	1		1	1	1	1	1	1	1	1	1	1	1	1	1	1	1		1	1
ATPVC								1		1	1	1					1									1			1			1	1	1		1	1													
ATPVD	1	1	1	1	1	1	1	1	1		1	1	1	1			1	1	1	1	1	1	1	1	1				1	1	1	1	1	1		1	1		1	1	1	1	1	1	1	1		1	1	
ATPVE																																			1															
ATPVF	1	2	1	1		1	1	1	1	1			1	1		1	1	1	1		1	1	1	1	1	1	1	1	1	1	1	1	1	1		1	1		1		1	1	1	1	1	1	1		1	
ATPVI	2	1	1	1	1	1	1		2	1	1		1	1	1	1	1	2		1	2	1	1	1	1	1	1	1	1	1		1	1	1	1	1	1	2	1	1	1	1	1	1	1	2	1	1	1	1
ATPVK					1	1	1			1		1	1		1	1	2	2		1	1	1		2	1	1	1		1	1	1	1		1	1	1	1		1	1	1	1	1	1	1	1	1	1	1	1
fqoA																																																		
fqoBC																																																		
fqoH																																																		
fqoI																																																		
fqoJ																																																		
fqoK																																																		
fqoL																																																		
fqoM																																																		
fqoN																																																		
rnfA																		1	1	1																														
rnfB																		1	1	1																														
rnfC																		1	1	1																														
rnfD																		1	1	1																														
rnfE																		1	1	1																														
rnfG																		1	1	1																														
frhB	6	4	3	2	2	3	2	3	4	7	4	5	2	4	1	3	6	5	3	2	1	4	1	7	5	5	4	6	6	4	5	3	7	5		3	3	5	3	3	4	3	3	3	3	5	2	4	3	5
mvhA																			1		1																												1	1

mvhD	3	1	3	3	3	5	4	2	2	2	2	2	2	2	2	1	3	2	5		1	2	2	4	2	2	2	2	2	1	2		4	2		2	2	2	2	4	3	2	2	2	2	3	1	2	4	5
mvhG																			1		1																												1	1
porA	1		1	1	1	1	1	2	1	2	2	3	1	1	2	1	1	1	1	1		2		2	2	2	1	2	1	1	1	1	1	1		1	1		1	1	1	1	1	1	1	1		1	2	2
porB	1		1	1	1	1	1	2	2	2	2	2	1	1	1	1	1	1	1	1		2		2	3	3	1	1	1	1	2	1	3	1	1	1	1		1	1	1	1	1	1	1	1	1	1	2	2
porD		1	1	1	1	1	1	1	1			2	1	1	1		1	1	1					1	1			1	1	1	1		1	1		1	1		1	1	1	1	1	1	1	1		1	2	1
porC	1		1	1	1	1	1	1	2	1	2	2	1	1	1		1	1	1	1		2		2	2	1	1	2	1	2	2	1	1	1	1	1	1		1	1	1	1	1	1	1	1		1	2	2
CcmB																																																		
ccmC	1	1	1	1	1	1	1			1	1		1		1		1						1	1	1	1	1	1	1	1	1	1	1	1	1	1			1	1	2	1	1	1	1	1				
CcmE	1	1	1	1			1			1	1	1	1	1	1		1						1	1	1	1	1	1	1					1	1				1	1	1	1	1	1	1	1				
ccdA	1	1	1	1	1	1	1	1		1	1								1					1	1	1	1	1		1	1		1	1	2			1	1		1	1	1	1	1					
mtaA																																																	2	1
mtaB																																																		
mtaC																																																		
hemE																																																	2	2
mmgC							1	1								1	1						1	1	1	1	1			1		1	1																1	
ACADM, acd																																																		
ACAT, atoB	2	2	2	2	2	2	1	1	3	2	2	3	2	2	2		2	2	2		2	2	2	1	1	2	2	1	2	1	2	1	2	2		2	2	1	2	2	2	2	2	2	2	2	1	1	5	5
mmgB	1	1	1	1	1	1	1	1	1	1	1	1	1	1	1		1		1	1	1	1	1	1	1	1	1	1	1	1	1	1	1	1		1	1	1	1	1	1	1	1	1	1	1		2	2	2
HADH																																																		
crt	1	1	1	1			1	1	1	1	1		1		1		1		1	1	1	1	1	1		1			2	1	1		1	1		1	1		1	1	1	1	1	1	1	1		1	3	3
paaF, echA																																																		
etfA																																																1	2	2
etfB																																																1	2	2
