# Supplementary material for: Phylogenetic and functional diverse ANME-1 thrive in Arctic hydrothermal vents
Source: FEMS Microbiol Ecol. 2022 Oct 3;98(11):fiac117. doi: 10.1093/femsec/fiac117 (PMC9576274; doi:10.1093/femsec/fiac117)
Supplement: fiac117_Supplemental_Files [file fiac117_supplemental_files.zip › Supp_data_Table_7.pdf]

Supplementary Table 7: Completeness of the main cellular pathways (as Kegg module) in ANME-1 genera, *Ca* . Veterometahnophagacea and *Ca* . Alkanophagaceae; the pathways completeness in genomes of Syntropharcheales and ANME-2 is also analyzed and compared (highlighted in blue and yellow, respectively). The matrix is obtained by using the anvi'o (v.7) function anvi-estimate-metabolism.

		QEXZ01												ANME1a		WJOV01		NME-1-TH		JACGMN01			QENH01						QENJ01				
module	module_name	Barite_M1_B61	Barite_M4_B1	Barite_M2_B22	Barite_M3_B26	Flange_M5_B8	INS_M14_B50	B64_G16	WYZ_LMO12	WYZ_LMO13	GoMg1	Barite_M1_B78	M5_MMPM	G37ANME1	HyVt_198	1244_F3_H4_B6	CR_Bin_179	ANME_1_THS	Kmv05	Gomg4	ANME-1-LC	SpSt_1198	UWMA-0191	CONS3730B06UFb1	GLR107	GoMg3.2	AG-394-G06	AG-394-G21	GoMg2	CONS3730H04p2b1	CONS3730F07p2b1	Agg-C03	CONS3730MDAH03UFb1
M00001	Glycolysis (Embden-Meyerhof pathway). glucose => pyruvate	0.4	0.7	0.6	0.6	0.8	0.7	0.7	0.7	0.6	0.6	0.4	0.7	0.7	0.7	0.5	0.7	0.5	0.7	0.2	0.6	0.7	0.6	0.9	0.8	0.8	0.7	0.7	0.4	0.7	0.7	0.4	0.6
M00002	Glycolysis. core module involving three-carbon compounds	0.4	0.8	0.6	0.7	0.8	0.7	0.8	0.6	0.6	0.7	0.5	0.8	0.8	0.8	0.5	0.8	0.7	0.8	0.4	0.7	0.8	0.8	0.8	0.8	0.8	0.5	0.7	0.5	0.7	0.7	0.4	0.7
M00003	Gluconeogenesis. oxaloacetate => fructose-6P	0.5	1.0	0.6	0.9	1.0	0.9	1.0	0.6	0.6	0.7	0.7	1.0	1.0	1.0	0.6	1.0	0.7	1.0	0.3	0.7	1.0	0.9	0.9	0.9	0.9	0.6	0.8	0.8	0.8	0.8	0.3	0.6
M00004	Pentose phosphate pathway (Pentose phosphate cycle)	0.3	0.3	0.3	0.3	0.3	0.3	0.3	0.3	0.3	0.3	0.3	0.3	0.3	0.1	0.1	0.4	0.1	0.3	0.1	0.1	0.3	0.1	0.3	0.3	0.3	0.1	0.1	0.1	0.3	0.3	0.1	0.3
M00005	PRPP biosynthesis. ribose 5P => PRPP	1.0	0.0	1.0	1.0	1.0	1.0	1.0	0.0	0.0	1.0	1.0	1.0	1.0	1.0	0.0	1.0	1.0	1.0	1.0	1.0	1.0	1.0	1.0	1.0	1.0	1.0	1.0	0.0	1.0	1.0	0.0	1.0
M00007	Pentose phosphate pathway. non-oxidative phase. fructose 6P => ribose 5P	0.3	0.3	0.3	0.3	0.3	0.3	0.3	0.3	0.3	0.3	0.3	0.3	0.3	0.0	0.0	0.3	0.3	0.3	0.3	0.0	0.3	0.0	0.3	0.3	0.3	0.0	0.0	0.3	0.3	0.3	0.0	0.3
M00009	Citrate cycle (TCA cycle. Krebs cycle)	0.1	0.2	0.3	0.3	0.4	0.3	0.4	0.2	0.1	0.4	0.3	0.4	0.3	0.2	0.1	0.3	0.3	0.3	0.2	0.1	0.5	0.1	0.3	0.3	0.3	0.1	0.3	0.2	0.3	0.2	0.1	0.3
M00010	Citrate cycle. first carbon oxidation. oxaloacetate => 2-oxoglutarate	0.0	0.3	0.3	0.3	0.7	0.7	0.7	0.3	0.0	0.7	0.7	0.7	0.3	0.3	0.0	0.3	0.3	0.7	0.3	0.0	0.7	0.0	0.3	0.3	0.3	0.3	0.3	0.3	0.3	0.3	0.0	0.3
M00011	Citrate cycle. second carbon oxidation. 2-oxoglutarate => oxaloacetate	0.2	0.1	0.2	0.2	0.2	0.1	0.2	0.1	0.2	0.2	0.0	0.2	0.2	0.1	0.2	0.2	0.3	0.1	0.1	0.2	0.4	0.2	0.2	0.2	0.2	0.0	0.2	0.1	0.2	0.1	0.1	0.2
M00015	Proline biosynthesis. glutamate => proline	1.0	1.0	1.0	1.0	1.0	1.0	0.3	1.0	0.7	1.0	0.7	1.0	1.0	0.7	0.5	1.0	1.0	1.0	0.3	1.0	1.0	0.5	1.0	1.0	0.7	0.3	1.0	0.7	0.7	1.0	0.7	1.0
M00016	Lysine biosynthesis. succinyl-DAP pathway. aspartate => lysine	0.6	0.9	0.6	0.9	0.8	0.9	0.8	0.4	0.4	0.9	0.6	0.7	0.9	0.8	0.4	0.9	0.8	0.9	0.7	0.8	0.9	0.9	0.9	0.8	0.7	0.3	0.4	0.8	0.6	0.7	0.4	0.7
M00017	Methionine biosynthesis. aspartate => homoserine => methionine	0.3	0.6	0.6	0.4	0.4	0.6	0.4	0.6	0.3	0.4	0.6	0.6	0.6	0.4	0.3	0.9	0.6	1.0	0.4	0.6	0.9	0.4	0.7	0.3	0.4	0.1	0.4	0.3	0.4	0.6	0.7	0.7
M00018	Threonine biosynthesis. aspartate => homoserine => threonine	0.4	0.8	0.6	0.8	0.6	0.6	0.8	0.8	0.6	0.6	0.8	0.8	0.8	0.6	0.6	0.8	0.8	0.8	0.6	0.8	0.8	0.6	0.8	0.6	0.6	0.4	0.4	0.4	0.6	1.0	0.8	0.8
M00019	Valine/isoleucine biosynthesis. pyruvate => valine / 2-oxobutanoate => isoleucine	0.8	1.0	0.5	0.8	0.8	0.4	1.0	0.4	0.8	1.0	0.5	1.0	1.0	1.0	0.0	1.0	0.8	1.0	0.5	1.0	1.0	1.0	1.0	0.6	0.5	0.6	0.9	0.8	1.0	0.8	0.8	1.0
M00020	Serine biosynthesis. glycerate-3P => serine	0.0	0.3	0.0	0.3	0.3	0.3	0.3	0.0	0.3	0.0	0.0	0.3	0.3	0.3	0.0	0.3	0.0	0.3	0.0	0.3	0.3	0.0	0.3	0.3	0.3	0.3	0.0	0.3	0.3	0.3	0.3	0.3
M00022	Shikimate pathway. phosphoenolpyruvate + erythrose-4P => chorismate	0.4	0.6	0.4	0.4	0.4	0.5	0.6	0.3	0.6	0.6	0.4	0.6	0.6	0.6	0.3	0.6	0.3	0.4	0.2	0.6	0.6	0.4	0.6	0.4	0.3	0.3	0.5	0.6	0.4	0.6	0.4	0.4
M00023	Tryptophan biosynthesis. chorismate => tryptophan	0.7	0.8	0.7	0.7	0.8	0.8	0.8	0.8	0.5	0.8	0.8	0.8	0.8	0.5	0.7	0.8	0.8	0.8	0.7	0.8	0.8	0.7	0.8	0.8	0.8	0.5	0.8	0.8	0.7	0.8	0.7	0.7
M00024	Phenylalanine biosynthesis. chorismate => phenylpyruvate => phenylalanine	0.3	0.3	0.0	0.3	0.0	0.3	0.3	0.3	0.3	0.3	0.3	0.3	0.3	0.3	0.3	0.3	0.3	0.3	0.3	0.3	0.3	0.0	0.3	0.3	0.3	0.0	0.3	0.0	0.3	0.0	0.3	0.0
M00025	Tyrosine biosynthesis. chorismate => HPP => tyrosine	0.0	0.3	0.0	0.0	0.3	0.0	0.3	0.0	0.0	0.3	0.0	0.3	0.3	0.3	0.3	0.3	0.3	0.3	0.3	0.3	0.3	0.3	0.0	0.3	0.0	0.3	0.0	0.0	0.0	0.3	0.3	0.0
M00026	Histidine biosynthesis. PRPP => histidine	0.6	0.6	0.8	0.8	0.8	0.7	0.6	0.7	0.7	0.6	0.8	0.7	0.7	0.6	0.4	0.7	0.8	0.6	0.5	0.7	0.6	0.7	0.8	0.8	0.6	0.4	0.4	0.6	0.7	0.7	0.3	0.8
M00028	Ornithine biosynthesis. glutamate => ornithine	0.8	1.0	1.0	1.0	1.0	0.8	1.0	0.4	0.6	1.0	0.6	0.8	1.0	1.0	1.0	1.0	0.8	1.0	0.5	1.0	1.0	0.8	1.0	1.0	1.0	0.4	0.6	0.4	1.0	1.0	0.6	1.0
M00029	Urea cycle	0.2	0.6	0.6	0.6	0.6	0.6	0.6	0.6	0.4	0.4	0.4	0.4	0.6	0.6	0.0	0.6	0.4	0.6	0.0	0.6	0.6	0.6	0.6	0.6	0.6	0.4	0.2	0.6	0.4	0.6	0.2	0.6
M00033	Ectoine biosynthesis. aspartate => ectoine	0.2	0.4	0.4	0.4	0.2	0.4	0.4	0.4	0.4	0.4	0.4	1.0	0.4	0.2	0.2	1.0	0.4	0.4	0.8	0.4	1.0	1.0	1.0	0.2	0.4	0.2	0.2	0.2	0.2	0.4	0.4	0.4
M00034	Methionine salvage pathway	0.3	0.3	0.3	0.3	0.4	0.5	0.4	0.4	0.3	0.5	0.1	0.3	0.3	0.3	0.3	0.3	0.3	0.3	0.3	0.1	0.3	0.3	0.5	0.5	0.4	0.4	0.4	0.5	0.5	0.5	0.5	0.3
M00035	Methionine degradation	0.5	0.5	0.5	0.5	0.3	0.5	0.5	0.5	0.3	0.5	0.3	0.5	0.5	0.5	0.0	0.8	0.8	0.5	0.8	0.5	0.8	0.3	0.5	0.5	0.8	0.5	0.3	0.3	0.5	0.5	0.3	0.8
M00036	Leucine degradation. leucine => acetoacetate + acetyl-CoA	0.3	0.3	0.2	0.2	0.3	0.3	0.3	0.3	0.2	0.3	0.0	0.3	0.3	0.3	0.2	0.3	0.2	0.3	0.2	0.3	0.2	0.3	0.3	0.3	0.3	0.3	0.3	0.3	0.2	0.2	0.3	0.3
M00048	Inosine monophosphate biosynthesis. PRPP + glutamine => IMP	0.6	0.6	0.5	0.6	0.6	0.7	0.7	0.6	0.4	0.6	0.7	0.6	0.8	0.6	0.6	0.9	0.7	0.8	0.4	0.8	0.8	0.6	0.6	0.7	0.4	0.4	0.5	0.7	0.7	0.6	0.5	0.6
M00049	Adenine ribonucleotide biosynthesis. IMP => ADP.ATP	0.5	0.5	0.8	0.8	1.0	0.8	0.8	0.3	0.0	0.8	0.5	0.5	1.0	1.0	0.8	1.0	1.0	0.8	1.0	1.0	1.0	0.8	0.8	1.0	0.5	0.8	1.0	0.8	0.8	0.8	0.8	1.0
M00050	Guanine ribonucleotide biosynthesis. IMP => GDP.GTP	0.5	0.5	0.5	0.5	0.5	0.5	0.5	0.3	0.3	0.5	0.3	0.5	0.5	0.5	0.3	0.5	0.5	0.5	0.3	0.5	0.5	0.3	0.5	0.5	0.3	0.3	0.5	0.5	0.3	0.5	0.5	0.5
M00051	Uridine monophosphate biosynthesis. glutamine (+ PRPP) => UMP	0.4	0.3	0.4	0.4	0.7	0.3	0.6	0.6	0.6	0.7	0.3	0.5	0.4	0.3	0.4	0.6	0.6	0.6	0.3	0.7	0.7	0.5	0.7	0.6	0.6	0.5	0.5	0.7	0.6	0.5	0.2	0.7
M00052	Pyrimidine ribonucleotide biosynthesis. UMP => UDP/UTP.CDP/CTP	1.0	0.7	1.0	1.0	1.0	1.0	1.0	0.7	0.7	0.7	1.0	0.7	1.0	1.0	1.0	1.0	0.7	1.0	0.7	1.0	1.0	0.3	1.0	0.7	0.3	1.0	0.7	0.7	0.7	0.7	1.0	0.7
M00053	Pyrimidine deoxyribonuleotide biosynthesis. CDP => dCTP	0.5	0.8	0.8	0.8	0.8	0.5	0.8	0.0	0.0	0.8	0.8	0.5	0.5	0.5	0.8	0.5	0.8	0.8	0.8	0.5	0.8	0.3	0.5	0.5	0.3	0.8	0.8	0.5	0.0	0.5	0.5	0.5
M00082	Fatty acid biosynthesis. initiation	0.0	0.0	0.0	0.0	0.0	0.0	0.0	0.0	0.0	0.0	0.0	0.0	0.0	0.0	0.0	0.4	0.0	0.0	0.0	0.0	0.0	0.0	0.0	0.0	0.0	0.0	0.0	0.0	0.0	0.0	0.0	0.0
M00083	Fatty acid biosynthesis. elongation	0.0	0.0	0.0	0.0	0.0	0.0	0.0	0.0	0.0	0.0	0.0	0.0	0.0	0.0	0.0	0.8	0.0	0.3	0.0	0.0	0.0	0.0	0.0	0.0	0.0	0.0	0.3	0.0	0.0	0.0	0.0	0.0
M00085	Fatty acid elongation in mitochondria	0.0	0.0	0.0	0.0	0.0	0.0	0.0	0.0	0.0	0.0	0.0	0.0	0.0	0.0	0.0	0.0	0.0	0.0	0.0	0.0	0.0	0.0	0.0	0.0	0.0	0.0	0.0	0.0	0.0	0.0	0.0	0.0
M00086	beta-Oxidation. acyl-CoA synthesis	0.0	0.0	0.0	0.0	0.0	0.0	0.0	0.0	0.0	0.0	0.0	0.0	0.0	0.0	0.0	0.0	0.0	0.0	0.0	0.0	0.0	0.0	0.0	0.0	0.0	0.0	0.0	0.0	0.0	0.0	0.0	0.0
M00087	beta-Oxidation	0.5	0.5	0.3	0.5	0.5	0.5	0.5	0.5	0.5	0.5	0.3	0.3	0.5	0.5	0.3	0.5	0.3	0.5	0.5	0.5	0.3	0.5	0.5	0.3	0.5	0.3	0.3	0.5	0.3	0.5	0.3	0.3
M00088	Ketone body biosynthesis. acetyl-CoA => acetoacetate/3-hydroxybutyrate/acetone	0.4	0.4	0.4	0.4	0.2	0.4	0.4	0.2	0.4	0.4	0.4	0.4	0.4	0.4	0.0	0.4	0.2	0.4	0.0	0.4	0.4	0.4	0.4	0.4	0.2	0.2	0.2	0.4	0.2	0.4	0.2	0.4
M00093	Phosphatidylethanolamine (PE) biosynthesis. PA => PS => PE	0.3	0.3	0.3	0.3	0.3	0.3	0.0	0.3	0.0	0.7	0.7	0.3	0.7	0.7	0.0	0.3	0.3	0.3	0.0	0.0	0.3	0.3	0.3	0.3	0.0	0.0	0.0	0.7	0.0	0.7	0.7	1.0
M00095	C5 isoprenoid biosynthesis. mevalonate pathway	0.4	0.6	0.4	0.6	0.4	0.6	0.6	0.4	0.4	0.6	0.4	0.4	0.6	0.6	0.1	0.7	0.4	0.6	0.1	0.6	0.6	0.6	0.4	0.6	0.4	0.3	0.3	0.7	0.4	0.6	0.3	0.6
M00096	C5 isoprenoid biosynthesis. non-mevalonate pathway	0.0	0.1	0.1	0.1	0.1	0.1	0.3	0.1	0.1	0.1	0.1	0.0	0.1	0.1	0.0	0.1	0.1	0.1	0.0	0.1	0.1	0.1	0.0	0.1	0.1	0.1	0.1	0.1	0.1	0.1	0.0	0.0
M00098	Acylglycerol degradation	0.0	0.0	0.0	0.0	0.0	0.0	0.0	0.0	0.5	0.0	0.0	0.0	0.0	0.0	0.0	0.0	0.0	0.0	0.0	0.0	0.0	0.0	0.0	0.0	0.5	0.0	0.0	0.0	0.0	0.0	0.0	0.0
M00114	Ascorbate biosynthesis. plants. fructose-6P => ascorbate	0.1	0.1	0.1	0.0	0.1	0.1	0.1	0.3	0.0	0.1	0.1	0.1	0.1	0.0	0.0	0.1	0.0	0.0	0.1	0.1	0.1	0.0	0.1	0.1	0.1	0.1	0.1	0.1	0.1	0.1	0.0	0.1
M00115	NAD biosynthesis. aspartate => quinolinate => NAD	0.6	0.8	0.8	0.8	0.6	0.8	0.8	0.6	0.4	0.8	0.6	0.8	0.8	0.6	0.4	0.6	0.8	0.8	0.6	0.8	0.8	0.6	0.8	0.8	0.6	0.4	0.8	0.8	0.6	0.8	0.6	0.8
M00119	Pantothenate biosynthesis. valine/L-aspartate => pantothenate	0.2	0.2	0.0	0.0	0.2	0.2	0.4	0.4	0.2	0.4	0.2	0.4	0.2	0.2	0.2	0.4	0.2	0.4	0.0	0.2	0.2	0.4	0.2	0.2	0.2	0.2	0.2	0.4	0.4	0.2	0.4	0.4
M00120	Coenzyme A biosynthesis. pantothenate => CoA	0.8	0.8	0.8	0.8	0.8	0.8	0.5	0.8	0.5	0.8	0.3	0.8	0.8	0.8	0.5	0.8	0.8	0.8	0.3	0.8	0.8	0.5	0.8	0.5	0.5	0.5	0.5	0.5	0.5	0.5	0.5	0.8
M00121	Heme biosynthesis. plants and bacteria. glutamate => heme	0.2	0.3	0.2	0.4	0.5	0.3	0.4	0.3	0.4	0.5	0.4	0.5	0.5	0.3	0.2	0.6	0.1	0.5	0.5	0.5	0.5	0.3	0.5	0.5	0.4	0.3	0.3	0.4	0.3	0.4	0.4	0.4
M00122	Cobalamin biosynthesis. cobyrinate a.c-diamide => cobalamin	0.4	0.7	0.6	0.7	0.7	0.6	0.6	0.6	0.6	0.4	0.6	0.7	0.6	0.4	0.4	0.6	0.6	0.6	0.1	0.6	0.3	0.4	0.7	0.7	0.6	0.4	0.6	0.4	0.6	0.6	0.4	0.6
M00123	Biotin biosynthesis. pimeloyl-ACP/CoA => biotin	0.0	0.0	0.0	0.0	0.0	0.0	0.0	0.0	0.0	0.0	0.0	0.0	0.0	0.0	0.0	0.0	0.0	0.0	0.0	0.0	0.0	0.0	0.0	0.0	0.0	0.0	0.0	0.0	0.0	0.0	0.0	0.0
M00124	Pyridoxal-P biosynthesis. erythrose-4P => pyridoxal-P	0.0	0.0	0.0	0.0	0.0	0.0	0.0	0.0	0.0	0.0	0.0	0.0	0.0	0.0	0.0	0.0	0.0	0.0	0.0	0.0	0.2	0.0	0.2	0.0	0.0	0.0	0.0	0.0	0.2	0.0	0.0	0.2
M00125	Riboflavin biosynthesis. plants and bacteria. GTP => riboflavin/FMN/FAD	0.0	0.0	0.0	0.1	0.1	0.1	0.1	0.1	0.1	0.1	0.1	0.1	0.1	0.1	0.1	0.1	0.1	0.1	0.0	0.1	0.1	0.1	0.1	0.1	0.1	0.1	0.1	0.1	0.1	0.1	0.0	0.1
M00126	Tetrahydrofolate biosynthesis. GTP => THF	0.0	0.2	0.0	0.0	0.0	0.0	0.2	0.0	0.0	0.0	0.0	0.0	0.0	0.0	0.0	0.0	0.2	0.0	0.0	0.0	0.0	0.0	0.0	0.0	0.0	0.0	0.0	0.0	0.0	0.2	0.0	0.0
M00127	Thiamine biosynthesis. prokaryotes. AIR (+ DXP/tyrosine) => TMP/TPP	0.0	0.1	0.3	0.1	0.3	0.3	0.4	0.0	0.1	0.3	0.3	0.3	0.3	0.3	0.3	0.3	0.3	0.4	0.2	0.4	0.3	0.3	0.4	0.3	0.4	0.2	0.1	0.4	0.2	0.3	0.2	0.3
M00129	Ascorbate biosynthesis. animals. glucose-1P => ascorbate	0.0	0.0	0.0	0.0	0.1	0.0	0.0	0.0	0.0	0.1	0.0	0.0	0.0	0.0	0.1	0.1	0.1	0.1	0.1	0.0	0.0	0.1	0.3	0.1	0.1	0.3	0.1	0.1	0.1	0.1	0.1	0.1
M00133	Polyamine biosynthesis. arginine => agmatine => putrescine => spermidine	0.0	0.5	0.3	0.3	1.0	1.0	0.5	0.8	0.3	0.8	0.3	0.5	0.5	0.3	1.0	0.5	0.5	0.5	0.3	0.3	0.5	0.5	1.0	0.8	1.0	0.5	0.5	0.8	1.0	1.0	0.5	0.8
M00140	C1-unit interconversion. prokaryotes	0.3	0.3	0.3	0.3	0.3	0.3	0.3	0.3	0.3	0.3	0.3	0.3	0.3	0.3	0.3	0.7	0.7	0.3	0.0	0.3	0.3	0.3	0.3	0.3	0.3	0.3	0.3	0.3	0.0	0.3	0.3	0.3
M00141	C1-unit interconversion. eukaryotes	0.5	0.5	0.5	0.5	0.5	0.5	0.5	0.5	0.5	0.5	0.5	0.5	0.5	0.5	0.5	0.5	0.5	0.5	0.0	0.5	0.5	0.5	0.5	0.5	0.5	0.5	0.5	0.5	0.0	0.5	0.5	0.5
M00144	NADH:quinone oxidoreductase. prokaryotes	0.1	0.1	0.0	0.2	0.0	0.2	0.1	0.2	0.2	0.2	0.2	0.2	0.2	0.1	0.2	0.3	0.1	0.1	0.0	0.2	0.2	0.2	0.2	0.2	0.0	0.2	0.2	0.3	0.2	0.0	0.1	0.4
M00159	V/A-type ATPase. prokaryotes	0.9	0.8	0.8	0.9	0.7	0.4	0.9	0.8	0.6	0.7	0.6	0.6	0.7	0.4	0.3	0.7	0.8	0.7	0.7	0.8	0.8	0.8	0.8	0.8	0.7	0.2	0.7	0.6	0.6	0.6	0.4	0.7
M00165	Reductive pentose phosphate cycle (Calvin cycle)	0.1	0.3	0.1	0.3	0.3	0.3	0.3	0.3	0.1	0.3	0.3	0.5	0.3	0.2	0.4	0.3	0.3	0.3	0.1	0.1	0.5	0.2	0.5	0.5	0.5	0.3	0.4	0.6	0.5	0.5	0.0	0.3
M00166	Reductive pentose phosphate cycle. ribulose-5P => glyceraldehyde-3P	0.0	0.5	0.0	0.5	0.5	0.5	0.5	0.0	0.0	0.5	0.5	0.5	0.5	0.5	0.3	0.5	0.5	0.5	0.0	0.3	0.5	0.5	0.5	0.5	0.5	0.3	0.5	0.5	0.5	0.5	0.0	0.5
M00167	Reductive pentose phosphate cycle. glyceraldehyde-3P => ribulose-5P	0.1	0.1	0.1	0.1	0.1	0.1	0.1	0.4	0.1	0.1	0.1	0.4	0.1	0.0	0.4	0.1	0.1	0.1	0.1	0.0	0.4	0.0	0.4	0.4	0.4	0.3	0.3	0.6	0.4	0.4	0.0	0.1
M00168	CAM (Crassulacean acid metabolism). dark	0.0	0.5	0.0	0.5	0.0	0.0	0.5	0.5	0.0	0.0	0.0	0.0	0.0	0.0	0.0	0.0	0.0	0.0	0.0	0.5	0.0	0.0	0.0	0.0	0.0	0.0	0.0	0.0	0.0	0.0	0.0	0.0
M00169	CAM (Crassulacean acid metabolism). light	0.0	0.0	0.0	0.0	0.0	0.0	0.0	0.0	0.0	0.0	0.0	0.0	0.0	0.0	0.0	0.0	0.0	0.0	0.0	0.0	0.0	0.0	0.0	0.0	0.0	0.0	0.0	0.0	0.0	0.0	0.0	0.5
M00170	C4-dicarboxylic acid cycle. phosphoenolpyruvate carboxykinase type	0.0	0.3	0.0	0.3	0.0	0.0	0.3	0.3	0.0	0.0	0.0	0.0	0.0	0.0	0.0	0.0	0.0	0.0	0.0	0.3	0.0	0.0	0.0	0.0	0.0	0.0	0.0	0.0	0.0	0.0	0.0	0.0
M00171	C4-dicarboxylic acid cycle. NAD - malic enzyme type	0.0	0.1	0.0	0.1	0.0	0.0	0.1	0.1	0.0	0.0	0.0	0.0	0.0	0.0	0.0	0.0	0.0	0.0	0.0	0.1	0.0	0.0	0.0	0.0	0.0	0.0	0.0	0.0	0.0	0.0	0.0	0.0
M00172	C4-dicarboxylic acid cycle. NADP - malic enzyme type	0.0	0.3	0.0	0.3	0.0	0.0	0.3	0.3	0.0	0.0	0.0	0.0	0.0	0.0	0.0	0.0	0.0	0.0	0.0	0.3	0.0	0.0	0.0	0.0	0.0	0.0	0.0	0.0	0.0	0.0	0.0	0.3
M00173	Reductive citrate cycle (Arnon-Buchanan cycle)	0.2	0.3	0.3	0.3	0.4	0.3	0.5	0.4	0.3	0.4	0.2	0.4	0.3	0.2	0.2	0.3	0.4	0.4	0.4	0.3	0.5	0.2	0.4	0.4	0.3	0.3	0.4	0.1	0.4	0.3	0.2	0.4
M00174	Methane oxidation. methanotroph. methane => formaldehyde	0.0	0.0	0.0	0.0	0.0	0.0	0.0	0.0	0.0	0.0	0.0	0.0	0.0	0.0	0.0	0.0	0.0	0.0	0.0	0.0	0.0	0.0	0.0	0.0	0.0	0.0	0.0	0.0	0.0	0.0	0.0	0.0
M00175	Nitrogen fixation. nitrogen => ammonia	0.3	0.3	0.3	0.3	0.3	0.3	0.3	0.3	0.3	0.7	0.3	0.3	0.0	0.0	0.0	0.3	0.3	0.3	0.3	0.3	0.3	0.0	0.7	0.3	0.7	0.3	0.7	0.7	0.3	0.7	0.7	0.7
M00176	Assimilatory sulfate reduction. sulfate => H2S	0.2	0.2	0.5	0.2	0.5	0.5	0.2	0.0	0.0	0.0	0.0	0.0	0.3	0.3	0.0	0.5	0.2	0.0	0.0	0.0	0.0	0.0	0.5	0.0	0.0	0.2	0.0	0.0	0.0	0.3	0.0	0.0
M00307	Pyruvate oxidation. pyruvate => acetyl-CoA	0.3	0.5	0.8	0.8	1.0	0.8	1.0	1.0	1.0	1.0	0.5	1.0	1.0	1.0	0.5	1.0	0.8	1.0	1.0	0.0	1.0	0.0	1.0	1.0	0.5	0.8	1.0	0.8	1.0	0.8	0.8	1.0
M00308	Semi-phosphorylative Entner-Doudoroff pathway. gluconate => glycerate-3P	0.0	0.4	0.0	0.4	0.4	0.4	0.6	0.0	0.0	0.4	0.4	0.4	0.4	0.4	0.2	0.4	0.4	0.4	0.0	0.2	0.4	0.4	0.4	0.4	0.4	0.2	0.4	0.4	0.4	0.4	0.0	0.4
M00309	Non-phosphorylative Entner-Doudoroff pathway. gluconate/galactonate => glycerate	0.0	0.3	0.3	0.3	0.0	0.3	0.3	0.3	0.3	0.3	0.3	0.3	0.3	0.3	0.0	0.3	0.3	0.3	0.3	0.0	0.3	0.3	0.3	0.3	0.0	0.3	0.3	0.3	0.3	0.3	0.3	0.3
M00338	Cysteine biosynthesis. homocysteine + serine => cysteine	0.0	0.0	0.0	0.0	0.0	0.0	0.0	0.0	0.0	0.0	0.0	0.0	0.0	0.0	0.0	0.0	0.0	0.5	0.0	0.0	0.0	0.0	0.0	0.0	0.0	0.0	0.0	0.0	0.0	0.0	0.0	0.0
M00344	Formaldehyde assimilation. xylulose monophosphate pathway	0.0	0.0	0.0	0.0	0.0	0.0	0.0	0.3	0.0	0.0	0.0	0.3	0.0	0.0	0.5	0.0	0.0	0.0	0.0	0.0	0.3	0.0	0.3	0.3	0.3	0.3	0.3	0.5	0.3	0.3	0.0	0.0
M00345	Formaldehyde assimilation. ribulose monophosphate pathway	0.3	0.3	0.3	0.3	0.7	0.5	0.3	1.0	0.3	0.3	0.3	0.7	0.3	0.3	0.3	0.3	0.3	0.3	0.3	0.3	0.7	0.3	0.8	1.0	0.8	1.0	0.8	0.5	0.8	1.0	0.3	0.3
M00346	Formaldehyde assimilation. serine pathway	0.3	0.4	0.3	0.4	0.2	0.2	0.3	0.4	0.3	0.1	0.1	0.2	0.2	0.2	0.1	0.2	0.2	0.2	0.0	0.3	0.2	0.2	0.2	0.3	0.3	0.1	0.1	0.1	0.1	0.2	0.2	0.2
M00356	Methanogenesis. methanol => methane	0.2	0.4	0.3	0.3	0.3	0.3	0.6	0.4	0.6	0.4	0.3	0.4	0.4	0.3	0.2	0.4	0.4	0.6	0.2	0.4	0.6	0.3	0.6	0.4	0.2	0.3	0.3	0.3	0.2	0.2	0.2	0.3
M00357	Methanogenesis. acetate => methane	0.4	0.7	0.6	0.6	0.6	0.4	0.8	0.8	0.8	0.8	0.6	0.7	0.8	0.3	0.2	0.6	0.7	0.8	0.2	0.5	0.8	0.6	0.8	0.8	0.5	0.6	0.6	0.5	0.5	0.5	0.1	0.7
M00358	Coenzyme M biosynthesis	0.4	0.6	0.5	0.6	0.5	0.5	0.5	0.0	0.0	0.5	0.0	0.6	0.3	0.0	0.0	0.0	0.0	0.0	0.3	0.6	0.0	0.0	0.0	0.6	0.0	0.1	0.6	0.0	0.0	0.0	0.0	0.0
M00364	C10-C20 isoprenoid biosynthesis. bacteria	0.0	0.5	0.5	0.5	0.5	0.5	0.5	0.5	0.5	0.5	0.5	0.0	0.5	0.5	0.0	0.5	0.5	1.0	0.0	1.0	1.0	1.0	0.5	0.5	1.0	0.5	1.0	0.5	0.5	0.5	0.0	0.0
M00365	C10-C20 isoprenoid biosynthesis. archaea	0.0	0.5	0.5	0.5	0.5	0.5	0.5	0.5	0.5	0.5	0.5	0.0	0.5	0.5	0.0	0.5	0.5	1.0	0.0	1.0	1.0	1.0	0.5	0.5	1.0	0.5	1.0	0.5	0.5	0.5	0.0	0.0
M00366	C10-C20 isoprenoid biosynthesis. plants	0.0	0.3	0.3	0.3	0.3	0.3	0.3	0.3	0.3	0.3	0.3	0.0	0.3	0.3	0.0	0.3	0.3	0.3	0.0	0.3	0.3	0.3	0.0	0.3	0.3	0.3	0.3	0.3	0.3	0.3	0.0	0.0
M00367	C10-C20 isoprenoid biosynthesis. non-plant eukaryotes	0.0	0.3	0.3	0.3	0.3	0.3	0.3	0.3	0.3	0.3	0.3	0.0	0.3	0.3	0.0	0.3	0.3	0.3	0.0	0.3	0.3	0.3	0.0	0.3	0.3	0.3	0.3	0.3	0.3	0.3	0.0	0.0
M00368	Ethylene biosynthesis. methionine => ethylene	0.3	0.3	0.3	0.3	0.3	0.3	0.3	0.3	0.0	0.3	0.0	0.3	0.3	0.3	0.0	0.3	0.3	0.3	0.3	0.3	0.3	0.3	0.3	0.3	0.3	0.3	0.3	0.3	0.3	0.3	0.3	0.3
M00373	Ethylmalonyl pathway	0.1	0.1	0.1	0.1	0.1	0.1	0.1	0.1	0.1	0.1	0.1	0.1	0.1	0.1	0.0	0.1	0.1	0.1	0.0	0.1	0.1	0.1	0.1	0.1	0.1	0.1	0.1	0.1	0.1	0.1	0.1	0.1

	G60ANME1												*Ca* . Veteron		*Ca* . Alkanoph.			*Ca* . Syntroph.			ANME-2													
module	G60ANME1	B48_G6	Barite_M4_B48	Barite_M2_B26	Barite_M1_B69	Barite_M4_B44	Barite_M2_B29	Chimney19_Bin_00366	INS_M14_B57	INS_M11_B44	INS_M12_B72	INS_M10_B59	Chimney19_MAG_00329	B22_G9	B39_G2	HyVt_11	Flange_M5_B38	BOX2	HyVt_386	WYZ_LMO15	GCA_002254785_1	GCA_002503595.	GCA_002506015.1	GCA_002926195.1	GCA_003194445.1	GCA_003601535.1	GCA_003661105.1	GCA_004193545.1	GCA_004211975.1	GCA_004212095.1	GCA_012961645.1	GCA_013572335.1	GCA_013572355.1	GCA_014237145.1
M00001	0.8	0.6	0.8	0.8	0.7	0.8	0.6	0.8	0.8	0.8	0.8	0.8	0.7	0.5	0.7	0.7	0.4	0.8	0.7	0.7	0.6	0.9	1.0	0.8	1.0	1.0	0.9	0.8	1.0	0.8	0.9	0.6	0.8	0.7
M00002	0.8	0.7	0.8	0.8	0.8	0.8	0.7	0.8	0.8	0.8	0.8	0.8	0.8	0.5	0.8	0.8	0.5	0.8	0.8	0.8	0.8	1.0	1.0	1.0	1.0	1.0	0.8	0.8	1.0	0.8	0.8	0.7	1.0	0.8
M00003	1.0	0.9	1.0	1.0	0.8	1.0	0.8	1.0	1.0	1.0	1.0	1.0	1.0	0.6	1.0	1.0	0.6	0.9	0.9	0.9	1.0	0.8	0.8	0.9	0.8	0.8	0.6	0.6	0.8	0.6	0.8	0.9	0.8	1.0
M00004	0.3	0.3	0.3	0.3	0.3	0.3	0.3	0.3	0.3	0.3	0.3	0.3	0.1	0.3	0.3	0.3	0.3	0.3	0.3	0.1	0.0	0.1	0.3	0.3	0.3	0.3	0.3	0.1	0.3	0.1	0.1	0.1	0.3	0.3
M00005	1.0	1.0	1.0	1.0	1.0	1.0	1.0	1.0	1.0	1.0	1.0	1.0	1.0	1.0	1.0	1.0	0.0	1.0	1.0	1.0	1.0	1.0	1.0	1.0	1.0	1.0	1.0	1.0	1.0	1.0	1.0	0.0	1.0	1.0
M00007	0.3	0.3	0.3	0.3	0.3	0.3	0.3	0.3	0.3	0.3	0.3	0.3	0.0	0.3	0.3	0.3	0.3	0.3	0.3	0.0	0.0	0.3	0.3	0.3	0.3	0.3	0.3	0.3	0.3	0.3	0.0	0.0	0.3	0.3
M00009	0.3	0.1	0.3	0.3	0.3	0.3	0.3	0.3	0.3	0.3	0.3	0.3	0.1	0.1	0.2	0.2	0.2	0.1	0.3	0.3	0.3	0.2	0.3	0.5	0.3	0.2	0.1	0.4	0.3	0.4	0.1	0.3	0.3	0.6
M00010	0.3	0.0	0.3	0.3	0.3	0.3	0.3	0.3	0.3	0.3	0.3	0.3	0.3	0.0	0.3	0.3	0.3	0.3	0.7	0.7	0.3	0.3	0.3	0.3	0.3	0.3	0.3	0.3	0.3	0.3	0.3	0.3	0.3	0.3
M00011	0.2	0.1	0.2	0.2	0.2	0.2	0.2	0.2	0.2	0.2	0.2	0.2	0.0	0.1	0.1	0.1	0.1	0.0	0.0	0.0	0.2	0.1	0.2	0.6	0.2	0.1	0.0	0.4	0.2	0.4	0.0	0.3	0.2	0.7
M00015	1.0	1.0	1.0	1.0	0.7	1.0	1.0	1.0	1.0	1.0	1.0	1.0	0.0	0.5	0.5	0.5	0.5	0.5	1.0	1.0	1.0	0.7	1.0	1.0	1.0	1.0	0.5	0.5	1.0	0.0	1.0	0.5	0.7	1.0
M00016	0.9	0.7	0.8	0.9	0.4	0.6	0.9	0.9	0.9	0.9	0.9	0.9	0.8	0.4	0.8	0.7	0.4	0.8	0.7	0.7	0.6	0.6	0.7	0.7	0.8	0.8	0.6	0.7	0.7	0.7	0.6	0.8	0.7	0.8
M00017	0.4	0.3	0.4	0.6	0.3	0.3	0.4	0.6	0.6	0.6	0.6	0.6	0.6	0.3	0.9	0.6	0.3	0.7	0.6	0.6	0.6	0.6	0.3	0.3	0.6	0.4	0.3	0.9	0.6	0.7	0.7	0.4	0.4	0.9
M00018	0.8	0.6	0.6	0.8	0.2	0.6	0.6	0.8	0.8	0.8	0.8	0.8	0.8	0.4	0.8	0.8	0.4	0.8	0.8	0.8	0.8	0.8	0.4	0.6	0.8	0.6	0.4	0.8	0.8	0.6	0.8	0.8	0.6	0.8
M00019	1.0	1.0	1.0	1.0	1.0	1.0	1.0	1.0	1.0	1.0	1.0	1.0	0.8	0.8	0.3	0.3	0.0	1.0	1.0	1.0	1.0	1.0	1.0	1.0	1.0	1.0	1.0	1.0	1.0	1.0	1.0	0.8	1.0	1.0
M00020	0.3	0.0	0.3	0.3	0.3	0.3	0.3	0.3	0.3	0.3	0.3	0.3	0.3	0.3	0.7	0.7	0.0	0.7	0.3	0.3	0.0	0.7	0.7	0.3	0.7	0.7	0.7	0.7	0.7	0.7	0.7	0.3	0.7	0.3
M00022	0.6	0.4	0.4	0.4	0.6	0.4	0.4	0.6	0.6	0.6	0.6	0.6	0.4	0.3	0.4	0.6	0.2	0.6	0.4	0.4	0.6	0.6	0.6	0.6	0.4	0.6	0.5	0.4	0.4	0.6	0.4	0.3	0.6	0.6
M00023	0.7	0.7	0.7	0.7	0.7	0.5	0.6	0.7	0.7	0.7	0.7	0.7	0.6	0.7	0.8	0.6	0.5	0.8	0.8	0.8	0.5	0.7	0.7	0.8	0.8	0.4	0.8	0.8	0.6	0.8	0.6	0.8	0.8	0.7
M00024	0.3	0.3	0.3	0.3	0.0	0.3	0.3	0.3	0.3	0.3	0.3	0.3	0.3	0.3	0.3	0.3	0.3	0.0	0.3	0.3	0.3	0.3	0.3	0.3	0.3	0.3	0.0	0.3	0.3	0.0	0.3	0.3	0.3	0.3
M00025	0.3	0.0	0.3	0.3	0.3	0.3	0.0	0.3	0.3	0.3	0.3	0.3	0.3	0.3	0.3	0.3	0.0	0.0	0.3	0.3	0.0	0.3	0.3	0.3	0.3	0.3	0.3	0.3	0.3	0.3	0.3	0.0	0.3	0.3
M00026	0.8	0.6	0.6	0.8	0.7	0.8	0.6	0.6	0.8	0.8	0.8	0.8	0.4	0.8	0.8	0.4	0.4	0.8	0.7	0.8	0.7	0.8	0.5	0.6	0.8	0.5	0.6	0.6	0.6	0.8	0.7	0.7	0.8	0.8
M00028	1.0	1.0	1.0	1.0	0.2	0.8	0.6	1.0	1.0	1.0	1.0	1.0	0.8	0.6	1.0	1.0	1.0	1.0	1.0	0.6	0.6	0.8	1.0	1.0	1.0	1.0	0.8	0.8	0.8	1.0	0.2	1.0	1.0	1.0
M00029	0.6	0.4	0.6	0.6	0.2	0.6	0.4	0.6	0.6	0.6	0.6	0.6	0.4	0.4	0.6	0.4	0.4	0.6	0.6	0.6	0.4	0.8	0.6	0.6	0.6	0.6	0.4	0.6	0.6	0.6	0.4	0.2	0.6	0.6
M00033	0.4	0.2	0.2	0.4	0.0	0.2	0.4	0.4	0.4	0.4	0.4	0.4	0.4	0.0	0.4	0.4	0.2	0.4	0.4	0.4	0.4	0.4	0.2	0.2	0.4	0.4	0.2	0.4	0.4	0.4	0.4	0.4	0.4	0.4
M00034	0.3	0.1	0.1	0.3	0.3	0.3	0.3	0.4	0.3	0.3	0.3	0.3	0.4	0.4	0.5	0.5	0.3	0.4	0.3	0.4	0.0	0.3	0.3	0.3	0.5	0.1	0.3	0.4	0.3	0.4	0.1	0.3	0.3	0.3
M00035	0.5	0.3	0.3	0.5	0.5	0.5	0.5	0.8	0.5	0.5	0.5	0.5	0.3	0.5	0.8	0.8	0.3	0.5	0.5	0.5	0.3	0.5	0.5	0.5	0.5	0.5	0.5	0.5	0.5	0.3	0.3	0.5	0.3	0.8
M00036	0.3	0.2	0.3	0.3	0.3	0.3	0.3	0.3	0.3	0.3	0.3	0.3	0.3	0.2	0.3	0.3	0.2	0.3	0.3	0.3	0.3	0.2	0.2	0.2	0.2	0.2	0.2	0.2	0.2	0.2	0.2	0.2	0.2	0.2
M00048	0.9	0.6	0.7	0.7	0.8	0.7	0.7	0.8	0.8	0.8	0.8	0.8	0.5	0.7	0.6	0.6	0.3	0.6	0.7	0.7	0.6	0.9	0.9	0.9	0.9	0.9	0.8	0.9	0.9	1.0	0.4	0.9	0.9	1.0
M00049	1.0	0.5	0.8	1.0	1.0	1.0	1.0	1.0	1.0	1.0	1.0	1.0	1.0	1.0	0.5	0.5	0.3	0.8	1.0	1.0	0.5	1.0	1.0	1.0	1.0	1.0	1.0	1.0	1.0	1.0	0.8	1.0	1.0	1.0
M00050	0.5	0.5	0.5	0.5	0.5	0.5	0.5	0.5	0.5	0.5	0.5	0.5	0.5	0.5	0.5	0.5	0.3	0.5	0.5	0.5	0.5	0.5	0.5	0.5	0.5	0.5	0.5	0.5	0.5	0.5	0.5	0.5	0.5	0.5
M00051	0.6	0.4	0.6	0.5	0.4	0.5	0.5	0.6	0.6	0.6	0.6	0.6	0.6	0.4	0.7	0.4	0.3	0.8	0.8	0.8	0.4	0.8	0.8	1.0	0.8	0.8	0.7	0.9	0.8	0.8	0.8	0.6	0.8	1.0
M00052	1.0	0.7	1.0	1.0	1.0	1.0	1.0	1.0	1.0	1.0	1.0	1.0	1.0	1.0	1.0	1.0	0.7	1.0	1.0	1.0	0.7	1.0	1.0	1.0	1.0	0.7	1.0	1.0	1.0	1.0	1.0	0.7	1.0	1.0
M00053	0.8	0.8	0.8	0.8	0.5	0.8	0.8	0.8	0.8	0.8	0.8	0.8	0.5	0.5	0.5	0.5	0.0	0.8	0.8	0.8	0.5	0.8	0.5	0.8	0.5	0.8	0.5	0.8	0.5	0.5	0.5	0.8	0.8	0.8
M00082	0.0	0.0	0.0	0.0	0.0	0.0	0.0	0.0	0.0	0.0	0.0	0.0	0.0	0.0	0.0	0.0	0.0	0.2	0.0	0.2	0.0	0.0	0.2	0.0	0.2	0.2	0.2	0.2	0.2	0.0	0.0	0.0	0.2	0.0
M00083	0.0	0.0	0.0	0.0	0.0	0.0	0.0	0.0	0.0	0.0	0.0	0.0	0.0	0.0	0.3	0.3	0.0	0.0	0.0	0.0	0.0	0.0	0.0	0.3	0.0	0.0	0.0	0.0	0.3	0.0	0.0	0.0	0.0	0.0
M00085	0.0	0.0	0.0	0.0	0.0	0.0	0.0	0.0	0.0	0.0	0.0	0.0	0.0	0.0	0.3	0.0	0.0	0.3	0.3	0.3	0.0	0.0	0.0	0.0	0.0	0.0	0.0	0.0	0.0	0.0	0.0	0.0	0.0	0.0
M00086	0.0	0.0	0.0	0.0	0.0	0.0	0.0	0.0	0.0	0.0	0.0	0.0	0.0	0.0	0.0	1.0	0.0	0.0	0.0	0.0	0.0	0.0	0.0	0.0	0.0	0.0	0.0	0.0	0.0	0.0	0.0	0.0	0.0	0.0
M00087	0.5	0.0	0.5	0.5	0.5	0.5	0.5	0.5	0.5	0.5	0.5	0.5	0.5	0.3	0.8	0.5	0.5	0.8	0.8	0.8	0.3	0.0	0.0	0.0	0.0	0.0	0.0	0.0	0.0	0.0	0.0	0.0	0.0	0.0
M00088	0.4	0.2	0.4	0.4	0.4	0.4	0.4	0.4	0.4	0.4	0.4	0.4	0.4	0.2	0.4	0.4	0.2	0.4	0.4	0.4	0.4	0.4	0.2	0.4	0.4	0.4	0.4	0.4	0.4	0.4	0.4	0.4	0.4	0.4
M00093	0.7	0.7	0.7	0.7	0.3	0.7	0.3	0.7	0.3	0.3	0.7	0.3	0.0	0.3	0.7	0.7	0.3	0.0	0.0	0.0	0.7	0.7	0.7	0.7	0.7	0.7	0.7	0.7	0.7	0.7	0.7	0.7	0.7	0.7
M00095	0.7	0.3	0.7	0.7	0.4	0.6	0.6	0.6	0.6	0.6	0.6	0.6	0.6	0.3	0.6	0.6	0.4	0.6	0.6	0.6	0.6	0.6	0.4	0.7	0.4	0.6	0.6	0.7	0.6	0.6	0.6	0.7	0.6	0.6
M00096	0.1	0.0	0.1	0.1	0.0	0.0	0.0	0.1	0.0	0.0	0.0	0.0	0.0	0.0	0.1	0.1	0.1	0.1	0.1	0.1	0.1	0.1	0.1	0.1	0.0	0.1	0.1	0.1	0.1	0.1	0.1	0.1	0.1	0.0
M00098	0.0	0.0	0.0	0.0	0.0	0.0	0.0	0.0	0.0	0.0	0.0	0.0	0.0	0.0	0.0	0.0	0.0	0.0	0.0	0.0	0.0	0.0	0.0	0.0	0.0	0.0	0.0	0.0	0.0	0.0	0.0	0.0	0.0	0.0
M00114	0.1	0.1	0.1	0.1	0.1	0.1	0.1	0.1	0.1	0.1	0.1	0.1	0.1	0.1	0.1	0.1	0.0	0.1	0.1	0.1	0.1	0.0	0.1	0.0	0.0	0.1	0.1	0.0	0.0	0.0	0.1	0.0	0.1	0.0
M00115	0.6	0.6	0.6	0.6	0.8	0.2	0.6	0.6	0.8	0.8	0.8	0.8	0.6	0.8	0.8	0.4	0.8	0.8	0.6	0.8	0.8	0.8	0.8	0.8	0.8	0.8	0.8	0.6	0.8	0.6	0.6	0.6	0.8	0.8
M00119	0.4	0.2	0.2	0.2	0.2	0.2	0.2	0.4	0.4	0.4	0.4	0.4	0.4	0.2	0.4	0.4	0.2	0.4	0.4	0.4	0.2	0.4	0.4	0.2	0.4	0.2	0.4	0.2	0.4	0.2	0.4	0.2	0.4	0.2
M00120	0.8	0.5	0.8	0.5	0.8	0.8	0.5	0.8	0.8	0.8	0.8	0.8	0.5	0.3	0.3	0.5	0.5	0.5	0.5	0.5	0.8	0.5	0.0	0.5	0.5	0.5	0.0	0.5	0.5	0.5	0.3	0.5	0.5	0.5
M00121	0.5	0.2	0.4	0.5	0.5	0.4	0.5	0.5	0.5	0.5	0.5	0.5	0.5	0.3	0.5	0.5	0.1	0.6	0.6	0.6	0.5	0.6	0.6	0.5	0.6	0.6	0.4	0.5	0.5	0.4	0.3	0.3	0.5	0.5
M00122	0.6	0.6	0.6	0.7	0.3	0.7	0.7	0.7	0.7	0.7	0.7	0.7	0.3	0.4	0.7	0.3	0.0	0.7	0.7	0.7	0.3	0.4	0.4	0.4	0.4	0.4	0.3	0.6	0.4	0.3	0.1	0.1	0.4	0.4
M00123	0.0	0.0	0.0	0.0	0.0	0.0	0.0	0.0	0.0	0.0	0.0	0.0	0.0	0.0	0.0	0.0	0.0	0.0	0.0	0.0	0.0	0.0	0.0	0.0	0.0	0.0	0.0	0.8	0.0	0.8	0.0	0.0	0.0	0.0
M00124	0.0	0.0	0.0	0.0	0.0	0.0	0.0	0.0	0.0	0.0	0.0	0.0	0.0	0.0	0.0	0.0	0.0	0.2	0.0	0.0	0.0	0.0	0.2	0.0	0.2	0.0	0.2	0.0	0.0	0.0	0.0	0.0	0.0	0.0
M00125	0.1	0.1	0.1	0.1	0.1	0.0	0.1	0.1	0.1	0.1	0.1	0.1	0.1	0.1	0.1	0.1	0.0	0.1	0.1	0.1	0.1	0.1	0.1	0.1	0.1	0.1	0.1	0.0	0.1	0.1	0.1	0.1	0.1	0.1
M00126	0.0	0.0	0.0	0.0	0.0	0.0	0.0	0.0	0.0	0.0	0.0	0.0	0.2	0.0	0.0	0.0	0.0	0.0	0.0	0.0	0.0	0.0	0.0	0.2	0.0	0.0	0.0	0.2	0.0	0.2	0.0	0.0	0.0	0.2
M00127	0.4	0.1	0.4	0.4	0.1	0.4	0.3	0.2	0.4	0.4	0.4	0.4	0.3	0.3	0.6	0.3	0.0	0.4	0.4	0.4	0.1	0.6	0.3	0.6	0.6	0.6	0.6	0.6	0.4	0.6	0.6	0.4	0.6	0.6
M00129	0.0	0.0	0.0	0.0	0.1	0.0	0.0	0.1	0.0	0.0	0.0	0.0	0.0	0.0	0.0	0.0	0.0	0.1	0.0	0.0	0.0	0.3	0.3	0.1	0.3	0.1	0.3	0.3	0.0	0.3	0.3	0.0	0.3	0.1
M00133	0.5	0.5	0.3	0.0	0.5	0.5	0.5	0.8	0.5	0.5	0.5	0.5	1.0	0.5	0.8	0.5	0.0	0.3	0.3	0.3	0.5	0.5	0.3	0.3	0.8	0.3	0.0	0.3	0.5	0.3	0.3	0.3	0.3	0.3
M00140	0.3	0.3	0.3	0.3	0.3	0.3	0.3	0.3	0.3	0.3	0.3	0.3	0.3	0.0	0.0	0.0	0.3	0.3	0.3	0.3	0.3	0.3	0.3	0.7	0.3	0.0	0.3	0.7	0.3	0.7	0.3	0.3	0.3	0.7
M00141	0.5	0.5	0.5	0.5	0.5	0.5	0.5	0.5	0.5	0.5	0.5	0.5	0.5	0.0	0.0	0.0	0.5	0.5	0.5	0.5	0.5	0.5	0.5	0.5	0.5	0.0	0.5	0.5	0.5	0.5	0.5	0.5	0.5	0.5
M00144	0.2	0.2	0.1	0.2	0.2	0.0	0.2	0.2	0.2	0.2	0.2	0.2	0.0	0.0	0.0	0.0	0.0	0.6	0.6	0.6	0.2	0.0	0.2	0.6	0.5	0.3	0.1	0.4	0.3	0.6	0.4	0.6	0.3	0.6
M00159	0.9	0.7	0.8	0.8	0.6	0.4	0.8	0.9	0.9	0.9	0.9	0.9	0.8	0.8	0.9	0.3	0.1	0.9	0.9	0.9	0.7	0.9	0.9	0.9	1.0	0.8	0.9	0.9	0.9	0.9	0.6	1.0	0.9	1.0
M00165	0.5	0.5	0.3	0.3	0.3	0.5	0.2	0.5	0.5	0.5	0.5	0.5	0.2	0.2	0.3	0.3	0.2	0.4	0.4	0.3	0.2	0.5	0.5	0.6	0.5	0.3	0.5	0.2	0.5	0.2	0.4	0.5	0.5	0.6
M00166	0.5	0.5	0.5	0.5	0.5	0.5	0.3	0.5	0.5	0.5	0.5	0.5	0.5	0.3	0.5	0.5	0.3	0.8	0.8	0.8	0.5	0.5	0.5	0.5	0.5	0.5	0.5	0.0	0.5	0.0	0.5	0.5	0.5	0.8
M00167	0.4	0.4	0.1	0.1	0.1	0.4	0.1	0.4	0.4	0.4	0.4	0.4	0.0	0.1	0.1	0.1	0.1	0.1	0.1	0.0	0.0	0.4	0.4	0.6	0.4	0.1	0.4	0.3	0.4	0.3	0.3	0.4	0.4	0.6
M00168	0.0	0.0	0.0	0.0	0.0	0.0	0.0	0.0	0.0	0.0	0.0	0.0	0.0	0.0	0.0	0.0	0.0	0.0	0.5	0.5	0.0	0.0	0.0	0.5	0.0	0.0	0.0	0.5	0.0	0.5	0.0	0.0	0.0	0.5
M00169	0.0	0.0	0.0	0.0	0.0	0.0	0.0	0.0	0.0	0.0	0.0	0.0	0.0	0.0	0.0	0.0	0.0	0.5	0.0	0.0	0.0	0.5	0.5	0.5	0.5	0.5	0.5	0.5	0.5	0.5	0.5	0.5	0.5	0.5
M00170	0.0	0.0	0.0	0.0	0.0	0.0	0.0	0.0	0.0	0.0	0.0	0.0	0.0	0.0	0.0	0.0	0.0	0.0	0.3	0.3	0.0	0.0	0.0	0.0	0.0	0.0	0.0	0.0	0.0	0.0	0.0	0.0	0.0	0.0
M00171	0.0	0.0	0.0	0.0	0.0	0.0	0.0	0.0	0.0	0.0	0.0	0.0	0.0	0.0	0.0	0.0	0.0	0.1	0.1	0.1	0.0	0.1	0.1	0.1	0.1	0.1	0.1	0.1	0.1	0.1	0.1	0.1	0.1	0.1
M00172	0.0	0.0	0.0	0.0	0.0	0.0	0.0	0.0	0.0	0.0	0.0	0.0	0.0	0.0	0.0	0.0	0.0	0.3	0.3	0.3	0.0	0.3	0.3	0.3	0.3	0.3	0.3	0.3	0.3	0.3	0.3	0.3	0.3	0.3
M00173	0.3	0.2	0.3	0.3	0.3	0.3	0.2	0.3	0.3	0.3	0.3	0.3	0.2	0.2	0.2	0.2	0.2	0.4	0.4	0.4	0.3	0.2	0.3	0.5	0.4	0.2	0.3	0.4	0.4	0.4	0.2	0.4	0.3	0.5
M00174	0.0	0.0	0.0	0.0	0.0	0.0	0.0	0.0	0.0	0.0	0.0	0.0	0.0	0.0	0.0	0.0	0.0	0.0	0.0	0.0	0.0	0.0	0.0	0.0	0.0	0.0	0.0	0.0	0.0	0.0	0.0	0.0	0.0	0.0
M00175	0.3	0.3	0.0	0.0	0.3	0.3	0.3	0.3	0.7	0.7	0.7	0.7	0.3	0.3	0.3	0.0	0.0	0.3	0.0	0.3	0.0	0.7	0.3	0.7	1.0	0.0	0.0	1.0	0.0	0.3	0.0	0.0	1.0	1.0
M00176	0.0	0.0	0.0	0.0	0.3	0.3	0.0	0.2	0.2	0.0	0.2	0.2	0.5	0.2	0.2	0.0	0.5	0.0	0.0	0.0	0.3	0.0	0.0	0.5	0.0	0.0	0.0	0.0	0.0	0.0	0.0	0.0	0.0	0.3
M00307	1.0	1.0	1.0	1.0	1.0	1.0	0.5	1.0	1.0	1.0	1.0	1.0	1.0	0.5	1.0	1.0	0.8	1.0	1.0	1.0	1.0	1.0	1.0	1.0	1.0	1.0	1.0	1.0	1.0	1.0	1.0	1.0	1.0	1.0
M00308	0.4	0.4	0.4	0.4	0.4	0.4	0.2	0.4	0.4	0.4	0.4	0.4	0.4	0.2	0.4	0.4	0.2	0.4	0.4	0.4	0.4	0.2	0.2	0.3	0.2	0.2	0.2	0.0	0.2	0.0	0.2	0.0	0.2	0.2
M00309	0.3	0.3	0.3	0.3	0.3	0.3	0.3	0.3	0.3	0.3	0.3	0.3	0.3	0.3	0.3	0.3	0.3	0.3	0.3	0.3	0.3	0.3	0.3	0.3	0.3	0.3	0.3	0.3	0.3	0.3	0.3	0.3	0.3	0.3
M00338	0.0	0.0	0.0	0.0	0.0	0.0	0.0	0.0	0.0	0.0	0.0	0.0	0.0	0.0	0.0	0.0	0.0	0.0	0.0	0.0	0.0	0.0	0.0	0.0	0.0	0.0	0.0	0.0	0.0	0.0	0.0	0.0	0.0	0.0
M00344	0.3	0.3	0.0	0.0	0.0	0.3	0.0	0.3	0.3	0.3	0.3	0.3	0.0	0.0	0.0	0.0	0.0	0.0	0.0	0.0	0.0	0.3	0.3	0.5	0.3	0.0	0.3	0.3	0.3	0.3	0.3	0.5	0.3	0.5
M00345	1.0	0.7	0.7	0.7	0.3	1.0	0.3	1.0	1.0	1.0	1.0	1.0	0.5	0.3	0.3	0.3	0.0	0.8	0.5	0.5	0.3	0.8	0.8	0.8	0.8	0.5	0.7	0.5	0.8	0.5	0.3	0.8	0.8	0.8
M00346	0.2	0.1	0.2	0.2	0.2	0.2	0.2	0.3	0.3	0.3	0.3	0.2	0.2	0.1	0.1	0.1	0.2	0.2	0.3	0.3	0.2	0.2	0.2	0.3	0.2	0.1	0.2	0.3	0.2	0.3	0.2	0.2	0.2	0.3
M00356	0.6	0.6	0.4	0.6	0.4	0.3	0.2	0.6	0.3	0.4	0.3	0.6	0.4	0.4	0.4	0.4	0.4	0.4	0.4	0.4	0.4	0.6	0.4	0.7	0.7	0.7	0.4	0.4	0.6	0.4	0.3	0.7	0.7	0.7
M00357	0.6	0.6	0.5	0.6	0.7	0.6	0.5	0.6	0.5	0.5	0.5	0.6	0.6	0.5	0.5	0.5	0.3	0.5	0.5	0.5	0.5	0.7	0.6	1.0	1.0	0.8	0.7	0.7	0.7	0.7	0.5	1.0	0.8	1.0
M00358	0.6	0.5	0.4	0.3	0.0	0.1	0.1	0.5	0.5	0.5	0.5	0.5	0.0	0.0	0.1	0.1	0.0	0.8	0.5	0.5	0.3	0.1	0.3	0.0	0.3	0.3	0.3	0.0	0.3	0.0	0.1	0.1	0.3	0.0
M00364	1.0	0.5	1.0	1.0	0.5	0.5	0.5	0.5	0.5	0.5	0.5	0.5	0.0	0.0	0.5	0.5	0.5	0.5	0.5	0.5	0.5	0.5	0.5	0.5	0.0	0.5	0.5	0.5	0.5	0.5	0.5	1.0	0.5	0.5
M00365	1.0	0.5	1.0	1.0	0.5	0.5	0.5	0.5	0.5	0.5	0.5	0.5	0.0	0.0	0.5	0.5	0.5	0.5	0.5	0.5	0.5	0.5	0.5	0.5	0.0	0.5	0.5	0.5	0.5	0.5	0.5	1.0	0.5	0.5
M00366	0.3	0.0	0.3	0.3	0.0	0.0	0.0	0.0	0.0	0.0	0.0	0.0	0.0	0.0	0.3	0.3	0.3	0.3	0.3	0.3	0.3	0.3	0.3	0.3	0.0	0.3	0.3	0.3	0.3	0.3	0.3	0.3	0.3	0.0
M00367	0.3	0.0	0.3	0.3	0.0	0.0	0.0	0.0	0.0	0.0	0.0	0.0	0.0	0.0	0.3	0.3	0.3	0.3	0.3	0.3	0.3	0.3	0.3	0.3	0.0	0.3	0.3	0.3	0.3	0.3	0.3	0.3	0.3	0.0
M00368	0.3	0.0	0.0	0.3	0.3	0.3	0.3	0.3	0.3	0.3	0.3	0.3	0.0	0.3	0.3	0.3	0.3	0.3	0.3	0.3	0.0	0.3	0.3	0.3	0.3	0.3	0.3	0.3	0.3	0.3	0.0	0.3	0.3	0.3
M00373	0.1	0.1	0.1	0.1	0.1	0.1	0.1	0.1	0.1	0.1	0.1	0.1	0.1	0.1	0.1	0.1	0.1	0.1	0.2	0.1	0.1	0.1	0.1	0.1	0.1	0.1	0.1	0.1	0.1	0.1	0.1	0.1	0.1	0.1

M00374	Dicarboxylate-hydroxybutyrate cycle	0.3	0.3	0.3	0.4	0.3	0.3	0.4	0.4	0.3	0.3	0.2	0.3	0.3	0.3	0.2	0.3	0.3	0.3	0.2	0.3	0.3	0.2	0.3	0.3	0.2	0.2	0.3	0.2	0.3	0.3	0.3	0.3	0.3	0.3	0.3	0.3	0.3	0.3	0.3	0.3	0.3	0.3	0.3	0.3	0.2	0.2	0.4	0.4	0.3	0.2	0.3	0.3	0.3	0.2	0.2	0.3	0.2	0.2	0.2	0.3	0.2	0.3	0.2	0.2	0.2	0.4
M00375	Hydroxypropionate-hydroxybutylate cycle	0.1	0.1	0.1	0.1	0.1	0.1	0.1	0.1	0.1	0.1	0.1	0.1	0.1	0.1	0.0	0.1	0.1	0.1	0.0	0.1	0.1	0.1	0.1	0.1	0.1	0.1	0.1	0.1	0.1	0.1	0.1	0.1	0.1	0.1	0.1	0.1	0.1	0.1	0.1	0.1	0.1	0.1	0.1	0.1	0.1	0.1	0.3	0.2	0.1	0.1	0.1	0.1	0.1	0.1	0.1	0.1	0.1	0.1	0.1	0.1	0.1	0.1	0.1	0.1	0.1	0.1
M00376	3-Hydroxypropionate bi-cycle	0.0	0.0	0.0	0.0	0.0	0.0	0.0	0.0	0.0	0.0	0.0	0.0	0.0	0.0	0.0	0.0	0.0	0.0	0.0	0.0	0.0	0.0	0.0	0.0	0.0	0.0	0.0	0.0	0.0	0.0	0.0	0.0	0.0	0.0	0.0	0.0	0.0	0.0	0.0	0.0	0.0	0.0	0.0	0.0	0.0	0.0	0.0	0.0	0.1	0.0	0.1	0.1	0.0	0.0	0.0	0.0	0.0	0.0	0.0	0.0	0.0	0.0	0.0	0.1	0.0	0.1
M00377	Reductive acetyl-CoA pathway (Wood-Ljungdahl pathway)	0.1	0.1	0.1	0.1	0.1	0.1	0.1	0.1	0.1	0.1	0.1	0.1	0.1	0.1	0.0	0.1	0.4	0.2	0.0	0.1	0.2	0.1	0.1	0.1	0.1	0.1	0.1	0.0	0.1	0.1	0.0	0.1	0.1	0.1	0.1	0.1	0.1	0.1	0.1	0.1	0.2	0.2	0.2	0.2	0.2	0.2	0.4	0.1	0.0	0.4	0.4	0.4	0.1	0.2	0.2	0.4	0.2	0.1	0.2	0.2	0.2	0.2	0.1	0.1	0.1	0.5
M00378	F420 biosynthesis. archaea	0.8	0.8	0.8	0.8	0.8	0.8	0.8	0.1	0.6	0.8	0.6	0.4	0.9	0.5	0.5	0.6	0.8	0.8	0.8	0.8	0.8	0.3	0.8	0.8	0.8	0.8	0.3	0.8	0.8	0.8	0.3	0.8	0.9	0.5	0.8	0.8	0.9	0.9	0.9	0.6	0.6	0.6	0.6	0.6	0.6	0.9	0.5	0.6	0.4	0.9	0.8	0.8	0.5	0.9	1.0	0.8	0.9	1.0	0.8	1.0	1.0	1.0	0.8	0.6	0.9	0.9
M00422	Acetyl-CoA pathway. CO2 => acetyl-CoA	0.8	1.0	0.8	1.0	0.8	0.6	0.8	0.8	1.0	1.0	1.0	0.8	1.0	0.8	0.0	0.5	0.8	1.0	0.0	1.0	1.0	0.8	1.0	1.0	1.0	0.8	0.8	0.7	0.6	0.6	0.3	1.0	1.0	1.0	1.0	1.0	1.0	0.8	1.0	0.8	1.0	1.0	1.0	1.0	1.0	0.5	1.0	1.0	0.5	1.0	1.0	1.0	1.0	1.0	1.0	1.0	1.0	1.0	1.0	1.0	1.0	1.0	0.8	1.0	1.0	1.0
M00432	Leucine biosynthesis. 2-oxoisovalerate => 2-oxoisocaproate	0.3	1.0	0.7	0.8	1.0	1.0	0.7	0.7	0.2	0.7	0.5	0.7	1.0	0.7	0.3	1.0	1.0	0.7	0.3	0.7	1.0	0.8	1.0	0.8	0.8	0.7	0.3	0.7	0.5	0.7	0.3	1.0	1.0	0.8	0.8	1.0	0.5	0.8	0.8	1.0	1.0	1.0	1.0	1.0	0.8	0.8	0.2	0.2	0.5	0.7	0.7	0.7	1.0	0.7	1.0	0.3	1.0	1.0	1.0	1.0	0.7	1.0	0.3	0.3	1.0	0.3
M00433	Lysine biosynthesis. 2-oxoglutarate => 2-oxoadipate	0.0	0.0	0.0	0.0	0.0	0.0	0.0	0.0	0.0	0.0	0.0	0.0	0.0	0.0	0.0	0.0	0.0	0.0	0.0	0.0	0.0	0.0	0.0	0.0	0.0	0.0	0.0	0.0	0.0	0.0	0.0	0.0	0.0	0.0	0.0	0.0	0.0	0.0	0.0	0.0	0.0	0.0	0.0	0.0	0.0	0.0	0.0	0.0	0.0	0.0	0.0	0.0	0.0	0.2	0.0	0.3	0.2	0.2	0.2	0.2	0.2	0.2	0.2	0.0	0.3	0.3
M00525	Lysine biosynthesis. acetyl-DAP pathway. aspartate => lysine	0.4	0.7	0.3	0.7	0.6	0.7	0.6	0.4	0.4	0.7	0.4	0.6	0.7	0.6	0.2	0.7	0.7	0.7	0.6	0.6	0.7	0.7	0.7	0.6	0.6	0.2	0.2	0.6	0.4	0.4	0.4	0.6	0.7	0.6	0.6	0.7	0.3	0.4	0.7	0.7	0.7	0.7	0.7	0.7	0.7	0.3	0.6	0.6	0.3	0.7	0.6	0.6	0.6	0.4	0.6	0.6	0.7	0.7	0.4	0.7	0.7	0.6	0.6	0.7	0.6	0.7
M00526	Lysine biosynthesis. DAP dehydrogenase pathway. aspartate => lysine	0.5	0.8	0.5	0.8	0.7	0.8	0.7	0.5	0.7	0.8	0.7	0.7	0.8	0.7	0.3	0.8	0.8	0.8	0.7	0.7	1.0	0.8	1.0	0.8	0.8	0.3	0.2	0.8	0.8	0.7	0.7	0.8	0.8	0.7	0.7	0.8	0.3	0.5	0.8	0.8	0.8	0.8	0.8	0.8	0.8	0.3	0.7	0.7	0.5	0.8	0.7	0.7	0.7	0.7	0.7	0.7	0.8	0.8	0.5	0.8	0.8	0.7	0.8	0.8	0.8	0.8
M00527	Lysine biosynthesis. DAP aminotransferase pathway. aspartate => lysine	0.7	1.0	0.6	1.0	0.9	1.0	0.9	0.7	0.7	0.9	0.6	0.9	1.0	0.7	0.3	1.0	1.0	1.0	0.9	0.9	1.0	1.0	1.0	0.9	0.9	0.3	0.4	0.9	0.6	0.7	0.7	0.9	1.0	0.7	0.9	1.0	0.6	0.6	1.0	1.0	1.0	1.0	1.0	1.0	1.0	0.6	0.9	0.7	0.4	1.0	0.9	0.9	0.9	0.6	0.9	0.9	1.0	0.9	0.6	0.9	0.9	0.9	0.7	1.0	0.9	1.0
M00528	Nitrification. ammonia => nitrite	0.0	0.0	0.0	0.0	0.0	0.0	0.0	0.0	0.0	0.0	0.0	0.0	0.0	0.0	0.0	0.0	0.0	0.0	0.0	0.0	0.0	0.0	0.0	0.5	0.0	0.0	0.0	0.0	0.0	0.0	0.0	0.0	0.0	0.0	0.0	0.0	0.0	0.0	0.0	0.0	0.0	0.0	0.0	0.0	0.0	0.0	0.0	0.0	0.0	0.5	0.5	0.5	0.0	0.5	0.5	0.0	0.5	0.0	0.5	0.5	0.5	0.5	0.0	0.0	0.0	0.0
M00529	Denitrification. nitrate => nitrogen	0.0	0.0	0.0	0.0	0.0	0.0	0.0	0.0	0.0	0.0	0.0	0.0	0.0	0.0	0.0	0.0	0.0	0.0	0.0	0.0	0.0	0.0	0.0	0.0	0.0	0.0	0.0	0.0	0.0	0.0	0.0	0.0	0.0	0.0	0.0	0.0	0.0	0.0	0.0	0.0	0.0	0.0	0.0	0.0	0.0	0.0	0.0	0.0	0.0	0.0	0.0	0.0	0.0	0.0	0.0	0.0	0.0	0.0	0.1	0.0	0.0	0.0	0.0	0.0	0.0	0.0
M00530	Dissimilatory nitrate reduction. nitrate => ammonia	0.0	0.0	0.0	0.0	0.0	0.0	0.0	0.0	0.0	0.0	0.0	0.0	0.0	0.0	0.0	0.0	0.0	0.0	0.0	0.0	0.0	0.0	0.0	0.0	0.0	0.0	0.0	0.0	0.0	0.0	0.0	0.0	0.0	0.0	0.0	0.0	0.0	0.0	0.0	0.0	0.0	0.0	0.0	0.0	0.0	0.0	0.0	0.0	0.0	0.0	0.0	0.0	0.0	0.0	0.0	0.0	0.0	0.0	0.3	0.0	0.0	0.0	0.0	0.0	0.0	0.0
M00531	Assimilatory nitrate reduction. nitrate => ammonia	0.0	0.0	0.0	0.0	0.0	0.0	0.0	0.0	0.0	0.0	0.0	0.0	0.0	0.0	0.0	0.0	0.0	0.0	0.0	0.0	0.0	0.0	0.0	0.0	0.0	0.0	0.0	0.0	0.0	0.0	0.0	0.0	0.0	0.0	0.0	0.0	0.0	0.0	0.0	0.0	0.0	0.0	0.0	0.0	0.0	0.0	0.0	0.0	0.0	0.0	0.0	0.0	0.0	0.0	0.0	0.0	0.0	0.0	0.0	0.0	0.0	0.0	0.0	0.0	0.0	0.0
M00532	Photorespiration	0.2	0.2	0.2	0.2	0.2	0.2	0.1	0.1	0.1	0.2	0.1	0.2	0.1	0.1	0.1	0.1	0.1	0.1	0.0	0.1	0.1	0.1	0.2	0.2	0.2	0.1	0.2	0.2	0.1	0.1	0.1	0.2	0.1	0.1	0.1	0.1	0.1	0.1	0.1	0.1	0.1	0.1	0.1	0.1	0.1	0.0	0.0	0.0	0.1	0.2	0.2	0.2	0.1	0.2	0.2	0.1	0.2	0.0	0.1	0.2	0.2	0.2	0.2	0.2	0.1	0.3
M00535	Isoleucine biosynthesis. pyruvate => 2-oxobutanoate	0.0	0.7	0.3	0.8	1.0	1.0	0.7	0.7	0.2	0.3	0.5	0.3	1.0	0.7	0.0	1.0	1.0	0.7	0.3	0.7	1.0	0.8	1.0	0.8	0.5	0.7	0.3	0.7	0.5	0.7	0.3	1.0	1.0	0.5	0.8	1.0	0.5	0.8	0.8	1.0	1.0	1.0	0.7	0.7	0.8	0.8	0.2	0.2	0.5	0.3	0.3	0.3	1.0	0.7	1.0	0.7	1.0	0.7	1.0	1.0	0.7	1.0	0.7	0.3	1.0	0.7
M00549	Nucleotide sugar biosynthesis. glucose => UDP-glucose	0.0	0.0	0.0	0.0	0.3	0.0	0.0	0.0	0.0	0.3	0.0	0.0	0.0	0.0	0.3	0.3	0.3	0.3	0.3	0.3	0.3	0.3	0.7	0.0	0.3	0.7	0.0	0.3	0.3	0.3	0.3	0.3	0.0	0.0	0.0	0.0	0.3	0.0	0.0	0.3	0.0	0.0	0.0	0.0	0.0	0.0	0.0	0.0	0.0	0.3	0.0	0.0	0.0	0.3	0.3	0.0	0.3	0.0	0.3	0.3	0.0	0.3	0.3	0.0	0.3	0.0
M00550	Ascorbate degradation. ascorbate => D-xylulose-5P	0.0	0.0	0.0	0.0	0.0	0.0	0.0	0.0	0.0	0.0	0.0	0.0	0.0	0.0	0.0	0.0	0.0	0.0	0.0	0.0	0.0	0.0	0.2	0.0	0.0	0.0	0.0	0.0	0.0	0.0	0.0	0.0	0.0	0.0	0.0	0.0	0.0	0.0	0.0	0.0	0.0	0.0	0.0	0.0	0.0	0.0	0.0	0.0	0.0	0.0	0.0	0.0	0.0	0.0	0.0	0.0	0.0	0.0	0.0	0.0	0.0	0.0	0.0	0.0	0.0	0.0
M00552	D-galactonate degradation. De Ley-Doudoroff pathway. D-galactonate => glycerate-3P	0.0	0.4	0.0	0.4	0.4	0.4	0.4	0.0	0.0	0.4	0.4	0.4	0.4	0.4	0.2	0.4	0.4	0.4	0.0	0.2	0.4	0.4	0.4	0.4	0.4	0.2	0.4	0.4	0.4	0.4	0.0	0.4	0.4	0.4	0.4	0.4	0.4	0.4	0.2	0.4	0.4	0.4	0.4	0.4	0.4	0.2	0.4	0.4	0.2	0.4	0.4	0.4	0.4	0.2	0.2	0.2	0.2	0.2	0.2	0.0	0.2	0.0	0.2	0.0	0.2	0.2
M00554	Nucleotide sugar biosynthesis. galactose => UDP-galactose	0.0	0.0	0.0	0.0	0.0	0.0	0.0	0.0	0.0	0.0	0.0	0.5	0.0	0.0	0.0	0.0	0.0	0.0	0.0	0.0	0.0	0.0	0.5	0.0	0.0	0.0	0.0	0.0	0.0	0.0	0.0	0.0	0.0	0.0	0.0	0.0	0.0	0.0	0.0	0.0	0.0	0.0	0.0	0.0	0.0	0.0	0.0	0.0	0.5	0.5	0.5	0.5	0.0	0.5	0.0	0.0	0.0	0.0	0.0	0.5	0.5	0.5	0.0	0.0	0.5	0.0
M00563	Methanogenesis. methylamine/dimethylamine/trimethylamine => methane	0.1	0.3	0.3	0.3	0.3	0.3	0.4	0.3	0.4	0.3	0.3	0.3	0.3	0.3	0.2	0.3	0.3	0.4	0.2	0.3	0.4	0.3	0.4	0.3	0.2	0.3	0.3	0.3	0.2	0.2	0.2	0.3	0.4	0.4	0.3	0.4	0.3	0.3	0.2	0.4	0.3	0.3	0.3	0.4	0.3	0.3	0.6	0.6	0.3	0.3	0.3	0.3	0.3	0.4	0.3	0.5	0.5	0.5	0.3	0.3	0.4	0.3	0.3	0.5	0.5	0.5
M00567	Methanogenesis. CO2 => methane	0.5	0.7	0.7	0.6	0.7	0.5	0.8	0.7	0.8	0.7	0.4	0.7	0.7	0.6	0.4	0.7	0.7	0.7	0.6	0.7	0.8	0.6	0.8	0.7	0.3	0.6	0.7	0.5	0.6	0.6	0.5	0.7	0.8	0.6	0.7	0.8	0.6	0.6	0.6	0.8	0.7	0.7	0.6	0.7	0.7	0.7	0.8	0.5	0.6	0.7	0.7	0.7	0.4	0.8	0.9	1.0	1.0	1.0	0.9	0.8	1.0	0.8	0.6	1.0	1.0	1.0
M00568	Catechol ortho-cleavage. catechol => 3-oxoadipate	0.0	0.0	0.3	0.3	0.0	0.0	0.0	0.0	0.0	0.0	0.0	0.0	0.0	0.0	0.0	0.0	0.0	0.0	0.0	0.0	0.0	0.0	0.0	0.3	0.3	0.0	0.0	0.0	0.0	0.0	0.0	0.0	0.0	0.0	0.0	0.0	0.0	0.0	0.0	0.0	0.0	0.0	0.0	0.0	0.0	0.0	0.0	0.3	0.0	0.0	0.0	0.0	0.0	0.0	0.0	0.0	0.0	0.0	0.0	0.0	0.0	0.0	0.0	0.0	0.0	0.3
M00569	Catechol meta-cleavage. catechol => acetyl-CoA / 4-methylcatechol => propanoyl-CoA	0.0	0.0	0.0	0.0	0.0	0.0	0.0	0.0	0.0	0.0	0.0	0.0	0.0	0.0	0.0	0.0	0.1	0.0	0.1	0.0	0.1	0.0	0.0	0.0	0.0	0.0	0.0	0.0	0.0	0.0	0.0	0.0	0.0	0.0	0.0	0.0	0.0	0.0	0.0	0.0	0.0	0.0	0.0	0.0	0.0	0.0	0.0	0.0	0.0	0.0	0.0	0.0	0.0	0.0	0.1	0.0	0.0	0.0	0.0	0.0	0.0	0.0	0.0	0.0	0.0	0.1
M00570	Isoleucine biosynthesis. threonine => 2-oxobutanoate => isoleucine	0.6	0.8	0.4	0.6	0.6	0.3	0.8	0.3	0.6	0.8	0.4	0.8	0.8	0.8	0.0	0.8	0.6	0.8	0.4	0.8	0.8	0.8	0.8	0.5	0.4	0.5	0.7	0.6	0.8	0.6	0.6	0.8	0.8	0.8	0.8	0.8	0.8	0.8	0.8	0.8	0.8	0.8	0.8	0.8	0.6	0.6	0.2	0.2	0.0	0.8	0.8	0.8	0.8	0.8	0.8	0.8	0.8	0.8	0.8	0.8	0.8	0.8	0.8	0.6	0.8	0.8
M00573	Biotin biosynthesis. BioI pathway. long-chain-acyl-ACP => pimeloyl-ACP => biotin	0.0	0.0	0.0	0.0	0.0	0.0	0.0	0.0	0.0	0.0	0.0	0.0	0.0	0.0	0.0	0.0	0.0	0.0	0.0	0.0	0.0	0.0	0.0	0.0	0.0	0.0	0.0	0.0	0.0	0.0	0.0	0.0	0.0	0.0	0.0	0.0	0.0	0.0	0.0	0.0	0.0	0.0	0.0	0.0	0.0	0.0	0.0	0.0	0.0	0.0	0.0	0.0	0.0	0.0	0.0	0.0	0.0	0.0	0.0	0.6	0.0	0.6	0.0	0.0	0.0	0.0
M00577	Biotin biosynthesis. BioW pathway. pimelate => pimeloyl-CoA => biotin	0.0	0.0	0.0	0.0	0.0	0.0	0.0	0.0	0.0	0.0	0.0	0.0	0.0	0.0	0.0	0.0	0.0	0.0	0.0	0.0	0.0	0.0	0.0	0.0	0.0	0.0	0.0	0.0	0.0	0.0	0.0	0.0	0.0	0.0	0.0	0.0	0.0	0.0	0.0	0.0	0.0	0.0	0.0	0.0	0.0	0.0	0.0	0.0	0.0	0.0	0.0	0.0	0.0	0.0	0.0	0.0	0.0	0.0	0.0	0.8	0.0	0.8	0.0	0.0	0.0	0.0
M00580	Pentose phosphate pathway. archaea. fructose 6P => ribose 5P	1.0	1.0	0.7	1.0	1.0	0.7	1.0	1.0	1.0	1.0	0.7	1.0	1.0	0.5	0.0	0.7	1.0	1.0	1.0	0.5	1.0	0.3	0.7	1.0	0.7	0.5	0.3	0.7	0.7	1.0	0.5	1.0	1.0	1.0	1.0	1.0	1.0	1.0	1.0	1.0	1.0	1.0	1.0	1.0	0.7	0.7	1.0	1.0	0.5	1.0	1.0	0.7	0.5	1.0	1.0	1.0	1.0	1.0	1.0	1.0	1.0	1.0	0.0	0.7	1.0	1.0
M00595	Thiosulfate oxidation by SOX complex. thiosulfate => sulfate	0.0	0.0	0.0	0.0	0.0	0.0	0.0	0.0	0.0	0.0	0.0	0.0	0.0	0.0	0.0	0.0	0.0	0.0	0.0	0.0	0.0	0.0	0.0	0.0	0.0	0.0	0.0	0.0	0.0	0.0	0.0	0.0	0.0	0.0	0.0	0.0	0.0	0.0	0.0	0.0	0.0	0.0	0.0	0.0	0.0	0.0	0.0	0.0	0.0	0.0	0.0	0.0	0.0	0.0	0.0	0.0	0.0	0.0	0.0	0.0	0.0	0.0	0.0	0.0	0.0	0.0
M00596	Dissimilatory sulfate reduction. sulfate => H2S	0.0	0.0	0.0	0.0	0.0	0.0	0.3	0.0	0.0	0.0	0.0	0.0	0.0	0.0	0.0	0.0	0.0	0.0	0.0	0.0	0.0	0.0	0.0	0.0	0.0	0.0	0.0	0.0	0.0	0.3	0.0	0.0	0.0	0.0	0.0	0.0	0.0	0.0	0.0	0.0	0.0	0.0	0.0	0.0	0.0	0.0	0.3	0.0	0.0	0.0	0.0	0.0	0.0	0.0	0.0	0.0	0.0	0.0	0.0	0.0	0.0	0.0	0.0	0.0	0.0	0.0
M00608	2-Oxocarboxylic acid chain extension. 2-oxoglutarate => 2-oxoadipate => 2-oxopimelate	0.0	0.0	0.0	0.0	0.0	0.0	0.0	0.0	0.0	0.0	0.0	0.0	0.0	0.0	0.0	0.0	0.0	0.0	0.0	0.0	0.0	0.0	0.0	0.0	0.0	0.0	0.0	0.0	0.0	0.0	0.0	0.0	0.0	0.0	0.0	0.0	0.0	0.0	0.0	0.0	0.0	0.0	0.0	0.0	0.0	0.0	0.0	0.0	0.0	0.0	0.0	0.0	0.0	0.5	0.3	0.7	0.5	0.2	0.5	0.5	0.5	0.5	0.5	0.0	0.7	1.0
M00609	Cysteine biosynthesis. methionine => cysteine	0.2	0.2	0.2	0.2	0.2	0.2	0.2	0.2	0.0	0.2	0.0	0.2	0.2	0.2	0.0	0.2	0.2	0.2	0.2	0.2	0.2	0.2	0.2	0.2	0.2	0.2	0.2	0.2	0.3	0.2	0.2	0.2	0.2	0.0	0.0	0.2	0.2	0.2	0.2	0.2	0.2	0.2	0.2	0.2	0.0	0.2	0.2	0.2	0.2	0.3	0.2	0.2	0.0	0.2	0.2	0.2	0.3	0.2	0.2	0.3	0.2	0.3	0.0	0.2	0.2	0.2
M00611	Oxygenic photosynthesis in plants and cyanobacteria	0.1	0.1	0.1	0.1	0.1	0.1	0.1	0.1	0.1	0.1	0.1	0.2	0.1	0.1	0.2	0.1	0.1	0.1	0.1	0.1	0.2	0.1	0.2	0.2	0.2	0.1	0.2	0.3	0.2	0.2	0.0	0.1	0.2	0.2	0.1	0.1	0.1	0.2	0.1	0.2	0.2	0.2	0.2	0.2	0.1	0.1	0.1	0.1	0.1	0.2	0.2	0.1	0.1	0.2	0.2	0.3	0.2	0.1	0.2	0.1	0.2	0.1	0.2	0.2	0.2	0.3
M00612	Anoxygenic photosynthesis in purple bacteria	0.1	0.1	0.1	0.1	0.1	0.1	0.1	0.1	0.1	0.1	0.1	0.2	0.1	0.1	0.2	0.1	0.1	0.1	0.1	0.1	0.2	0.1	0.2	0.2	0.2	0.1	0.2	0.3	0.2	0.2	0.0	0.1	0.2	0.2	0.1	0.1	0.1	0.2	0.1	0.2	0.2	0.2	0.2	0.2	0.1	0.1	0.1	0.1	0.1	0.2	0.2	0.1	0.1	0.2	0.2	0.3	0.2	0.1	0.2	0.1	0.2	0.1	0.2	0.2	0.2	0.3
M00614	Anoxygenic photosynthesis in green sulfur bacteria	0.1	0.1	0.1	0.2	0.2	0.2	0.2	0.2	0.1	0.2	0.1	0.2	0.1	0.1	0.1	0.1	0.2	0.2	0.2	0.1	0.3	0.1	0.2	0.2	0.2	0.1	0.2	0.1	0.2	0.2	0.1	0.2	0.1	0.1	0.1	0.1	0.1	0.1	0.1	0.1	0.1	0.1	0.1	0.1	0.1	0.1	0.1	0.1	0.1	0.2	0.2	0.2	0.1	0.1	0.1	0.2	0.2	0.1	0.2	0.2	0.2	0.2	0.1	0.2	0.1	0.3
M00615	Nitrate assimilation	0.0	0.0	0.0	0.0	0.0	0.0	0.0	0.0	0.0	0.0	0.0	0.0	0.0	0.0	0.0	0.0	0.0	0.0	0.0	0.0	0.0	0.0	0.0	0.0	0.0	0.0	0.0	0.0	0.0	0.0	0.0	0.0	0.0	0.0	0.0	0.0	0.0	0.0	0.0	0.0	0.0	0.0	0.0	0.0	0.0	0.0	0.0	0.0	0.0	0.0	0.0	0.0	0.0	0.0	0.0	0.0	0.0	0.0	0.0	0.0	0.0	0.0	0.0	0.0	0.0	0.0
M00616	Sulfate-sulfur assimilation	0.1	0.1	0.3	0.1	0.3	0.3	0.1	0.0	0.0	0.0	0.0	0.0	0.2	0.2	0.0	0.3	0.1	0.0	0.0	0.0	0.0	0.0	0.3	0.0	0.0	0.1	0.0	0.0	0.0	0.2	0.0	0.0	0.0	0.0	0.0	0.0	0.2	0.2	0.0	0.1	0.1	0.0	0.1	0.1	0.3	0.1	0.1	0.0	0.3	0.0	0.0	0.0	0.2	0.0	0.0	0.3	0.0	0.0	0.0	0.0	0.0	0.0	0.0	0.0	0.0	0.2
M00617	Methanogen	0.5	0.7	0.7	0.6	0.7	0.5	0.8	0.8	0.8	0.8	0.6	0.7	0.8	0.6	0.4	0.7	0.7	0.8	0.6	0.7	0.8	0.6	0.8	0.8	0.5	0.6	0.7	0.5	0.6	0.6	0.5	0.7	0.8	0.6	0.7	0.8	0.7	0.6	0.6	0.8	0.7	0.7	0.6	0.7	0.7	0.7	0.8	0.6	0.6	0.7	0.7	0.7	0.5	0.8	0.9	1.0	1.0	1.0	0.9	0.8	1.0	0.8	0.6	1.0	1.0	1.0
M00618	Acetogen	0.1	0.1	0.1	0.1	0.0	0.0	0.0	0.1	0.1	0.1	0.1	0.1	0.1	0.1	0.0	0.1	0.2	0.1	0.0	0.1	0.1	0.0	0.1	0.1	0.1	0.1	0.1	0.0	0.1	0.1	0.0	0.1	0.1	0.1	0.1	0.1	0.1	0.1	0.1	0.1	0.1	0.1	0.1	0.1	0.1	0.1	0.2	0.1	0.0	0.2	0.2	0.2	0.1	0.1	0.1	0.2	0.1	0.1	0.1	0.1	0.1	0.1	0.0	0.1	0.1	0.3
M00620	Incomplete reductive citrate cycle. acetyl-CoA => oxoglutarate	0.2	0.1	0.3	0.3	0.3	0.2	0.3	0.2	0.3	0.3	0.1	0.3	0.3	0.2	0.2	0.3	0.3	0.2	0.2	0.1	0.4	0.1	0.3	0.3	0.2	0.1	0.3	0.2	0.3	0.2	0.2	0.3	0.3	0.2	0.3	0.3	0.3	0.3	0.2	0.3	0.3	0.3	0.3	0.3	0.1	0.1	0.2	0.2	0.2	0.3	0.2	0.2	0.3	0.3	0.3	0.6	0.4	0.3	0.2	0.6	0.4	0.5	0.2	0.3	0.4	0.7
M00632	Galactose degradation. Leloir pathway. galactose => alpha-D-glucose-1P	0.3	0.3	0.3	0.3	0.3	0.0	0.3	0.3	0.3	0.3	0.3	0.5	0.3	0.0	0.3	0.3	0.3	0.0	0.0	0.3	0.3	0.0	0.5	0.3	0.3	0.3	0.3	0.3	0.3	0.3	0.3	0.3	0.3	0.3	0.3	0.0	0.0	0.3	0.3	0.3	0.3	0.3	0.3	0.3	0.0	0.3	0.0	0.0	0.3	0.5	0.5	0.3	0.0	0.5	0.3	0.3	0.3	0.3	0.3	0.5	0.5	0.5	0.3	0.0	0.5	0.3
M00740	Methylaspartate cycle	0.0	0.0	0.0	0.0	0.1	0.1	0.1	0.1	0.0	0.1	0.1	0.1	0.0	0.0	0.0	0.0	0.1	0.1	0.1	0.0	0.1	0.0	0.1	0.1	0.1	0.1	0.1	0.0	0.1	0.1	0.0	0.1	0.0	0.0	0.0	0.0	0.0	0.0	0.0	0.0	0.0	0.0	0.0	0.0	0.0	0.0	0.0	0.0	0.0	0.1	0.1	0.1	0.0	0.1	0.0	0.1	0.1	0.1	0.2	0.1	0.2	0.1	0.0	0.1	0.0	0.2
M00741	Propanoyl-CoA metabolism. propanoyl-CoA => succinyl-CoA	0.0	0.0	0.0	0.0	0.0	0.0	0.0	0.0	0.0	0.0	0.0	0.0	0.0	0.0	0.0	0.0	0.0	0.0	0.0	0.0	0.0	0.0	0.0	0.0	0.0	0.0	0.0	0.0	0.0	0.0	0.0	0.0	0.0	0.0	0.0	0.0	0.0	0.0	0.0	0.0	0.0	0.0	0.0	0.0	0.0	0.0	0.2	0.2	0.3	0.0	0.3	0.3	0.0	0.0	0.0	0.0	0.0	0.0	0.0	0.0	0.0	0.0	0.0	0.0	0.0	0.3
M00793	dTDP-L-rhamnose biosynthesis	0.0	0.0	0.0	0.0	0.0	0.0	0.5	0.0	0.0	0.8	0.0	0.0	0.0	0.0	0.0	1.0	0.0	0.0	0.0	0.0	0.0	0.3	1.0	0.8	1.0	1.0	0.8	0.0	0.8	0.8	0.3	0.8	0.8	0.3	0.0	0.0	0.0	0.0	0.0	1.0	0.0	0.0	0.0	0.0	0.0	0.0	0.0	0.0	0.0	0.0	0.0	0.0	0.0	0.0	0.0	0.3	0.8	0.8	0.0	0.0	0.0	0.8	0.0	0.0	0.0	0.5
M00804	Complete nitrification. comammox. ammonia => nitrite => nitrate	0.0	0.0	0.0	0.0	0.0	0.0	0.0	0.0	0.0	0.0	0.0	0.0	0.0	0.0	0.0	0.0	0.0	0.0	0.0	0.0	0.0	0.0	0.0	0.3	0.0	0.0	0.0	0.0	0.0	0.0	0.0	0.0	0.0	0.0	0.0	0.0	0.0	0.0	0.0	0.0	0.0	0.0	0.0	0.0	0.0	0.0	0.0	0.0	0.0	0.3	0.3	0.3	0.0	0.3	0.3	0.0	0.3	0.0	0.3	0.3	0.3	0.3	0.0	0.0	0.0	0.0
M00835	Pyocyanine biosynthesis. chorismate => pyocyanine	0.0	0.0	0.0	0.0	0.0	0.0	0.0	0.0	0.0	0.0	0.0	0.0	0.0	0.0	0.0	0.0	0.0	0.0	0.0	0.0	0.0	0.0	0.1	0.0	0.0	0.0	0.0	0.0	0.1	0.1	0.0	0.0	0.0	0.0	0.0	0.0	0.0	0.0	0.0	0.0	0.0	0.0	0.0	0.0	0.0	0.0	0.0	0.0	0.0	0.0	0.0	0.0	0.0	0.1	0.1	0.0	0.1	0.0	0.0	0.0	0.0	0.0	0.0	0.0	0.0	0.1
M00836	Coenzyme F430 biosynthesis. sirohydrochlorin => coenzyme F430	0.5	0.5	0.5	0.8	0.8	0.6	0.9	0.3	0.5	0.6	0.4	0.9	0.6	0.5	0.6	0.9	0.6	0.6	0.6	0.6	0.9	0.4	0.4	0.4	0.0	0.6	0.6	0.6	0.6	0.5	0.5	0.9	0.6	0.9	0.4	0.6	0.3	0.6	0.6	0.9	0.6	0.6	0.6	0.6	0.3	0.5	0.4	0.0	0.0	0.4	0.4	0.4	0.3	0.4	1.0	0.9	1.0	1.0	1.0	0.9	1.0	0.6	0.8	0.5	0.9	1.0
M00842	Tetrahydrobiopterin biosynthesis. GTP => BH4	0.0	0.3	0.3	0.0	0.3	0.0	0.3	0.3	0.0	0.3	0.3	0.3	0.3	0.0	0.3	0.3	0.3	0.3	0.0	0.3	0.3	0.0	0.3	0.3	0.3	0.3	0.3	0.3	0.3	0.3	0.3	0.3	0.3	0.3	0.3	0.3	0.0	0.3	0.3	0.3	0.3	0.3	0.3	0.3	0.3	0.0	0.3	0.3	0.3	0.3	0.3	0.3	0.3	0.3	0.3	0.3	0.3	0.3	0.3	0.3	0.3	0.3	0.3	0.3	0.0	0.0
M00843	L-threo-Tetrahydrobiopterin biosynthesis. GTP => L-threo-BH4	0.0	0.3	0.3	0.0	0.3	0.0	0.3	0.3	0.0	0.3	0.3	0.3	0.3	0.0	0.3	0.3	0.3	0.3	0.0	0.3	0.3	0.0	0.3	0.3	0.3	0.3	0.3	0.3	0.3	0.3	0.3	0.3	0.3	0.3	0.3	0.3	0.0	0.3	0.3	0.3	0.3	0.3	0.3	0.3	0.3	0.0	0.3	0.3	0.3	0.3	0.3	0.3	0.3	0.3	0.3	0.3	0.3	0.3	0.3	0.3	0.3	0.3	0.3	0.3	0.0	0.0
M00844	Arginine biosynthesis. ornithine => arginine	0.3	1.0	1.0	1.0	1.0	1.0	1.0	1.0	0.7	0.7	0.7	0.7	1.0	1.0	0.0	1.0	0.7	1.0	0.0	1.0	1.0	1.0	1.0	1.0	1.0	0.7	0.3	1.0	0.7	1.0	0.3	1.0	1.0	0.7	1.0	1.0	0.3	1.0	0.7	1.0	1.0	1.0	1.0	1.0	0.7	0.7	1.0	0.7	0.7	1.0	1.0	1.0	0.7	1.0	1.0	1.0	1.0	1.0	0.7	1.0	1.0	1.0	0.7	0.3	1.0	1.0
M00845	Arginine biosynthesis. glutamate => acetylcitrulline => arginine	0.3	0.6	0.6	0.6	0.6	0.4	0.6	0.4	0.3	0.4	0.3	0.3	0.6	0.6	0.3	0.6	0.3	0.6	0.0	0.6	0.6	0.6	0.6	0.6	0.6	0.4	0.4	0.6	0.6	0.6	0.1	0.6	0.6	0.4	0.6	0.6	0.3	0.4	0.6	0.6	0.6	0.6	0.6	0.6	0.4	0.4	0.6	0.4	0.4	0.6	0.6	0.6	0.3	0.4	0.6	0.6	0.6	0.6	0.4	0.4	0.4	0.6	0.3	0.4	0.6	0.6
M00846	Siroheme biosynthesis. glutamyl-tRNA => siroheme	0.4	0.6	0.4	0.8	0.9	0.6	0.8	0.3	0.7	0.9	0.7	0.9	0.9	0.5	0.4	0.9	0.2	0.9	0.9	0.9	0.9	0.6	0.9	0.9	0.8	0.6	0.6	0.8	0.6	0.8	0.7	0.8	0.9	0.4	0.8	0.9	0.9	0.7	0.9	0.8	0.9	0.9	0.9	0.9	0.7	0.6	0.8	0.8	0.0	0.9	0.9	0.9	0.9	0.9	0.9	0.9	0.9	0.9	0.8	0.9	0.9	0.8	0.6	0.5	0.9	0.9
M00847	Heme biosynthesis. archaea. siroheme => heme	0.7	1.0	1.0	0.7	1.0	0.7	0.7	0.3	0.7	1.0	0.0	1.0	1.0	1.0	0.7	1.0	0.3	0.3	0.0	0.0	0.3	1.0	0.7	1.0	0.3	0.7	1.0	0.0	1.0	0.7	0.0	1.0	1.0	1.0	1.0	1.0	1.0	1.0	1.0	1.0	1.0	1.0	1.0	1.0	1.0	0.7	0.0	0.0	0.3	0.7	1.0	1.0	1.0	0.7	0.7	0.7	0.7	0.7	0.7	1.0	0.7	1.0	0.0	0.0	0.7	1.0
M00848	Aurachin biosynthesis. anthranilate => aurachin A	0.0	0.0	0.0	0.0	0.0	0.0	0.0	0.0	0.0	0.0	0.0	0.0	0.0	0.0	0.0	0.0	0.0	0.0	0.0	0.0	0.0	0.0	0.0	0.0	0.0	0.0	0.0	0.0	0.0	0.0	0.0	0.0	0.0	0.0	0.0	0.0	0.0	0.0	0.0	0.0	0.0	0.0	0.0	0.0	0.0	0.0	0.0	0.0	0.0	0.0	0.0	0.0	0.0	0.0	0.0	0.0	0.0	0.0	0.0	0.0	0.0	0.0	0.0	0.0	0.0	0.0
M00849	C5 isoprenoid biosynthesis. mevalonate pathway. archaea	0.6	0.9	0.7	0.9	0.7	0.9	0.9	0.6	0.7	0.9	0.7	0.7	0.9	0.7	0.1	0.9	0.7	0.9	0.4	0.9	0.9	0.9	0.7	0.9	0.7	0.6	0.6	0.9	0.6	0.6	0.4	0.7	0.9	0.3	0.9	0.9	0.4	0.6	0.7	0.7	0.7	0.7	0.7	0.7	0.7	0.4	0.7	0.7	0.4	0.9	0.9	0.9	0.9	0.9	0.7	0.9	0.7	0.9	0.7	0.9	0.7	0.7	0.9	0.9	0.9	0.7
M00854	Glycogen biosynthesis. glucose-1P => glycogen/starch	0.3	0.3	0.3	0.3	0.7	0.3	0.0	0.0	0.0	0.3	0.0	0.0	0.0	0.0	0.3	0.7	0.7	0.7	0.3	0.0	0.0	0.3	0.3	0.0	0.3	0.3	0.0	0.3	0.3	0.3	0.3	0.3	0.3	0.0	0.3	0.3	0.7	0.3	0.3	0.7	0.0	0.0	0.0	0.0	0.3	0.0	0.3	0.3	0.0	0.3	0.3	0.3	0.0	0.3	0.3	0.0	0.3	0.0	0.3	0.3	0.0	0.3	0.3	0.0	0.3	0.0
M00855	Glycogen degradation. glycogen => glucose-6P	0.0	0.0	0.0	0.0	0.0	0.3	0.0	0.0	0.0	0.0	0.0	0.0	0.0	0.0	0.3	0.3	0.5	0.5	0.0	0.3	0.7	0.0	0.0	0.0	0.0	0.0	0.0	0.0	0.0	0.3	0.0	0.0	0.3	0.0	0.3	0.3	0.3	0.0	0.3	0.3	0.3	0.3	0.3	0.3	0.0	0.0	0.0	0.3	0.0	0.0	0.0	0.0	0.0	0.0	0.5	0.0	0.0	0.3	0.0	0.0	0.5	0.0	0.0	0.0	0.0	0.0
M00868	Heme biosynthesis. animals and fungi. glycine => heme	0.1	0.1	0.1	0.3	0.4	0.1	0.3	0.3	0.3	0.4	0.3	0.4	0.4	0.3	0.1	0.4	0.1	0.4	0.4	0.4	0.4	0.1	0.4	0.4	0.3	0.3	0.1	0.4	0.3	0.4	0.3	0.4	0.4	0.1	0.4	0.4	0.4	0.3	0.4	0.4	0.4	0.4	0.4	0.4	0.4	0.4	0.4	0.4	0.1	0.5	0.5	0.5	0.4	0.4	0.4	0.4	0.4	0.4	0.4	0.4	0.4	0.3	0.3	0.3	0.4	0.4
M00872	O-glycan biosynthesis. mannose type (core M3)	0.1	0.1	0.1	0.1	0.1	0.1	0.0	0.0	0.0	0.1	0.1	0.1	0.1	0.1	0.0	0.1	0.1	0.1	0.1	0.0	0.1	0.1	0.1	0.0	0.1	0.1	0.0	0.1	0.1	0.1	0.1	0.1	0.1	0.1	0.1	0.1	0.1	0.1	0.1	0.1	0.1	0.1	0.1	0.1	0.1	0.1	0.1	0.1	0.0	0.0	0.0	0.0	0.1	0.1	0.0	0.0	0.1	0.1	0.1	0.1	0.1	0.1	0.0	0.1	0.0	0.1
M00878	Phenylacetate degradation. phenylaxetate => acetyl-CoA/succinyl-CoA	0.3	0.3	0.1	0.3	0.1	0.1	0.4	0.3	0.4	0.4	0.1	0.1	0.1	0.1	0.0	0.3	0.1	0.3	0.4	0.1	0.3	0.3	0.3	0.1	0.3	0.0	0.0	0.1	0.4	0.4	0.3	0.1	0.4	0.1	0.3	0.3	0.3	0.4	0.4	0.4	0.3	0.3	0.3	0.3	0.3	0.0	0.6	0.6	0.3	0.3	0.3	0.3	0.0	0.0	0.1	0.1	0.0	0.0	0.0	0.0	0.0	0.0	0.0	0.1	0.0	0.1
M00880	Molybdenum cofactor biosynthesis. GTP => molybdenum cofactor	1.0	1.0	0.8	0.8	1.0	0.6	0.6	1.0	0.8	1.0	0.8	1.0	1.0	0.8	0.6	1.0	0.6	1.0	0.8	1.0	1.0	0.6	0.8	1.0	1.0	0.8	1.0	1.0	1.0	1.0	0.8	0.6	1.0	0.8	0.8	1.0	0.8	1.0	1.0	1.0	1.0	1.0	1.0	1.0	0.8	0.6	1.0	0.6	0.8	0.8	1.0	1.0	0.8	1.0	0.8	1.0	1.0	1.0	0.6	1.0	1.0	1.0	0.8	0.6	1.0	1.0
M00881	Lipoic acid biosynthesis. plants and bacteria. octanoyl-ACP => dihydrolipoyl-E2/H	0.0	0.0	0.0	0.0	0.0	0.0	0.0	0.0	0.0	0.0	0.0	0.0	0.0	0.0	0.0	0.0	0.0	0.0	0.0	0.0	0.0	0.0	0.0	0.0	0.0	0.0	0.0	0.0	0.0	0.0	0.0	0.0	0.0	0.0	0.0	0.0	0.0	0.0	0.0	0.0	0.0	0.0	0.0	0.0	0.0	0.0	0.0	0.0	0.0	0.0	0.0	0.0	0.0	0.0	0.0	0.0	0.0	0.0	0.0	0.0	0.0	0.0	0.0	1.0	0.0	0.0
M00882	Lipoic acid biosynthesis. eukaryotes. octanoyl-ACP => dihydrolipoyl-H	0.0	0.0	0.0	0.0	0.0	0.0	0.0	0.0	0.0	0.0	0.0	0.0	0.0	0.0	0.0	0.0	0.0	0.0	0.0	0.0	0.0	0.0	0.0	0.0	0.0	0.0	0.0	0.0	0.0	0.0	0.0	0.0	0.0	0.0	0.0	0.0	0.0	0.0	0.0	0.0	0.0	0.0	0.0	0.0	0.0	0.0	0.0	0.0	0.0	0.0	0.0	0.0	0.0	0.0	0.0	0.0	0.0	0.0	0.0	0.0	0.0	0.0	0.0	0.5	0.0	0.0
M00883	Lipoic acid biosynthesis. animals and bacteria. octanoyl-ACP => dihydrolipoyl-H => dihy	0.0	0.0	0.0	0.0	0.0	0.0	0.0	0.0	0.0	0.0	0.0	0.0	0.0	0.0	0.0	0.0	0.0	0.0	0.0	0.0	0.0	0.0	0.0	0.0	0.0	0.0	0.0	0.0	0.0	0.0	0.0	0.0	0.0	0.0	0.0	0.0	0.0	0.0	0.0	0.0	0.0	0.0	0.0	0.0	0.0	0.0	0.0	0.0	0.0	0.0	0.0	0.0	0.0	0.0	0.0	0.0	0.0	0.0	0.0	0.0	0.0	0.0	0.0	0.3	0.0	0.0
M00884	Lipoic acid biosynthesis. octanoyl-CoA => dihydrolipoyl-E2	0.0	0.0	0.0	0.0	0.0	0.0	0.0	0.0	0.0	0.0	0.0	0.0	0.0	0.0	0.0	0.0	0.0	0.0	0.0	0.0	0.0	0.0	0.0	0.0	0.0	0.0	0.0	0.0	0.0	0.0	0.0	0.0	0.0	0.0	0.0	0.0	0.0	0.0	0.0	0.0	0.0	0.0	0.0	0.0	0.0	0.0	0.0	0.0	0.0	0.0	0.0	0.0	0.0	0.0	0.0	0.0	0.0	0.0	0.0	0.0	0.0	0.0	0.0	0.5	0.0	0.0
M00892	UDP-N-acetyl-D-glucosamine biosynthesis. eukaryotes. glucose => UDP-GlcNAc	0.2	0.2	0.2	0.2	0.2	0.2	0.2	0.2	0.0	0.2	0.0	0.2	0.2	0.2	0.2	0.2	0.2	0.2	0.0	0.2	0.2	0.2	0.2	0.2	0.2	0.2	0.0	0.2	0.0	0.2	0.2	0.2	0.3	0.3	0.3	0.3	0.3	0.2	0.3	0.3	0.3	0.3	0.3	0.3	0.2	0.2	0.2	0.2	0.0	0.2	0.2	0.0	0.2	0.2	0.2	0.2	0.2	0.2	0.2	0.2	0.2	0.2	0.2	0.0	0.2	0.2
M00895	Thiamine biosynthesis. prokaryotes. AIR (+ DXP/glycine) => TMP/TPP	0.0	0.0	0.1	0.0	0.1	0.1	0.2	0.0	0.1	0.1	0.1	0.1	0.1	0.1	0.2	0.1	0.2	0.3	0.2	0.2	0.2	0.2	0.2	0.1	0.2	0.1	0.0	0.3	0.2	0.1	0.2	0.1	0.2	0.1	0.2	0.3	0.1	0.3	0.2	0.1	0.2	0.2	0.2	0.2	0.1	0.1	0.3	0.2	0.0	0.2	0.2	0.2	0.1	0.4	0.2	0.4	0.4	0.4	0.4	0.3	0.3	0.3	0.4	0.3	0.4	0.5
M00896	Thiamine biosynthesis. archaea. AIR (+ NAD+) => TMP/TPP	0.3	0.3	0.8	0.5	0.8	0.8	1.0	0.3	0.5	0.8	0.5	0.8	0.8	0.5	0.8	0.8	0.8	0.8	0.3	0.8	0.8	0.8	0.8	0.8	0.8	0.5	0.3	0.5	0.5	0.8	0.3	0.8	0.8	0.0	0.8	0.8	0.3	0.8	0.8	0.5	0.8	0.8	0.8	0.8	0.8	0.8	1.0	0.5	0.3	0.8	0.8	0.8	0.5	1.0	0.5	1.0	1.0	1.0	1.0	1.0	1.0	1.0	1.0	0.8	1.0	1.0
M00897	Thiamine biosynthesis. plants. AIR (+ NAD+) => TMP/thiamine/TPP	0.0	0.0	0.0	0.0	0.0	0.0	0.2	0.0	0.0	0.0	0.0	0.0	0.0	0.0	0.0	0.0	0.0	0.0	0.0	0.0	0.0	0.0	0.0	0.0	0.0	0.0	0.0	0.0	0.0	0.0	0.0	0.0	0.0	0.0	0.0	0.0	0.0	0.0	0.0	0.0	0.0	0.0	0.0	0.0	0.0	0.0	0.2	0.0	0.0	0.0	0.0	0.0	0.0	0.2	0.2	0.2	0.2	0.2	0.2	0.2	0.2	0.2	0.2	0.0	0.2	0.2
M00899	Thiamine salvage pathway. HMP/HET => TMP	0.0	0.5	0.5	0.5	0.5	0.5	0.5	0.0	0.0	0.5	0.5	0.5	0.5	0.5	0.5	0.5	0.5	0.5	0.5	0.5	0.5	0.5	0.5	0.5	0.5	0.5	0.5	0.5	0.0	0.5	0.0	0.5	0.5	0.0	0.5	0.5	0.5	0.5	0.5	0.5	0.5	0.5	0.5	0.5	0.5	0.5	0.5	0.0	0.0	0.5	0.5	0.5	0.0	0.5	0.0	0.5	0.5	0.5	0.5	0.5	0.5	0.5	0.5	0.5	0.5	0.5
M00909	UDP-N-acetyl-D-glucosamine biosynthesis. prokaryotes. glucose => UDP-GlcNAc	0.2	0.2	0.2	0.2	0.4	0.4	0.2	0.2	0.0	0.2	0.0	0.2	0.2	0.2	0.2	0.2	0.2	0.2	0.0	0.2	0.2	0.2	0.4	0.2	0.2	0.4	0.0	0.2	0.0	0.2	0.2	0.2	0.4	0.4	0.4	0.4	0.2	0.4	0.4	0.4	0.4	0.4	0.4	0.4	0.2	0.2	0.2	0.2	0.0	0.2	0.2	0.0	0.2	0.2	0.2	0.2	0.2	0.2	0.2	0.2	0.2	0.2	0.2	0.0	0.2	0.2
M00911	Riboflavin biosynthesis. fungi. GTP => riboflavin/FMN/FAD	0.1	0.1	0.1	0.2	0.2	0.2	0.2	0.2	0.2	0.2	0.2	0.2	0.2	0.1	0.2	0.2	0.2	0.2	0.0	0.2	0.2	0.2	0.2	0.1	0.2	0.2	0.1	0.2	0.1	0.2	0.1	0.1	0.2	0.2	0.1	0.2	0.2	0.2	0.1	0.2	0.2	0.2	0.2	0.1	0.2	0.2	0.2	0.1	0.1	0.1	0.2	0.2	0.2	0.2	0.2	0.2	0.2	0.2	0.2	0.1	0.2	0.2	0.2	0.2	0.2	0.2
M00912	NAD biosynthesis. tryptophan => quinolinate => NAD	0.3	0.3	0.3	0.3	0.1	0.3	0.3	0.3	0.1	0.3	0.1	0.3	0.3	0.1	0.1	0.1	0.4	0.3	0.3	0.3	0.4	0.1	0.4	0.4	0.1	0.1	0.3	0.4	0.3	0.4	0.1	0.4	0.1	0.1	0.1	0.1	0.3	0.0	0.1	0.1	0.3	0.3	0.3	0.3	0.3	0.3	0.3	0.1	0.3	0.4	0.1	0.3	0.3	0.4	0.4	0.4	0.4	0.4	0.4	0.3	0.4	0.3	0.3	0.3	0.4	0.4
M00913	Pantothenate biosynthesis. 2-oxoisovalerate/spermine => pantothenate	0.0	0.0	0.0	0.0	0.0	0.0	0.2	0.2	0.2	0.2	0.2	0.2	0.0	0.0	0.2	0.2	0.2	0.2	0.0	0.0	0.0	0.2	0.0	0.0	0.0	0.0	0.0	0.2	0.2	0.2	0.2	0.2	0.2	0.0	0.0	0.0	0.0	0.0	0.0	0.2	0.2	0.2	0.2	0.2	0.2	0.2	0.2	0.2	0.2	0.2	0.2	0.2	0.0	0.2	0.2	0.0	0.2	0.0	0.2	0.0	0.2	0.0	0.2	0.0	0.2	0.0
M00914	Coenzyme A biosynthesis. archaea. 2-oxoisovalerate => 4-phosphopantoate => CoA	0.6	0.4	0.6	0.6	0.6	0.4	0.4	0.7	0.6	0.4	0.3	0.6	0.6	0.4	0.4	0.7	0.6	0.6	0.1	0.6	0.6	0.4	0.6	0.6	0.4	0.6	0.4	0.6	0.6	0.6	0.6	0.7	0.7	0.4	0.6	0.4	0.6	0.6	0.4	0.7	0.7	0.7	0.7	0.7	0.7	0.4	0.6	0.6	0.6	0.9	0.9	0.9	0.3	0.9	0.3	0.7	0.9	0.7	0.6	0.7	0.9	0.6	0.7	0.7	0.9	0.7
M00916	Pyridoxal-P biosynthesis. R5P + glyceraldehyde-3P + glutamine => pyridoxal-P	1.0	1.0	1.0	1.0	1.0	0.5	1.0	1.0	1.0	0.5	1.0	0.5	0.5	0.5	0.0	0.5	1.0	1.0	0.5	0.5	1.0	0.5	1.0	1.0	1.0	0.5	0.0	0.5	0.5	1.0	1.0	1.0	1.0	0.5	1.0	1.0	1.0	1.0	1.0	1.0	0.5	0.5	0.5	1.0	1.0	0.0	1.0	1.0	0.0	1.0	1.0	1.0	0.5	1.0	1.0	1.0	1.0	1.0	1.0	1.0	1.0	1.0	0.5	0.0	1.0	1.0
M00921	Cyclooctatin biosynthesis. dimethylallyl-PP + isopentenyl-PP => cyclooctatin	0.0	0.0	0.0	0.0	0.0	0.0	0.0	0.0	0.0	0.0	0.0	0.0	0.0	0.0	0.0	0.0	0.0	0.3	0.0	0.3	0.3	0.3	0.3	0.0	0.3	0.0	0.3	0.0	0.0	0.0	0.0	0.0	0.3	0.3	0.3	0.3	0.3	0.3	0.3	0.3	0.3	0.3	0.3	0.3	0.0	0.0	0.0	0.0	0.0	0.0	0.0	0.0	0.0	0.0	0.0	0.0	0.0	0.0	0.0	0.0	0.0	0.0	0.0	0.3	0.0	0.3
M00922	CMP-Neu5Ac biosynthesis	0.0	0.0	0.0	0.0	0.0	0.0	0.0	0.0	0.0	0.0	0.0	0.0	0.0	0.0	0.0	0.0	0.0	0.0	0.0	0.0	0.0	0.0	0.0	0.0	0.3	0.0	0.0	0.0	0.3	0.3	0.3	0.0	0.0	0.0	0.0	0.0	0.0	0.0	0.0	0.0	0.0	0.0	0.0	0.0	0.0	0.0	0.0	0.0	0.0	0.0	0.0	0.0	0.0	0.0	0.0	0.0	0.0	0.0	0.0	0.0	0.0	0.0	0.0	0.0	0.0	0.0
M00923	UDP-L-FucNAm biosynthesis	0.0	0.0	0.0	0.0	0.0	0.0	0.0	0.0	0.0	0.0	0.0	0.0	0.0	0.0	0.0	0.0	0.0	0.0	0.0	0.0	0.0	0.0	0.0	0.0	0.3	0.3	0.0	0.0	0.0	0.3	0.0	0.3	0.0	0.0	0.0	0.0	0.0	0.0	0.0	0.0	0.0	0.0	0.0	0.0	0.0	0.0	0.0	0.0	0.0	0.0	0.0	0.0	0.0	0.0	0.0	0.0	0.0	0.0	0.0	0.0	0.0	0.0	0.0	0.0	0.0	0.0
M00924	Cobalamin biosynthesis. anaerobic. uroporphyrinogen III => sirohydrochlorin => cobyrina	0.5	0.8	0.7	0.8	0.3	0.3	0.6	0.5	0.5	0.7	0.4	0.4	0.3	0.2	0.3	0.8	0.3	0.8	0.3	0.8	0.3	0.6	0.7	0.7	0.7	0.4	0.3	0.3	0.8	0.5	0.6	0.7	0.8	0.4	0.2	0.3	0.3	0.3	0.3	0.8	0.6	0.6	0.6	0.6	0.5	0.7	0.3	0.7	0.0	0.4	0.3	0.7	0.3	0.8	0.8	0.7	0.7	0.6	0.8	0.8	0.5	0.8	0.7	0.6	0.8	0.8
M00925	Cobalamin biosynthesis. aerobic. uroporphyrinogen III => precorrin 2 => cobyrinate a.c-d	0.3	0.5	0.4	0.5	0.2	0.2	0.5	0.3	0.3	0.5	0.3	0.3	0.2	0.1	0.2	0.5	0.2	0.5	0.2	0.5	0.2	0.4	0.5	0.5	0.5	0.3	0.1	0.2	0.6	0.4	0.3	0.5	0.5	0.3	0.2	0.2	0.2	0.1	0.2	0.5	0.4	0.4	0.4	0.4	0.3	0.4	0.2	0.5	0.0	0.3	0.2	0.5	0.2	0.6	0.6	0.4	0.4	0.4	0.6	0.6	0.4	0.6	0.4	0.4	0.5	0.6
M00926	Heme biosynthesis. bacteria. glutamyl-tRNA => coproporphyrin III => heme	0.2	0.3	0.2	0.4	0.6	0.3	0.4	0.2	0.4	0.6	0.4	0.6	0.6	0.3	0.2	0.6	0.1	0.6	0.6	0.6	0.6	0.3	0.6	0.6	0.4	0.3	0.3	0.4	0.3	0.4	0.4	0.4	0.6	0.2	0.4	0.6	0.6	0.4	0.6	0.4	0.6	0.6	0.6	0.6	0.4	0.3	0.6	0.6	0.1	0.7	0.7	0.7	0.6	0.6	0.6	0.6	0.6	0.6	0.4	0.6	0.6	0.4	0.3	0.3	0.6	0.6
M00930	Menaquinone biosynthesis. futalosine pathway	0.0	0.5	0.7	0.7	0.5	0.7	0.3	0.0	0.2	0.7	0.2	0.7	0.7	0.5	0.2	0.7	0.0	0.7	0.7	0.0	0.7	0.0	0.7	0.7	0.5	0.7	0.7	0.5	0.7	0.7	0.3	0.2	0.3	0.3	0.7	0.7	0.0	0.5	0.7	0.7	0.7	0.7	0.7	0.7	0.7	0.2	0.0	0.0	0.0	1.0	1.0	1.0	0.2	0.0	0.0	0.0	0.0	0.0	0.0	0.8	0.0	0.8	0.0	0.0	0.0	0.0
M00931	Menaquinone biosynthesis. modified futalosine pathway	0.0	0.6	0.8	0.8	0.6	0.8	0.4	0.0	0.2	0.8	0.2	0.8	0.8	0.6	0.2	0.8	0.0	0.8	0.8	0.0	0.8	0.0	0.8	0.8	0.6	0.8	0.8	0.6	0.8	0.8	0.4	0.2	0.4	0.4	0.8	0.8	0.0	0.6	0.8	0.8	0.8	0.8	0.8	0.8	0.8	0.2	0.0	0.0	0.0	0.8	0.8	0.8	0.2	0.0	0.0	0.0	0.0	0.0	0.0	0.8	0.0	0.8	0.0	0.0	0.0	0.0
M00935	Methanofuran biosynthesis	0.8	0.8	0.8	0.8	0.8	0.8	0.7	0.7	0.7	0.8	0.8	0.8	0.8	0.3	0.2	0.8	0.7	0.8	0.7	0.7	0.8	0.7	0.7	0.5	0.5	0.0	0.5	0.7	0.3	0.8	0.7	0.8	0.8	0.7	0.8	0.8	0.7	0.7	0.8	0.8	0.8	0.8	0.8	0.8	0.7	0.7	0.7	0.3	0.3	0.8	0.8	0.8	0.8	0.8	0.8	1.0	0.8	0.5	0.8	0.8	0.8	0.7	0.8	0.5	0.7	1.0
M00938	Pyrimidine deoxyribonuleotide biosynthesis. UDP => dTTP	0.5	0.6	0.6	0.9	0.6	0.5	0.9	0.6	0.4	0.9	0.6	0.5	0.8	0.8	0.6	0.8	0.7	0.7	0.7	0.3	0.7	0.4	0.7	0.8	0.7	0.9	0.8	0.8	0.4	0.8	0.8	0.8	0.9	0.9	0.9	0.9	0.5	0.6	0.6	0.6	0.6	0.6	0.6	0.6	0.6	0.8	0.8	0.5	0.0	0.9	0.9	0.9	0.5	0.4	0.3	0.9	0.3	0.4	0.3	0.9	0.3	0.6	0.3	0.7	0.4	0.6
