# Supplementary material for: Phylogenetic and functional diverse ANME-1 thrive in Arctic hydrothermal vents
Source: FEMS Microbiol Ecol. 2022 Oct 3;98(11):fiac117. doi: 10.1093/femsec/fiac117 (PMC9576274; doi:10.1093/femsec/fiac117)

Supplementary Table 8: List of enriched functions in generalist and vent-specific groups. Putative functions, GO biological process, COG category and cellular location are given, when available. For the generalist group, the histogram to the right represents a summary of enriched COG categories.

	Putative function	GO Biological processes	COG categories	Cellular location	# in Figure 3
**Generalist**					
tellurite methyltransferase [EC:2.1.1.265]	tellurium detoxification	response to toxic substance, response to antibiotic			1
arsenite methyltransferase [EC:2.1.1.137]	arsenic methylation, common in marise anaerobic sediments	conversion of As(III) to a number of methylated products, Reduces the arsenic toxicity			2
multidrug resistance protein, MATE family	efflux of antimicrobial compound	xenobiotic transport	?	membrane	3
HTH-type transcriptional regulator, sugar sensing transcriptional regulator	transcriptional regulator	uptake and catabolism of malto-oligosaccharides, positive regulation of carbohydrate metabolic process	K	membrane	4
peptidoglycan-N-acetylglucosamine deacetylase [EC:3.5.1.104]	membrane modification	cell wall modification, deacetylation of N-acetylglucosamine (GlcNAc) residues in peptidoglycan, confers host lysozyme resistance	G	membrane	5
lipooligosaccharide transport system ATP-binding protein		ABC transporter complex NodIJ involved in the export of the nodulation factors (Nod factors), the bacterial signal molecules that induce symbiosis, This subunit is responsible for energy coupling to the transport system	V	membrane	6
lipooligosaccharide transport system permease protein		ABC transporter complex NodIJ involved in the export of the nodulation factors (Nod factors), the bacterial signal molecules that induce symbiosis, This subunit is responsible for energy coupling to the transport system	V	membrane	7
TrkA domain protein	signaling and cellular processes	potassium transport systems	P	membrane	8

protein O-mannosyl-transferase [EC:2.4.1.-]	O-Glycan biosynthesis	protein O-linked mannosylation	O	membrane	9
membrane protein HdeD	The hdeD Gene Represses the Expression of Flagella	response to pH	U	membrane	10
putative dimethyl sulfoxide reductase chaperone	chaperone	assembly of dimethyl sulfoxide (DMSO) reductase	?	?	11
molecular chaperone GrpE		hyperosmotic and heat shock, protein folding, response to heat	O	cytoplasm	12
heat shock protein HslJ	Heat shock proteins	response to heat	O		13
ATP-dependent Clp protease ATP-binding subunit ClpB	Heat shock proteins	directs the protease to specific substrates; hydrolysis to unfold proteins	O	cytoplasm	14
two-component system, chemotaxis family, sensor kinase CheA [EC:2.7.13.3]	chemotaxis	chemotaxis	T	cytoplasm	15
two-component system, sensor histidine kinase PdtaS [EC:2.7.13.3]	The genes downregulated in the Δms*pdtaS* strain belong to the Fe-S cluster protein family, hydrolases, oxidoreductases, transmembrane transport, and nitrogen assimilation genes. Genes upregulated affect the processes of nucleotide biosynthesis, DNA maintenance, amino acid biosynthesis, co-factor biosynthesis, fatty acid biosynthesis, ribosomal protein biosynthesis, and tRNA synthetases and transferases	phosphorelay signal transduction system, protein autophosphorylation	T		16
bacterioferritin [EC:1.16.3.1]	to store iron in the ferric form.	ferric iron binding	P	Cytosol/membrane	17
mRNA interferase RelE/StbE	mRNA interferase	cellular response to amino acid starvation	DJ	protein-DNA complex	18
circadian clock protein KaiC	Circadian (daily) biological clocks	regulation of circadian rhythm	T		19
small conductance mechanosensitive channel	opening in response to stretching of the cell membrane	cellular water homeostasis	M	membrane	20
catalase [EC:1.11.1.6]	H2O2 --> O2	cellular response to hydrogen peroxide	P		21
inhibitor of cysteine peptidase		?	?	?	22
CRISPR-associated protein Cst2	CRISPR	defense response to virus	L	?	23
type I restriction enzyme, S subunit [EC:3.1.21.3]		DNA restriction-modification system	L		24
type III restriction enzyme [EC:3.1.21.5]		DNA restriction-modification system			25
ribonuclease HI [EC:3.1.26.4]		?	?	?	26
carboxypeptidase T [EC:3.4.17.18]	Metallo peptidases	metallocarboxypeptidase activity	?	?	27
micrococcal nuclease [EC:3.1.31.1]	digest DNA/RNA	?		?	28
antitoxin PrlF	Type II TA system	?		?	29
tRNA(fMet)-specific endonuclease VapC [EC:3.1.-.-]	Type II TA system	?		?	30
adenine-specific DNA-methyltransferase [EC:2.1.1.72]	Type II R-M system	defense response to virus		?	31

mRNA interferase MazF [EC:3.1.-.-]	mRNA interferase	defense response to virus, regulation of translation, rRNA catabolic process, quorum sensing	T	protein containing complex	32
phenylacetate-CoA ligase [EC:6.2.1.30]	phenylacetate degradation	phenylacetate catabolic process	H	?	
N-methylhydantoinase B [EC:3.5.2.14]	converts hydantoin to N-carbamyl-amino acids	glutathione metabolic process/creatinine pathway	?	cytosol	
U32 family peptidase [EC:3.4.-.-]	have been linked to pathogenicity	tRNA modification	O	cytoplasm	
N-methylhydantoinase A [EC:3.5.2.14]	converts hydantoin to N-carbamyl-amino acids				
ferrous-iron efflux pump FieF	FieF (YiiP) from Escherichia coli mediates decreased cellular accumulation of iron and relieves iron stress	zinc, cadmium, iron homeostasis	P	membrane	
malate permease and related proteins (K24180)	malate transporter	transmembrane transport	S	membrane	
acetyl-CoA synthetase [EC:6.2.1.1]					
anaerobilin synthase [EC:2.1.1.342]	oxygen-independent heme-degradation pathway				
5-methylcytosine-specific restriction enzyme A [EC:3.1.21.-]	Type IV R-M system				
TolB protein	maybe porine assembly	cell cycle, protein transport	U	periplasm	
flavodoxin I	electron-transfer proteins	iron-sulfur cluster assembly, response to superoxide, response to drug	C	cytoplasm	
UTP--glucose-1-phosphate uridylyltransferase [EC:2.7.7.9]	UDP-glucpse biosynthesis	galactose catabolic process via UDP-galactose, lipopolysaccharide core region biosynthetic process, osmoregulated periplasmic glucan biosynthetic process, UDP-glucose metabolic process	M	protein containing complex, protein containing complex	
cobalt-precorrin-5B (C1)-methyltransferase [EC:2.1.1.195]	anaerobic biosynthesis of cobalamin/vitamin b12	cobalamin biosynthetic process, corrin biosynthetic process, cobalt ion transport	?	membrane	
phosphate transport system protein		cellular phosphate ion homeostasis, cellular response to antibiotic, cellular response to heat, cellular response to pH, cellular response to phosphate starvation, negative regulation of gene expression, negative regulation of ion transmembrane transporter activity, regulation of cell growth	P	cytoplasm	
glucose-6-phosphate isomerase, archaeal [EC:5.3.1.9]	glucose <--> fructose	gluconeogenesis, glycolytic process		cytoplasm	
2'-deoxynucleoside 5'-phosphate N-hydrolase [EC:3.2.2.-]	?	deoxyribonucleoside monophosphate catabolic process, nucleoside metabolic process, positive regulation of cell growth		cytoplasm	

cysteinyl-tRNA synthetase [EC:6.1.1.16]		cysteinyl-tRNA aminoacylation	J	cytoplasm	
uridine phosphorylase [EC:2.4.2.3]	uridina <--> uracile	cellular response to DNA damage stimulus, nucleotide catabolic process	F	cytoplasm	
(aminoalkyl)phosphonate N-acetyltransferase [EC:2.3.1.280]	phosphonate metabolism	transcription	K	?	
acylaminoacyl-peptidase [EC:3.4.19.1]	The products of the reaction are an acyl amino acid and a peptide with a free N terminus shortened by one amino acid	amyloid-beta metabolic process, proteolysis	E	cytoplasm	
Ca2+/H+ antiporter, TMEM165/GDT1 family	Ca2+/H+ antiporter	?	?	membrane	
NADH-dependent peroxiredoxin subunit F [EC:1.8.1.-]					
acid phosphatase type 7					
putative phosphoribosyl transferase					
acyl-CoA thioesterase [EC:3.1.2.-]		acyl-CoA hydrolase activity	?	cytoplasm	
precorrin-2/cobalt-factor-2 C20-methyltransferase [EC:2.1.1.130 2.1.1.151]		cobalamin biosynthetic process	?	?	
arylformamidase [EC:3.5.1.9]		anthranilate metabolic process, tryptophan catabolic process to kynurenine	S	?	
ATP citrate (pro-S)-lyase [EC:2.3.3.8]		acetyl-CoA biosynthetic process	C	cytoplasm	
acetyltransferase					
insertion element IS1 protein InsB		transposition, DNA-mediated	?	?	
2-iminobutanoate/2-iminopropanoate deaminase [EC:3.5.99.10]	Hydrolases	isoleucine biosynthetic process; response to toxic substance	J	cytoplasm	
ATP-dependent Clp protease ATP-binding subunit ClpB	Heat shock proteins	directs the protease to specific substrates; hydrolysis to unfold proteins	O	cytoplasm	
formylmethanofuran dehydrogenase subunit E [EC:1.2.7.12]		4 iron, 4 sulfur cluster binding	C		
thiosulfate/3-mercaptopyruvate sulfurtransferase [EC:2.8.1.1 2.8.1.2]	thiosulfate --> sulfite	sulfurtransferase activity, thiosulfate sulfurtransferase activity, thiosulfate sulfurtransferase activity	P		
D-alanine-D-alanine ligase [EC:6.3.2.4]		peptidoglycan biosynthetic process, D-alanine-D-alanine ligase activity	F	cytoplasm	
DeoR family transcriptional regulator, suf operon transcriptional repressor		Negative regulator of the dra-nupC-pdp operon	K		
thiosulfate reductase / polysulfide reductase chain A [EC:1.8.5.5]	sulfide --> thiosulfate	oxidoreductase activity, Transporters			
anti-sigma B factor antagonist		Positive regulator of sigma-B activity	T		
beta-phosphoglucomutase [EC:5.4.2.6]		carbohydrate metabolic process	S	cytoplasm	
ATP-citrate lyase beta-subunit [EC:2.3.3.8]		acetyl-CoA biosynthetic process	C	cytoplasm	

manganese-dependent inorganic pyrophosphatase [EC:3.6.1.1]		polyphosphate catabolic process	C	cytoplasm	
RNA-binding protein					
16S rRNA (adenine1518-N6/adenine1519-N6)-dimethyltransferase [EC:2.1.1.182]		Specifically dimethylates two adjacent adenosines, response to antibiotic, ribosomal small subunit assembly, rRNA methylation	J	cytoplasm	
hydrogenase expression/formation protein HypD	necessary for hydrogenase activity	Involved in the maturation of [NiFe] hydrogenase, cellular protein modification process	O	?	
DNA repair protein RadC		Involved in DNA double-strand break (DSB) repair and recombination, DNA recombinase assembly	L		
tubulin-like protein CetZ		regulation of cell shape, Essential for the development of a rod-shaped cell type required for efficient swimming	D	cytoplasm	
DNA polymerase IV [EC:2.7.7.7]		It is required for stationary-phase adaptive mutation, which provides the bacterium with flexibility in dealing with environmental stress, enhancing long-term survival and evolutionary fitness, Poorly processive, error-prone DNA polymerase	L	cytoplasm	
phytol kinase [EC:2.7.1.182]		Transferring phosphorus-containing groups, vitamin E biosynthetic process	I		
S-adenosylmethionine decarboxylase [EC:4.1.1.50]					
inner membrane protein					
energy-converting hydrogenase B subunit D	hydrogenase?energy converting?	Unclassified: metabolism	P	membrane	
glucosylglycerate synthase [EC:2.4.1.268]		biosynthesis of the compatible solute mannosylglucosylglycerate	M		
alanine or glycine:cation symporter, AGCS family		TC.AGCS; alanine or glycine:cation symporter, AGCS family			
gamma-glutamyl hercynylcysteine S-oxide synthase [EC:1.14.99.50]		ergothioneine biosynthesis, which is part of Amino-acid biosynthesis	T		
formate dehydrogenase major subunit [EC:1.17.1.9]					
5-methylcytosine-specific restriction enzyme A [EC:3.1.21.-]	Type IV R-M system				
diaminopimelate dehydrogenase [EC:1.4.1.16]		Probably plays a role in lysine biosynthesis			

carbamate kinase [EC:2.7.2.2]		Can also use acetate, arginine metabolic process, carbamate kinase activity		cytoplasm	
sugar fermentation stimulation protein A	Could be a regulatory factor involved in maltose metabolism.	Binds to DNA non-specifically. Could be a regulatory factor involved in maltose metabolism	S		

Vent-specific

hydroxymethylglutaryl-CoA reductase (NADPH) [EC:1.1.1.34]	mevolonate-isoprenyl synthesis	Catalyzes the synthesis of mevalonate, the specific precursor of all isoprenoid compounds, coenzyme A metabolic process, triterpenoid biosynthetic process		Membrane
tRNA (guanine6-N2)-methyltransferase [EC:2.1.1.256]	thermostability of archaeal tRNA	methylation of the guanosine nucleotide at position 6, tRNA processing	L	Cytoplasm
NAD+ synthase (glutamine-hydrolysing) [EC:6.3.5.1]		NAD biosynthetic process	H	Cytoplasm
predicted type IV restriction endonuclease				
anaerobic carbon-monoxide dehydrogenase iron sulfur subunit				
4-alpha-glucanotransferase [EC:2.4.1.25]	glycogen --> glucose	transglycosylation of maltooligosaccharides, yielding maltooligosaccharides of various lengths and glucose		
glucoamylase [EC:3.2.1.3]	starch;glycogen --> glucose	Hydrolysis of terminal (1->4)-linked alpha-D-glucose	G	
23S rRNA (cytidine1920-2'-O)/16S rRNA (cytidine1409-2'-O)-methyltransferase [EC:2.1.1.226 2.1.1.227]	These methylations result in increased susceptibility to the antibiotics capreomycin and viomycin.	23S rRNA modification factors, methylation		
acetoin utilization protein AcuC	Acetoin metabolite	Part of the acuABC operon, which is possibly involved in the breakdown of acetoin and butanediol, sporulation resulting in formation of a cellular spore	K	
DNA-directed RNA polymerase subunit D [EC:2.7.7.6]				
phosphonate transport system substrate-binding protein	phosphonate antibiotic	Part of the ABC transporter complex PhnCDE involved in phosphonates import	P	Membrane

N-glycosylase/DNA lyase [EC:3.2.2.- 4.2.99.18]				
P-type Ca2+ transporter type 2C [EC:7.2.2.10]	Catalysing the translocation of inorganic cations			
CRISPR-associated protein Csm1				
tRNA(His) guanylyltransferase [EC:2.7.7.79]	In eukarya an additional guanosine residue is added post-transcriptionally to the 5'-end of tRNAHis molecules			
F420H2:quinone oxidoreductase subunit L [EC:1.1.98.4]				

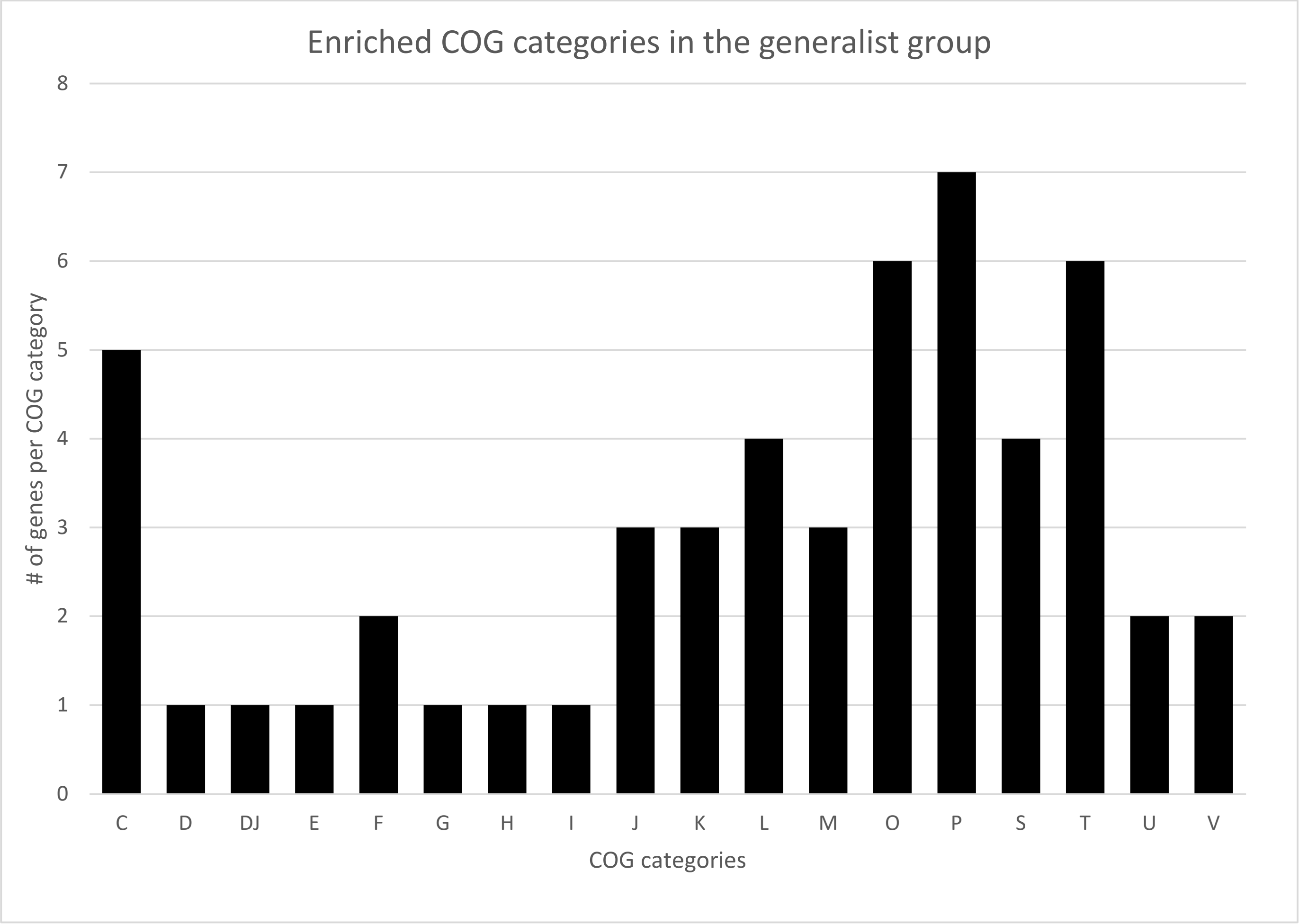

Supplement: fiac117_Supplemental_Files [file fiac117_supplemental_files.zip › Supp_data_Table_8.pdf]
