# Supplementary figures and images for: Phylogenetic and functional diverse ANME-1 thrive in Arctic hydrothermal vents
Source: FEMS Microbiol Ecol. 2022 Oct 3;98(11):fiac117. doi: 10.1093/femsec/fiac117 (PMC9576274; doi:10.1093/femsec/fiac117)

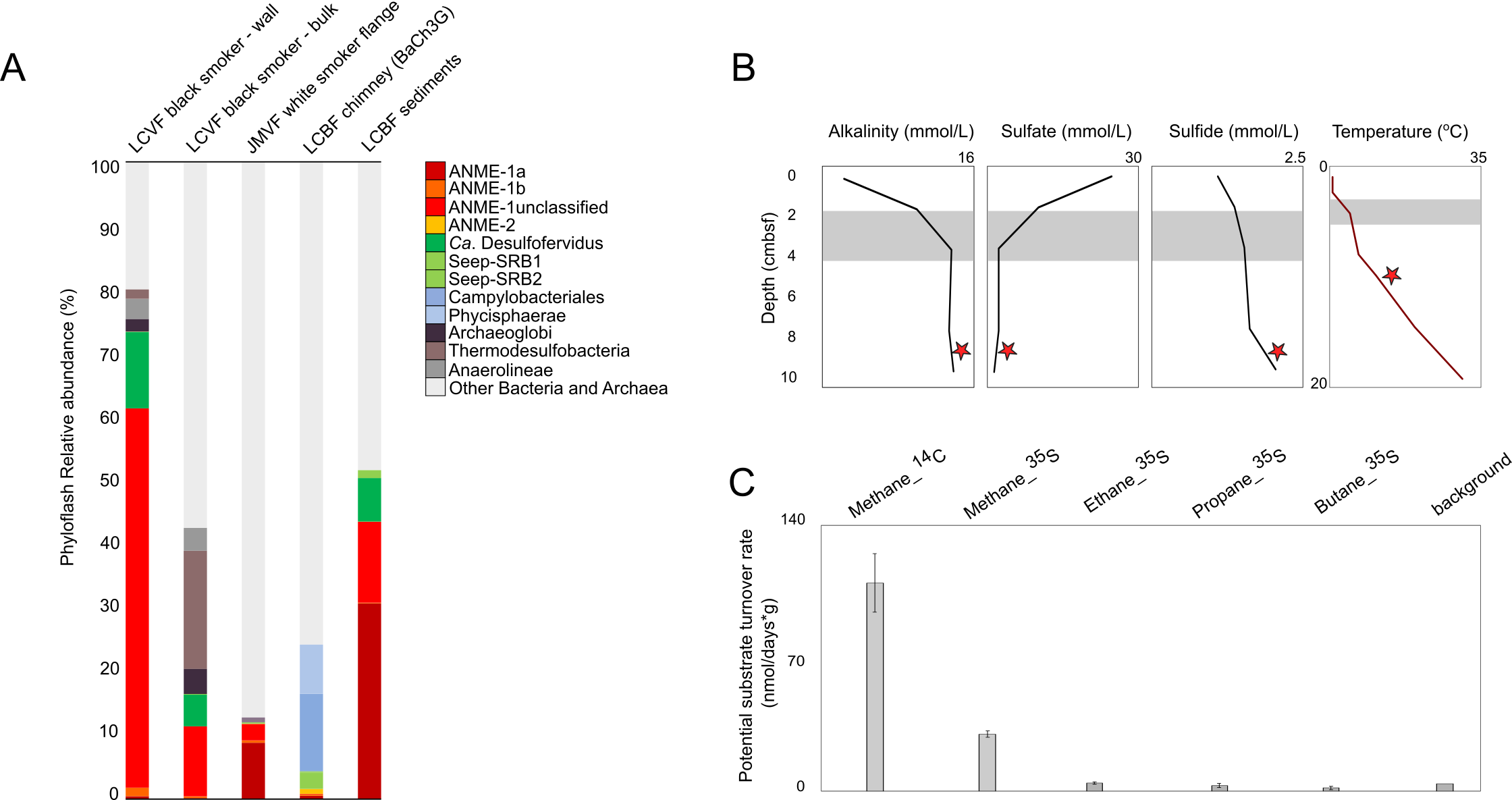

Supplement: fiac117_Supplemental_Files [file fiac117_supplemental_files.zip › Supp_data_Figure1ABC.tif]

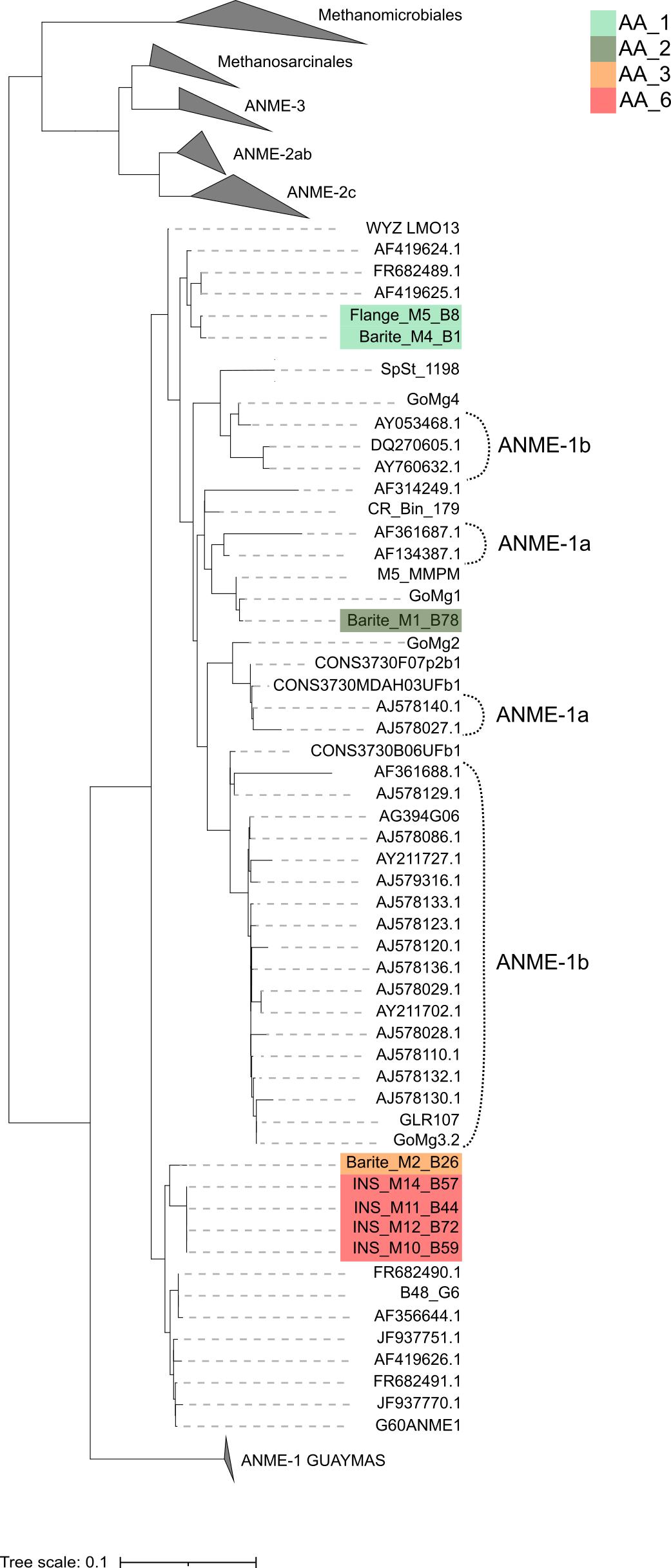

Supplement: fiac117_Supplemental_Files [file fiac117_supplemental_files.zip › Supp_data_Figure2.tif]

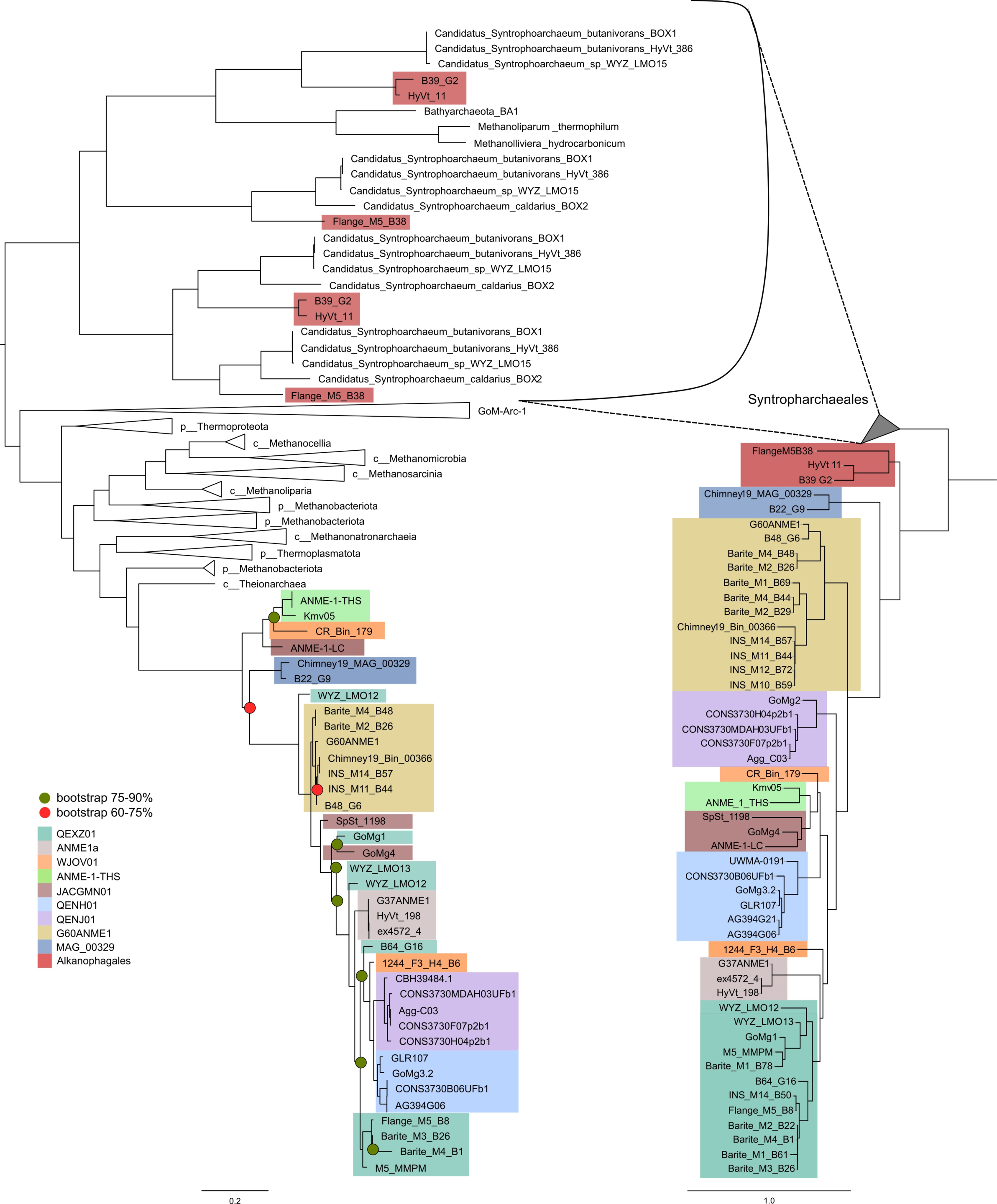

Supplement: fiac117_Supplemental_Files [file fiac117_supplemental_files.zip › Supp_data_Figure3.tif]

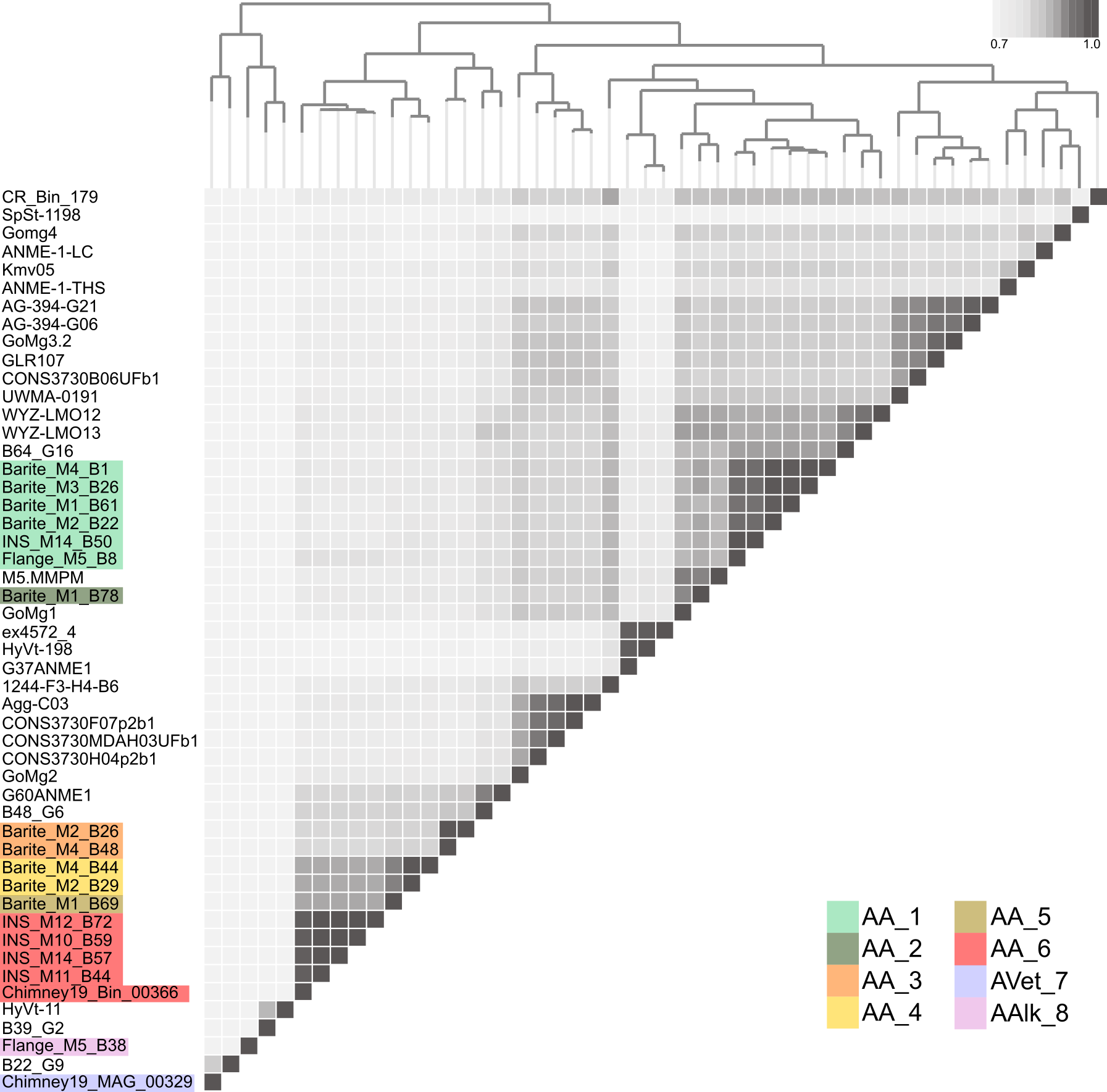

Supplement: fiac117_Supplemental_Files [file fiac117_supplemental_files.zip › Supp_data_Figure4.tif]

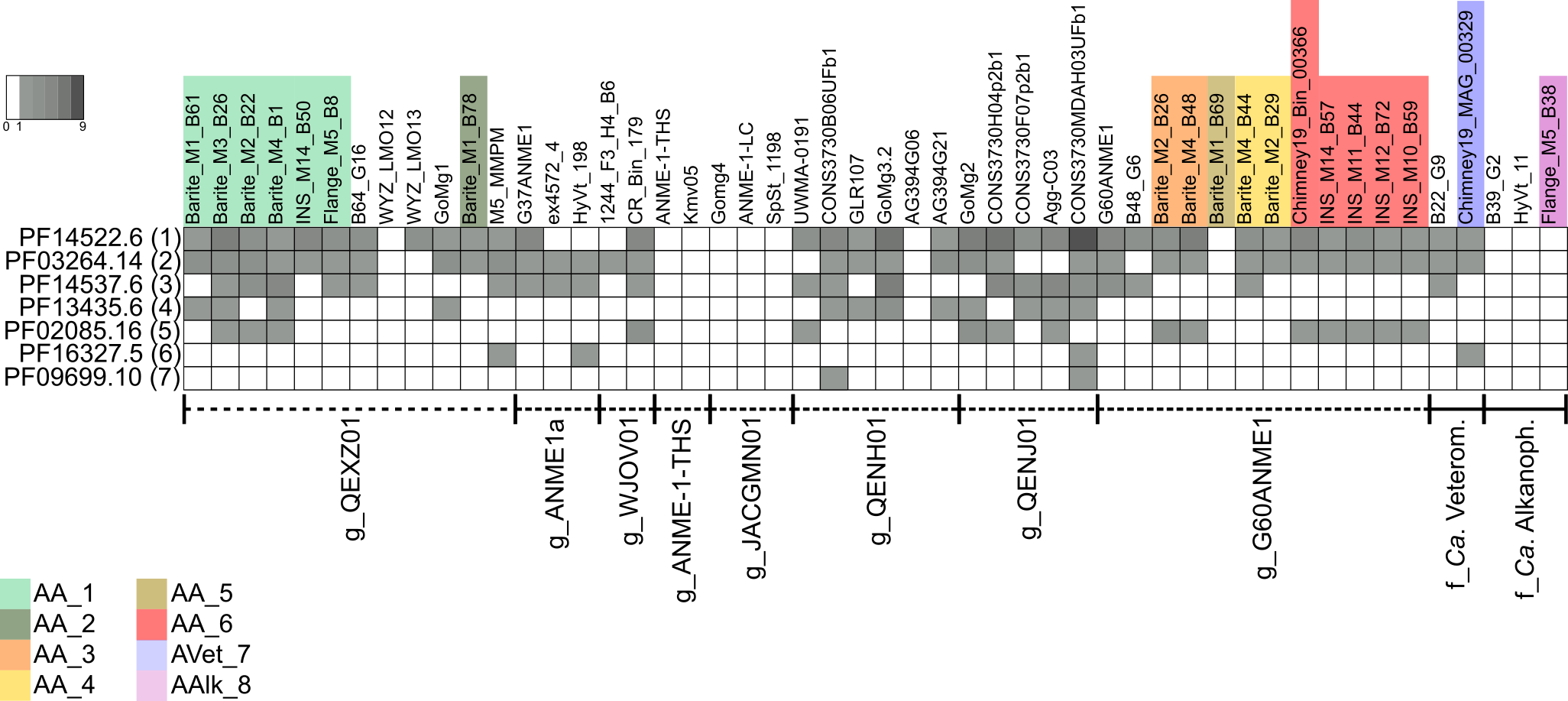

Supplement: fiac117_Supplemental_Files [file fiac117_supplemental_files.zip › Supp_data_Figure5.tif]

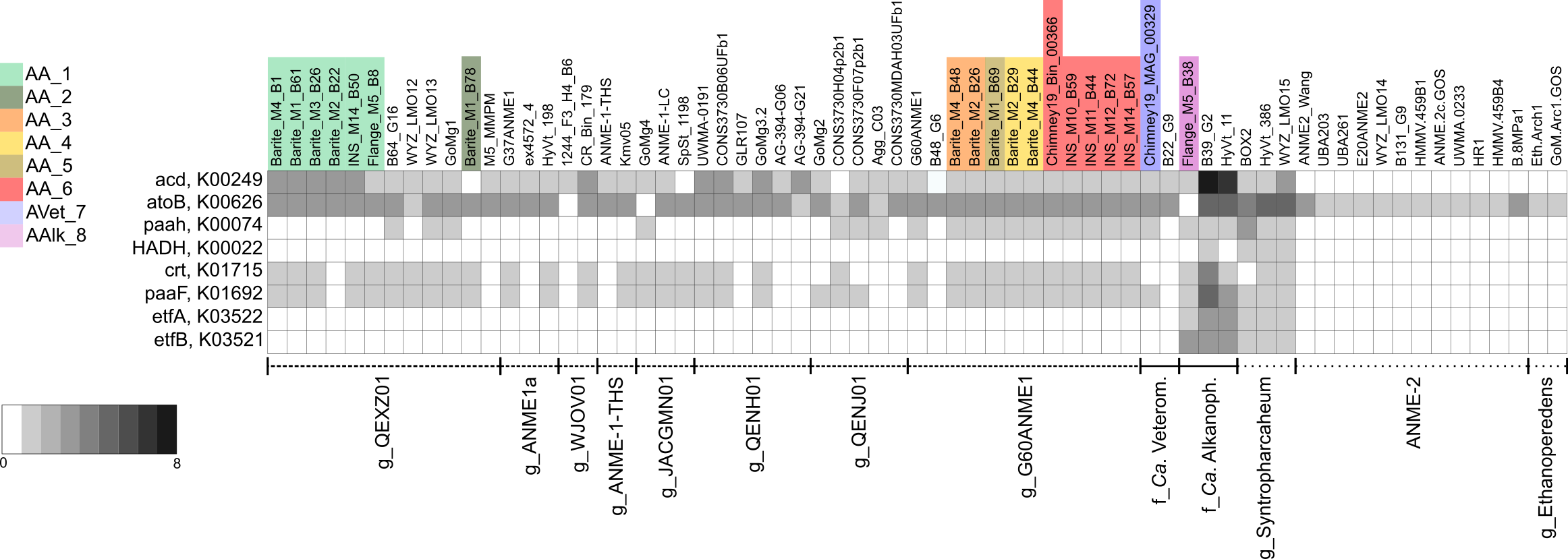

Supplement: fiac117_Supplemental_Files [file fiac117_supplemental_files.zip › Supp_data_Figure6.tif]
